# Solvent-Dependent Ultrafast Photochemical Dynamics of *N*‑Methyl Oxindole Overcrowded Alkene Molecular Motors

**DOI:** 10.1021/acs.jpca.5c02679

**Published:** 2025-06-16

**Authors:** Connah J. Harris, Beatrice S. L. Collins, Andrew J. Orr-Ewing

**Affiliations:** 1980 School of Chemistry, University of Bristol, Cantock’s Close, Bristol BS8 1TS, United Kingdom

## Abstract

Overcrowded alkenes are a class of rotational molecular motors that operate via alternating photochemical and thermal relaxation processes. Although the performances of various designs of molecular motors have been extensively studied, in general, their photoinduced isomerization efficiencies remain low. Ultrafast time-resolved spectroscopy can explore the excited-state dynamics and investigate the photoisomerization mechanisms. Herein, we study a series of visible-light-activated overcrowded alkene motors with *N*-methyl oxindole functionality using transient absorption and time-resolved infrared (TRIR) spectroscopies. The motors are examined in cyclohexane, DMSO, and methanol to probe the solvent environmental effects on the photoisomerization, paying particular attention to polarity and viscosity. Four dynamical processes are identified: relaxation from the Franck–Condon region of the bright excited state to a region of different electronic character (<120 fs) that is not directly optically accessible from the ground state; prompt (0.5–4 ps) and indirect (4–14 ps) depopulation of this dark state via conical intersections with the ground state; and vibrational cooling of hot ground-state molecules (10–15 ps). The time scales for decay of the dark state are both solvent polarity and viscosity-dependent. In nonpolar cyclohexane solutions, only direct depopulation of the dark state is observed, but in the DMSO and methanol solutions, both prompt and indirect depopulation are identified. Greater solvent viscosity increases the average excited-state lifetimes of the dark states by inhibiting rotation of the alkene bond. Oscillations observed in the excited-state absorption bands are attributed to coherent vibrations of the excited-state wave packet. Density functional theory (DFT) calculations of the stable (*P*,*P*)-*E* and metastable (*M*,*M*)-*Z* diastereomer structures, optimized at the ωB97XD/6–31+G­(d,p) level of theory and interpolated between the two geometries using internal coordinates, are used to approximate the geometrical change of the isomerization reaction in the excited state. For each interpolated structure, the vertical excitation energies are calculated using time-dependent DFT calculations at the same level of theory to track the adiabatic potential energy surfaces of the S_0_, S_1_, and S_2_ electronic states. This interpolation study shows that the excited-state dynamics are dictated by the S_1_ state, with no involvement of higher-lying singlet states. The poor quantum yield of isomerization is confirmed using the degree of ground-state bleach recovery of the carbonyl stretch in the recorded TRIR spectra, finding an upper estimate of the quantum yield for isomerization of the five molecular motors studied to range from 0.4–8.7%.

## Graphical abstract

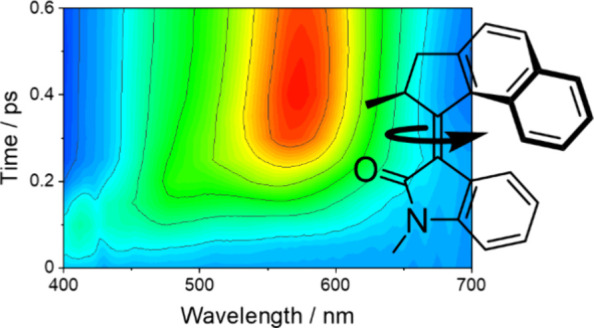

## Introduction

Molecular motors of the type first demonstrated by Feringa and co-workers use the absorption of light and thermal energy to drive successive steps in a unidirectional 360° internal rotational cycle.^1^ This class of overcrowded alkene molecular motor operates via a four-step cycle, as shown in Scheme 1 for an example *N*-methyl oxindole motor **1**, whereby the top half of the motor (rotor) rotates unidirectionally about the alkene bond with respect to the bottom half (stator). The initial step of the rotational cycle is the absorption of light, inducing a reversible *E-Z* photoisomerization of the alkene bond to establish a photostationary state (PSS) of the initial stable (*P*,*P*)-*E*-**1** and metastable (*M*,*M*)-*Z*-**1** diastereomers; the short-lived (*M*,*M*)-*Z*-**1** diastereomer undergoes a thermal relaxation process and inverts its helical chirality to form the stable diastereomer (*P*,*P*)-*Z*-**1** by a process called thermal helix inversion (THI). This two-step process is repeated once more to reform the initial diastereomer (*P*,*P*)-*E*-**1** (via (*M*,*M*)-*E*-**1**) to complete the unidirectional 360° rotation after a further photoisomerization and subsequent THI.

**Scheme 1:**
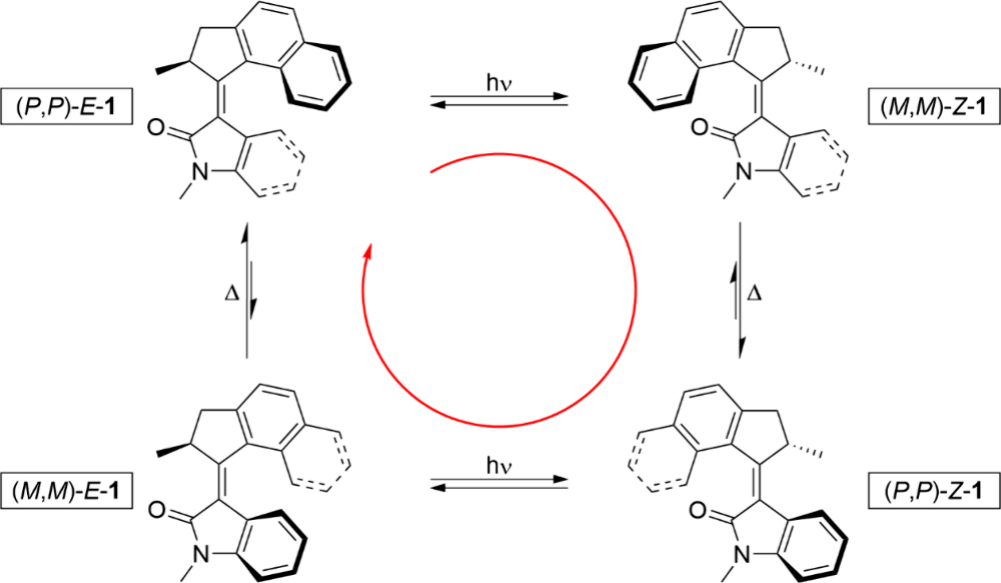
Four-Step Rotary Cycle of an *N*-Methyl Oxindole Motor **1**, whereby the Rotor Rotates Unidirectionally with Respect to the Stator about the Central Alkene Bond *via* Alternating Photoisomerization and THI Steps

Numerous reported overcrowded alkene motors function by irradiation with ultraviolet (UV) light.^1–8^ This UV activation poses a potential obstacle to their future practical applications because UV light is detrimental to biological and soft materials, which are potentially fertile fields in which to apply molecular motors. To avoid the use of harmful UV light, efforts have been made to shift the actinic wavelength into the visible region and even beyond into the near-infrared (near-IR). This objective has been achieved through various means; notable strategies include greater conjugation of the stator^9^ or rotor,^10^ metal coordination,^11^ donor–acceptor functionality,^12^ and the use of appended sensitizers.^13^ In one such example of motor redesign, Roke et al. reported a new class of overcrowded alkene motor based on an *N*-methyl oxindole stator.^14^ The oxindole-based motor design was later improved by Pooler et al., implementing a biomimetic photoswitch motif to enhance the charge-transfer character of the S_1_ excited state and boost the isomerization quantum yield (Φ_iso_).^15^ The motors they designed are shown in Figure 1 (**1** – **5**). These *N*-methyl oxindole overcrowded alkene motors operate at excitation wavelengths lying within the near-UV and visible regions. Visible-light irradiation yields less favorable PSS ratios compared to irradiation with near UV-light because of the comparatively greater absorption from the (*M*,*M*)-*Z* metastable diastereomer at longer wavelengths. Nevertheless, *N*-methyl oxindole motors have two main advantages over previous designs: they can be fuelled by less harmful visible light, and they have a relatively facile total synthesis.

**Figure 1:**
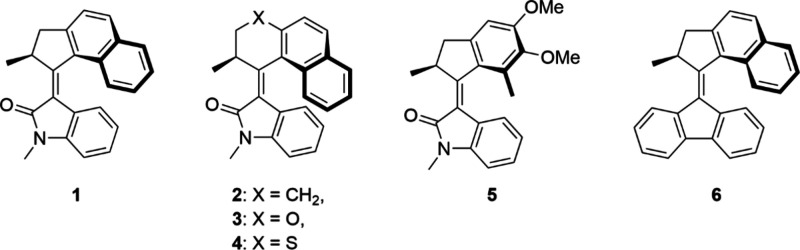
Structures of *N*-methyl oxindole motors **1**–**5** and the archetypal second-generation fluorene motor **6.**

While extensive research has sought to understand and improve the speed of the rate-limiting THI,^2,4,16^ comparatively less is understood about the excited-state dynamics of overcrowded alkene motors. Developing this understanding requires both ultrafast spectroscopic techniques that can observe molecular dynamics on the femto- to picosecond time scale, and high-level computational analysis to elucidate the potential energy surface (PES) landscapes that control the photoexcited motor dynamics.^15,17–21^ The photoinduced dynamics of the most thoroughly investigated fluorene overcrowded alkene motor **6** (Figure 1) have been analyzed using ultrafast fluorescence up-conversion spectroscopy, transient absorption (TA) spectroscopy, time-resolved infrared (TRIR) spectroscopy, and femtosecond stimulated Raman spectroscopy (FSRS), and much is known about the function of this particular motor in its electronically excited state.^22–25^ The deduced photodynamics are summarized schematically in Figure 2, and are shown in the current work also to be relevant to the *N*-methyl oxindole motors **1** – **5**. Fluorescence up-conversion spectroscopy experiments revealed that within the first 0.1 ps after excitation,^22^ the fluorene-containing motor moves away from the initially bright Franck–Condon (FC) region of the adiabatic S_1_ PES, toward the S_1_ minimum, which is referred to as a dark state because of the weak transition dipole moment for radiative decay to the electronic ground state S_0_.Two important nuclear coordinates were noted for the decay of the FC state to the dark-state minimum: torsion of the central alkene bond and pyramidalization of the olefinic carbon of the fluorene stator. The fluorescence emission signals exhibited biexponential ultrafast decay, with subpicosecond (0.29 ps) and picosecond (1.5 ps) time constants at a 556 nm emission wavelength. The early time (<1 ps) kinetic traces displayed damped oscillations, characteristic of dephasing of coherent vibrational motion for molecules in the electronically excited state.^22^ The sub-ps time constant was assigned to motion away from the FC region, and the 1.5 ps time constant to the internal conversion (IC) from the dark state to the electronic ground state S_0_. TA spectroscopy confirmed the contribution of the decay of the dark state by the elucidation of a 1.5 ± 0.3 ps time constant. The decay of the FC state was shown to be only weakly dependent on solvent polarity and viscosity, whereas the decay of the dark state was independent of solvent polarity, but highly viscosity dependent.

**Figure 2:**
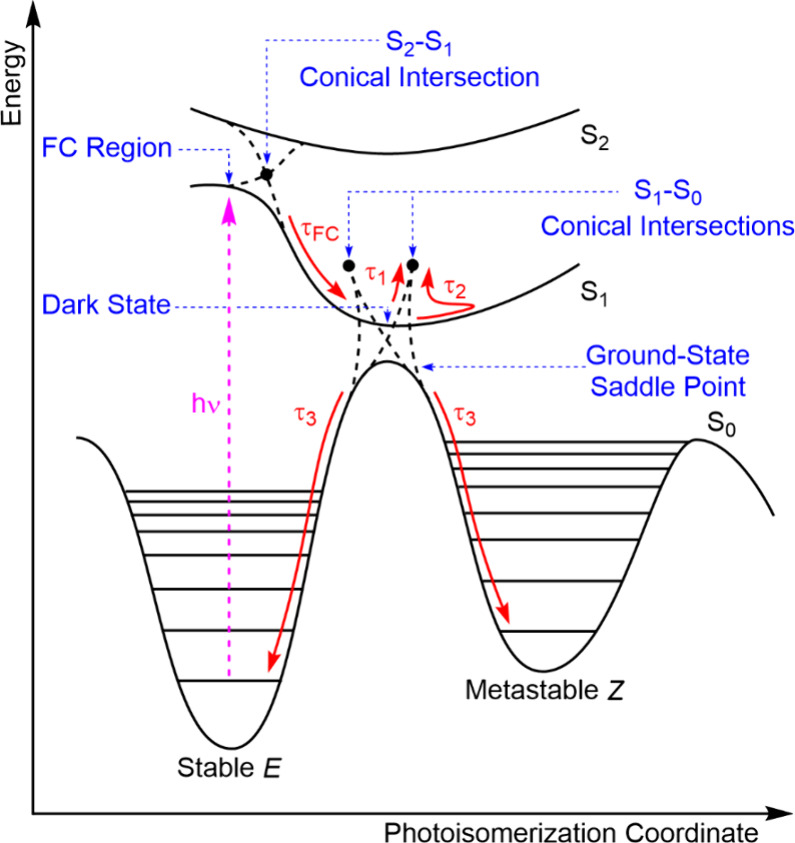
Proposed photochemical mechanism for the first step of motor operation, based on calculations reported by Pooler et al.^15^ and spectroscopic studies discussed in the main text. The bright FC region is reached by the absorption of light and is a region of the adiabatic S_1_ PES with high oscillator strength for radiative decay. The region closer to the minimum on the adiabatic S_1_ PES has low oscillator strength for radiative decay and is described as a dark state. The saddle point on the ground-state PES for the *E*–*Z* isomerization reaction is also shown. The red arrows show the dynamics on the excited- and ground-state PESs, and their associated time constants: the ultrafast relaxation from the FC region of the S_1_ state (τ_FC_), the direct (τ_1_) and indirect (τ_2_) decay of the dark state to the electronic ground state and vibrational cooling on the S_0_ PES (τ_3_). Conical intersections linking S_1_ and S_0_ are denoted by solid circles and are found at energies above the S_1_ minimum.

TRIR spectroscopy revealed added complexity to the excited-state dynamics by uncovering a rapidly decaying, wide and featureless excited-state absorption (ESA) band extending over a 1800 cm^–1^ range in the mid-IR spectral window (1200–3000 cm^–1^).^23^ This broad ESA was attributed to a low-energy electronic transition from the S_1_ state to the close-lying S_2_ electronically excited state, enhanced by vibronic coupling. The ESA band decayed biexponentially; the shorter of the reported time constants (1.53 ± 0.03 ps) was consistent with the dark-state lifetime reported from the TA and time-resolved fluorescence measurements.^22^ The longer time constant for the biexponential ESA decay kinetics was reported to be 12 ± 1 ps, and was attributed to cooling of the vibrationally hot metastable diastereomer photoproduct and the initially photoexcited stable diastereomer in the S_0_ ground state.

Alongside these spectroscopic findings, high-level computational studies at the OM2/MRCI level of theory and trajectory surface hopping (TSH) calculations have helped to clarify the excited-state behavior of motor **6**.^20^ Optimized S_1_ PES calculations showed the presence of two S_1_/S_0_ conical intersections (CIs) and a global S_1_ minimum accessible from both starting diastereomers.^15,18,21^ Internal conversion through one CI dominated the computed trajectories, but the decay kinetics were biexponential because of direct and indirect motions toward the CI seam. This explanation was also invoked for a motor that is similar in structure to motor **1**, again with the CI higher in energy than the S_1 min_.^26^ Calculations of the time-dependence of the fluorescence emission were in good agreement with fluorescence up-conversion experiments,^22^ revealing that the oscillator strength for fluorescence was quenched on an ultrafast time scale. The calculations confirmed that the dark state is not a different electronic state, but an area of the adiabatic S_1_ PES of different diabatic character with low oscillator strength for radiative decay, in contrast to prior time-dependent density functional theory (TD-DFT) calculations.^23^

The lifetime of the dark state and the isomerization quantum yield (Φ_iso_) are sensitive to changes in the electronic character of several overcrowded alkene motor designs, as has been demonstrated using synthetic redesigns utilizing functional groups with varying electron deficiency.^7,12,27^ Electron-withdrawing moieties promote a greater degree of pyramidalization of the olefinic carbon of the fluorene, whereas electron donating groups inhibit this effect. The greater degree of pyramidalization alters the nuclear geometry of the S_1_ minimum structure and the S_1_/S_0_ CI to enhance the proportion of photoproduct diastereomer formed, and thus increases isomerization quantum yield. Corroborating computational studies highlighted the importance of the pyramidalization coordinate in the excited-state dynamics of overcrowded alkene motors,^17,18,20,21^ similar to simpler alkenes such as ethylene and stilbene.^28–31^ The Φ_iso_ values for non-*N*-methylated analogues of *N*-methyl oxindole motor **1** have also been shown to increase using similar substitution methodology at the 5′ and at the 5 positions.^32^ Enhancing the charge-transfer character of the S_1_ excited state increases the isomerization quantum yield, and remarkably for a first-generation motor, Φ_iso_ ≈ 1.0 has been achieved in deuterated cyclohexane.^12,15^

The recently reported *N*-methyl oxindole motor **5** was subjected to TA spectroscopy measurements for solutions in methanol and *n*-hexane, and to a thorough computational study.^15^ The results show that **5** exhibits similar excited-state behavior to the analogous fluorene overcrowded alkene motor **6.** A persistent absorption feature was attributed to the formation of the (*M*,*M*)-*Z* metastable diastereomer of **5**, which is shifted to longer wavelength when compared to the UV–vis spectrum of the (*P*,*P*)-*E* diastereomer – characteristic of the metastable diastereomers of overcrowded alkene motors.^9,14,33^ A ground-state bleach (GSB) band recovered to within 8% of its original intensity, adding further support to the reported Φ_iso_ = 8% value measured using steady-state methods. The decay of the ESA bands exhibited oscillations attributed to a vibrational mode in the S_1_ excited state that promotes motion toward the S_1_ minimum dark state. This prior study provides a detailed explanation of the excited-state dynamics of this motor in methanol and *n*-hexane; however, further TA and TRIR spectroscopy studies of a wider range of *N*-methyl oxindole motor designs are needed to provide a fuller understanding of the excited-state dynamics of this class of overcrowded alkene molecular motor, and to explore how the motor dynamics are affected by their chemical environment.

Herein, motors **1** – **5** are submitted to both TA and TRIR experiments to elucidate the excited-state dynamics of *N*-methyl oxindole overcrowded alkene motors and their dependence on motor structure. Measurements are compared for solutions in three solvents chosen for their different bulk properties to explore further the dependence of the rotor dynamics on solvent polarity and viscosity. The outcomes are interpreted by drawing on insights from the prior spectroscopic and computational studies.

## Methods

### Synthesis of Oxindole Motors 1–5

The oxindole motors **1** – **5** were synthesized following established procedures from the Feringa group (Scheme S1).^2,8,14,15,34–36^ All intermediates and final motor compounds were characterized using ^1^H and ^13^C NMR, mass spectrometry and melting points (full characterization data are reported in Sections S2 – S3 in the Supporting Information).

### TA and TRIR Experimental

The TA and TRIR experimental setups used for the experimental measurements have been described thoroughly elsewhere and are summarized here.^37,38^ The ultrafast laser system was comprised of a Coherent Vitara-S oscillator and Coherent Legend Elite HE+ regenerative amplifier which produced 800 nm wavelength, 35 fs duration pulses at a frequency of 1 kHz and an output power of 4.5–5.0 W. TA and TRIR spectra were collected following UV or visible excitation of samples continuously circulating through a stainless-steel Harrick cell fitted with CaF_2_ windows sealed by Kalrez o-rings, and with window separation determined by Teflon spacers. Sample circulation used a peristaltic pump (Cole Palmer, Masterflex) and PTFE tubing. Further details including excitation wavelengths, sample concentrations, and window spacers, are provided in the Supporting Information (Table S1).

The optical path used in the TA and TRIR experiments is depicted in Figure S1. The 800 nm pulses were converted to UV and visible pump pulses and IR probe pulses for TRIR experiments using two Coherent OPerA Solo optical parametric amplifiers (OPAs). The broadband white-light continuum (WLC) used as a probe in the TA spectroscopy experiments was produced by focusing a small portion of the 800 nm pulses into a CaF_2_ window (thickness = 2 mm, f = 200 mm). The instrument response function of this experiment was previously determined to be 120 fs for TA measurements,^37^ and is similar for the TRIR experiments. The pump and WLC probe pulses were focused and spatially overlapped at the sample using an f = 200 mm CaF_2_ lens and an f = 75 mm concave aluminum mirror, respectively, to ensure a pump-beam focal spot size (previously measured to be 250 μm for the fwhm of the Gaussian beam profile) that was larger than the WLC probe spot size (50 μm fwhm). Pump pulse fluences were attenuated to below 1 μJ pulse^–1^ to avoid multiphoton artifacts in the experimental measurements. The delay between the pump and probe pulses was controlled by an optical delay stage (Thorlabs DDS220/M) fitted with an aluminum retroreflector and giving a maximum delay of 1.3 ns. This delay stage was positioned in the pump laser beam path. A synchronized chopper wheel (Thorlabs MC2000) blocked every other pump laser pulse to allow accumulation of both pump-on and pump-off absorption spectra using alternate laser shots. The linearly polarized pump and probe pulses were set with their polarizations at a magic angle of 54.7°.

After the probe pulses passed through the sample, they were dispersed in a spectrometer fitted with an array detector to accumulate transient spectra across the bandwidth of the probe pulses. For TA spectroscopy, the detector was a 1024-element photodiode array (Entwicklungsbüro Stresing) fitted to a spectrograph (Andor, Shamrock 163). For TRIR measurements, the detector was instead a 128-element, liquid-N_2_ cooled mercury cadmium telluride (MCT) linear array (InfraRed Associates Inc., MCT-10–128) mounted in a Horiba Scientific spectrometer (iHR320) and fitted with read-out electronics (Infrared Systems Development Corp., FPAS-0144) to record spectra for each probe laser pulse. A portion of the IR pump beam was separated using a beam splitter located before the sample and directed to a matched IR spectrometer and 128-element array detector for spectral referencing to reduce noise arising from shot-to-shot intensity fluctuations. The enclosed IR beam paths and spectrometers were purged with dry nitrogen gas to reduce the effects of atmospheric water absorption. All the components of the TA and TRIR experiments including the delay stage and data transfer from the spectrometers were controlled by a custom-written LabView program (TAPIR).

### Computational Methods

All the computational calculations for oxindole motors **1** – **5** were carried out using density functional theory (DFT). The lowest energy conformers of each compound were identified using the Spartan 20 V1.0.0 software package and subsequently optimized in Gaussian 09W software using the ωB97XD functional and the 6–31+G­(d,p) basis set for the oxindole motors **1** – **5**.^39^ With the optimized structures in-hand, each structure was submitted to time-dependent DFT calculations to calculate the vertical transitions. Using the results from the TD-DFT calculations, the two lowest energy transitions were used to calculate the natural transition orbitals (NTOs), to show the orbital character for each transition.

The ωB97XD functional was chosen because agreement between the experimental and calculated UV–vis and FTIR spectra obtained using the B3LYP and CAM-B3LYP functionals was poorer. Previously, B3LYP has been employed to calculate the ground state optimized geometries of fluorene molecular motors.^5,20,23^ However, the B3LYP functional does not include a dispersion correction term needed for overcrowded alkene motor molecules which possess a large degree of steric repulsion in the fjord region. The ωB97XD functional also includes a long-range correction term, which is important for the oxindole motors which possess a degree of charge-transfer character in the excited state. The 6–31+G­(d,p) basis set was selected because it is both relatively computationally inexpensive and gave calculated spectra for the oxindole motors that agreed satisfactorily with the experimental spectra.

## Results and Discussion

### Steady-State Absorption Spectroscopy

Motors **1** – **5** were submitted to UV–vis and FTIR spectroscopic analysis, using the same sample concentrations and pathlengths as the respective time-resolved TA and TRIR experiments (see Table S1 of the SI). The UV–vis absorption spectra and associated molecular orbitals (MOs) and natural transition orbitals (NTOs) obtained from TD-DFT calculations are shown in Figures S2 – S5.

The absorption band maxima for **1** are at 366 nm, 376 and 374 nm in cyclohexane, DMSO and methanol respectively (Figure 3). Motors **1**, **2** and **5** display two absorption bands in their UV–vis absorption spectra. The first of these absorption bands for each of these motors appears as a sharp peak within the range 340–370 nm, whereas the second is a broader peak spanning 370–475 nm, and thus extending into the visible region. TD-DFT calculations conducted for motor **1** at the ωB97XD/6–31+G­(d,p) level of theory (Figure 3A) show that the two absorption bands are caused by photoexcitation to the S_1_ and S_2_ excited states. The calculated electronic transitions are centered at 360 and 310 nm, which are 50 nm shifted to shorter wavelength than observed in the UV–vis spectra. Some of this shift can be attributed to comparison of experimental spectra obtained for solutions with calculations for an isolated molecule. Apparent mismatches in the relative intensities of the two bands when comparing experimental spectra to computed oscillator strengths arise from the greater width of the S_0_ → S_1_ band and the partial overlap of these two features in the measured absorption spectra. Using the TD-DFT calculations, the molecular orbitals and NTOs for these transitions were calculated and are shown in Figure 3B and Figure 3C for **1**, respectively. Both the S_0_ → S_1_ (HOMO → LUMO) and S_0_ → S_2_ (HOMO–1 → LUMO) transitions are characterized as ππ*.

**Figure 3:**
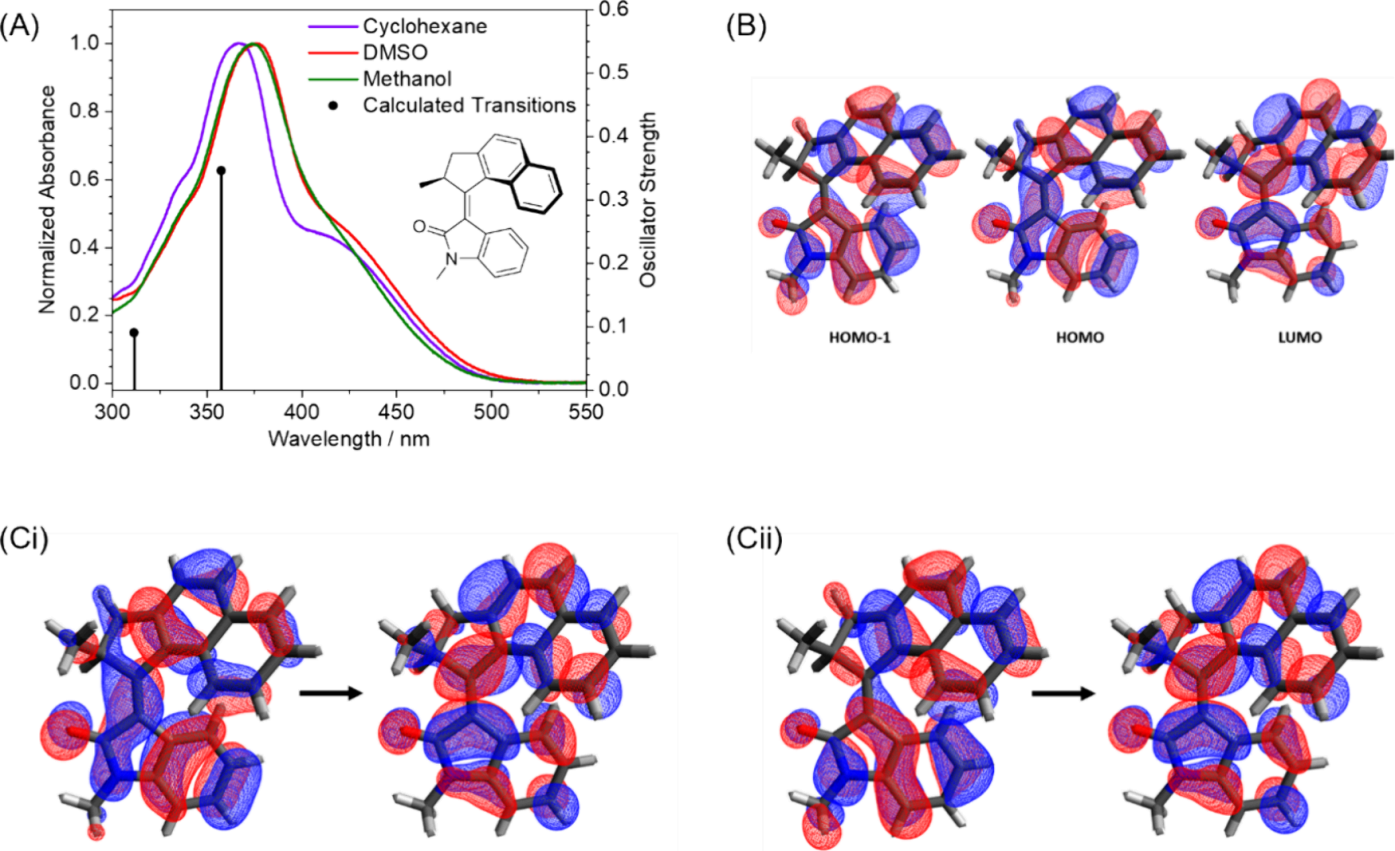
(A) Normalized steady-state UV–vis absorption spectra of motor **1** in three solvents: cyclohexane (violet), DMSO (red) and methanol (green). The vertical black lines show electronic transitions calculated using TD-DFT at the ωB97XD/6–31+G­(d,p) level of theory in the gas phase. (B) Calculated HOMO–1, HOMO and LUMO molecular orbitals. Calculated NTOs for the (Ci) first and (Cii) second electronic transitions for motor **1**.

Limited solvatochromism (≤10 nm) is observed for all the motors. Notably, in the case of all five motors studied, the absorption spectra are shifted to longer wavelength in the polar DMSO and methanol solvents by stabilization of the ππ* states. The UV–vis spectra for the heterocyclic motors **3** and **4** are distinct as there appears to be only one absorption band. This finding is supported by the calculated oscillator strengths for the second electronic transition, found to be 0.037 and 0.007 for **3** and **4**, respectively.

The recorded FTIR spectra and calculated vibrational modes are presented in the SI (Figures S6 – S10). These spectra were obtained in deuterated solvents because the nondeuterated solvents exhibit vibrational bands in the region of interest (1400–1800 cm^–1^). This range contains the C = O stretch observed from 1650–1725 cm^–1^ and aromatic modes from 1440–1675 cm^–1^_._ All the vibrational modes found in these regions show considerable C = C vibration of the central alkene bond. The C = O bond stretch is present at lower wavenumber in methanol when compared to cyclohexane or DMSO solutions because of the effects of hydrogen bonding.

### Transient Absorption and Time-Resolved Infrared Absorption Spectroscopy

Although our experimental investigations have examined the ultrafast photochemical dynamics of molecular motors **1** – **5** in three solvents, the time-resolved spectroscopy results presented here focus on the chosen example of motor **1**. Comparisons are then drawn between the molecular structure- and solvent-dependent excited-state behaviors of the five studied motors. The time-resolved TA and TRIR spectra for all the motors **1** – **5** are reported in the SI (Figures S11–25) and the corresponding fitted kinetic data are shown in Figures S28 – S42. The fitting procedures and examples of the spectral decomposition methodology used are illustrated in Scheme S2 and Figures S26 – S27.

Figure 4B shows TA spectra recorded for a 1.4 mM solution of **1** in DMSO, with spectra plotted for time delays 0.4–100 ps after photoexcitation using 375 nm light. Four main features are observed: a GSB band, a partially structured broad ESA band, a stimulated emission (SE) band and a photoproduct absorption. The negative change in absorbance detected from 350–425 nm is attributed to a GSB feature, and is partially obscured by residual pump light scatter centered at 375 nm; consequently, the fitting of the GSB band is less reliable than the fitting procedure for the ESA bands and therefore is not used.

**Figure 4:**
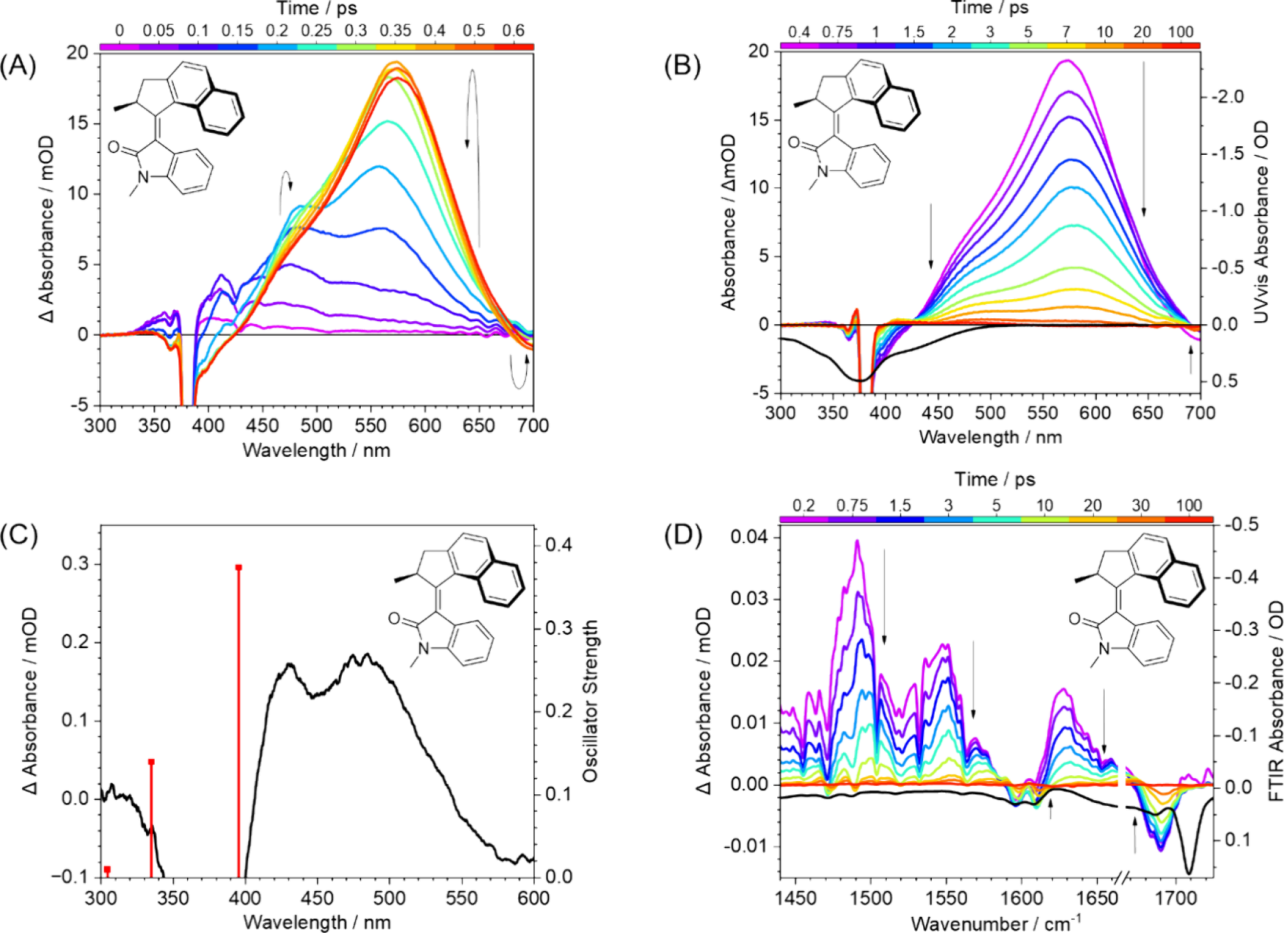
Temporal evolution of the transient absorption spectra of motor **1** in DMSO: (A) for time delays of 0–0.6 ps, (B) for time delays of 0.4–100 ps, (C) for 900 ps time delay, showing the (*M*,*M*)-*Z*-**1** photoproduct of the isomerization reaction and calculated TD-DFT electronic transitions at the ωB97XD/631+G­(d,p) level of theory. This spectrum has been smoothed using adjacent averaging in Origin2022b software. (D) TRIR spectra for motor **1** in DMSOd_6_ for time delays 0.2–100 ps. In panels (B) and (D), black curves are inverted steady-state UV–vis and FTIR spectra, respectively, to show regions of parent absorption. In panels (A–C), strong negative-going signals at 375 nm are scatter of the pump laser radiation. The probe WLC does not extend below 350 nm. The arrows illustrate how the time-resolved spectral features evolve over time.

The positive change in absorbance arises from a double-humped ESA feature comprised of two apparently overlapping bands observed in the range 425–700 nm with maxima at 480 and 575 nm. The shorter-wavelength ESA band overlaps and masks part of the GSB feature, which should extend to 520 nm according to the known UV–vis absorption spectrum, as shown by the inverted black line in Figure 4B. The two ESA bands are attributed to transitions to higher lying singlet states (S_1_ → S_n_, S_n+1_, ···) from a species formed in the electronically excited S_1_ state within 0.4 ps after photoexcitation. More examples of early time spectra (<0.6 ps) are shown in Figure 4A. The ESA band centered at 480 nm reaches maximum intensity at 0.2 ps and begins to decay; concomitantly, the band centered at 575 nm continues to increase in intensity until 0.4 ps, thereafter it also decays. An ill-defined isosbestic point is observed at 520 nm, indicating that there is a change in structure of the excited-state species at these early time delays. The isosbestic point is blurred by rapid vibrational cooling and decay of the excited-state population.

After 0.4 ps, both ESA bands begin to decay to the baseline, revealing a persistent photoproduct absorption (Figure 4C) which is observed until 900 ps after photoexcitation and is stable on this time scale. The double-humped photoproduct absorption band is shifted to longer wavelengths than the UV–vis spectrum of the parent diastereomer (*P*,*P*)-*E-* **1**, with peak maxima at 430 and 485 nm. This photoproduct is attributed to the *(M*,*M*)-*Z*-**1** metastable diastereomer, shown in Figure 1. Similar assignments have been reported for previous TA experiments on analogous overcrowded alkene motors.^5,7,12,15^ Our assignment is supported by TD-DFT calculations (Figure 4C) which show that the S_0_ → S_1_ and S_0_ → S_2_ transitions arise at longer wavelengths than the calculated electronic transitions shown in Figure 3 for the (*P*,*P*)-*E*-**1** diastereomer. No decay of the (*M*,*M*)-*Z*-**1** diastereomer absorption is observed in the TA spectra because the half-life of the metastable diastereomer is 50 h at 20 °C.^14^

The negative-going change in absorbance at wavelengths >680 nm is ascribed to a SE band that appears to decay rapidly and shift to longer wavelength, although our spectral window is limited. Short-lived SE bands have been observed for related overcrowded alkene motors.^12,15^ Only the 680–700 nm wavelength portion of the SE band is observed here, which, together with its rapid decay and wavelength shift, prevents a reliable quantitative analysis. However, the SE feature cannot be observed at times longer than 0.75 ps, which suggests that the transient species responsible rapidly relaxes to a geometry or an electronic state that is either nonemissive, or emits at wavelengths >700 nm.

Figure 4D shows TRIR spectra for a solution of **1** in DMSO-*d*_6_ for delays from 0.2–100 ps after photoexcitation at 375 nm. The FTIR spectrum is shown as a black line with an inverted absorbance axis to highlight where GSB bands are expected to appear in the TRIR spectra. Three main types of feature are seen: three broad ESA bands have sharper GSB bands overlaid, and hot groundstate (HGS) absorptions are observed as positive absorption bands at slightly lower wavenumber than the GSB bands. The band centered at 1709 cm^–1^ in the FTIR spectrum is not observed as a GSB in the TRIR spectra, most likely because of overlapping ESA bands. Sharp dips, for example at 1505 cm^–1^ and 1535 cm^–1^ are caused by gas-phase water absorption lines in the mid-IR beam path. Incomplete recovery of the GSB bands provides additional evidence for photoproduct formation, and the degree of the carbonyl stretch GSB recovery is used to estimate the upper limit for Φ_iso_ (see below). The broad ESA bands have maxima at 1490 cm^–1^, 1550 cm^–1^ and 1625 cm^–1^. These vibrational ESA bands shift to higher wavenumber as they decay, indicating that the excited-state population is both dissipating its excess vibrational energy to the surrounding solvent, and that solvent-reorganization is occurring to solvate excited-state molecules with some charge-transfer (CT) character. Band shifts on this time scale are not resolved in the ESA features seen by TA spectroscopy.

Figure 5 shows the excited-state decay kinetic traces obtained by decomposition of the TA and TRIR spectra and wavelength or wavenumber integration of the time-dependent intensities for the constituent bands. For the decomposition of TA spectra, the time-dependent intensity of the GSB band in the TA spectra was fitted in KOALA software using the UV–vis absorption spectrum as a basis function, and the double-humped ESA bands were simultaneously fitted using Gaussian functions centered at 480 and 575 nm, respectively.^40^ The resulting kinetic traces for growth (much of which is IRF limited) and decay of ESA intensity were fitted in Origin software using a Gaussian-convoluted biexponential decay function. The Gaussian convolution accounts for the instrument response for the TA measurements, which is determined to be 120 fs from the fitting procedure. The spectral overlap of the GSB, ESA and photoproduct features (350–550 nm) complicates the extraction of time-dependent intensities for the lower-wavelength component of the broad ESA feature, but the kinetic traces show only modest differences for the decays of the two ESA bands. The analysis reveals two time constants τ_1_ = 1.60 ± 0.10 ps and τ_2_ = 5.76 ± 0.52 ps (Figure 5A), where the stated error is obtained from the fitting procedure. The kinetic data at time delays below 0.55 ps were removed from the fitting procedure for the ESA band centered at 480 nm, because the decay in this time range in nonexponential. Similar excited-state behavior was previously observed for motor **5** and was attributed to nonexponential relaxation caused by ultrafast dynamics in the S_1_ adiabatic potential.^15^

**Figure 5:**
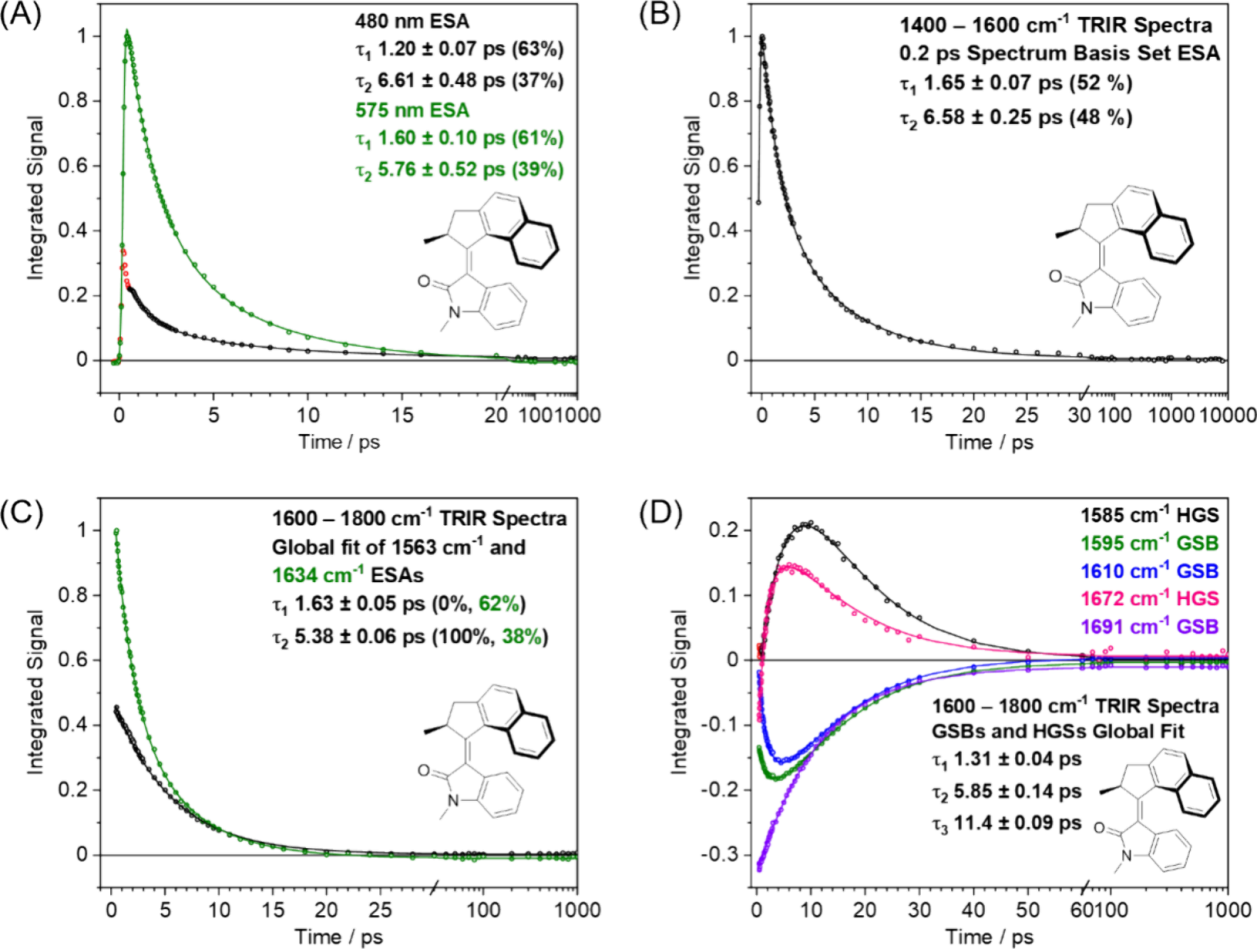
(A) Excited-state kinetics deduced from the TA spectra of **1** acquired in DMSO, highlighting biexponential decays fitted to integrated band intensities obtained by spectral decomposition using the two Gaussian functions centered at the wavelengths stated in the inset. The <0.55 ps 480 nm band intensity data (red circles) are omitted from the fitting procedure (see main text). (B, C) Biexponential excited-state kinetics of **1** derived from TRIR spectra in DMSO-*d*_6_ collected in the 1400–1600 and 1600–1800 cm^–1^ regions, respectively. These kinetics were acquired using the integrated band intensities derived from spectral fitting with (B) the 0.2 ps TRIR spectrum as a basis function; and (C) Gaussian functions centered at the wavenumbers stated in the inset. (D) Kinetics of the GSB and HGS TRIR bands obtained from the 1600–1800 cm^–1^ region, fitted to integrated band intensities for Gaussian basis functions centered at the wavenumbers stated in the inset.

For the TRIR spectra obtained in the 1400–1600 cm^–1^ region, the changing intensities of the ESA bands of motor **1** were fitted using the 0.2 ps TRIR spectrum as a basis function, with the GSB bands removed because of their different time-dependence and instead fitted using Gaussian functions. This analysis reveals a biexponential decay of the ESA bands for motor **1** in DMSO-*d*_6_ with time constants τ_1_ = 1.65 ± 0.07 ps and τ_2_ = 6.58 ± 0.25 ps (Figure 5B). Global fitting of the GSB and HGS absorptions reveals three time constants: τ_1_ = 1.31 ± 0.04 ps, τ_2_ = 5.85 ± 0.14 ps and τ_3_ = 11.4 ± 0.09 ps (Figure 5D). The ESA bands observed in the 1600–1800 cm^–1^ region were fitted using Gaussian functions to reveal biexponential decay kinetics with time constants of τ_1_ = 1.63 ± 0.05 ps and τ_2_ = 5.38 ± 0.06 ps (Figure 5C). Our mechanistic interpretations of the time constants are shown in Figure 2, and their derived values are in good agreement between the TA and TRIR spectra, suggesting that both experiments are probing the same excited-state species. The longer time component τ_3_ uncovered by the TRIR experiment is ascribed to vibrational cooling of the hot ground-state photoproduct (*M*,*M*)-*Z*-**1** and unreacted starting diastereomer (*P*,*P*)-*E*-**1.**

The TA and TRIR data for solutions of motor **1** in DMSO show that the excited-state dynamics of this oxindole motor are similar to those for the fluorene stator motor **6**, therefore comparisons between the two motors can be made. Based on previous spectroscopic studies and high-level computational calculations for motor **6**,^20,22^ the ultrafast nonexponential decay kinetics (τ_FC_) observed here on time scales comparable to our IRF can be attributed to structural evolution in the adiabatic S_1_ state from the bright FC geometry to the minimum-energy geometry in the dark-state region, as shown in Figure 2. This interpretation is supported by our own computational interpolation study of the (*P*,*P*)-*E*-**1** → (*M*,*M*)-*Z*-**1** isomerization reaction. The structures of the ground-state (*P*,*P*)-*E* and (*M*,*M*)-*Z* diastereomers of motors **1** – **5** were optimized at the ωB97XD/6–31+G­(d,p) level of theory, and interpolation in internal coordinates between these two structures was then used to model the geometric evolution induced by the photochemical isomerization reaction. All interpolated structures were subjected to TD-DFT calculations at the ωB97XD/6–31+G­(d,p) level of theory to calculate the vertical transition energies to the S_1_ and S_2_ singlet excited states. The results of the interpolation study for motor **1** are shown in Figure 6, with results from the corresponding interpolation studies of the other motors **2** – **5** shown in the SI (Figures S47 – S50).

**Figure 6:**
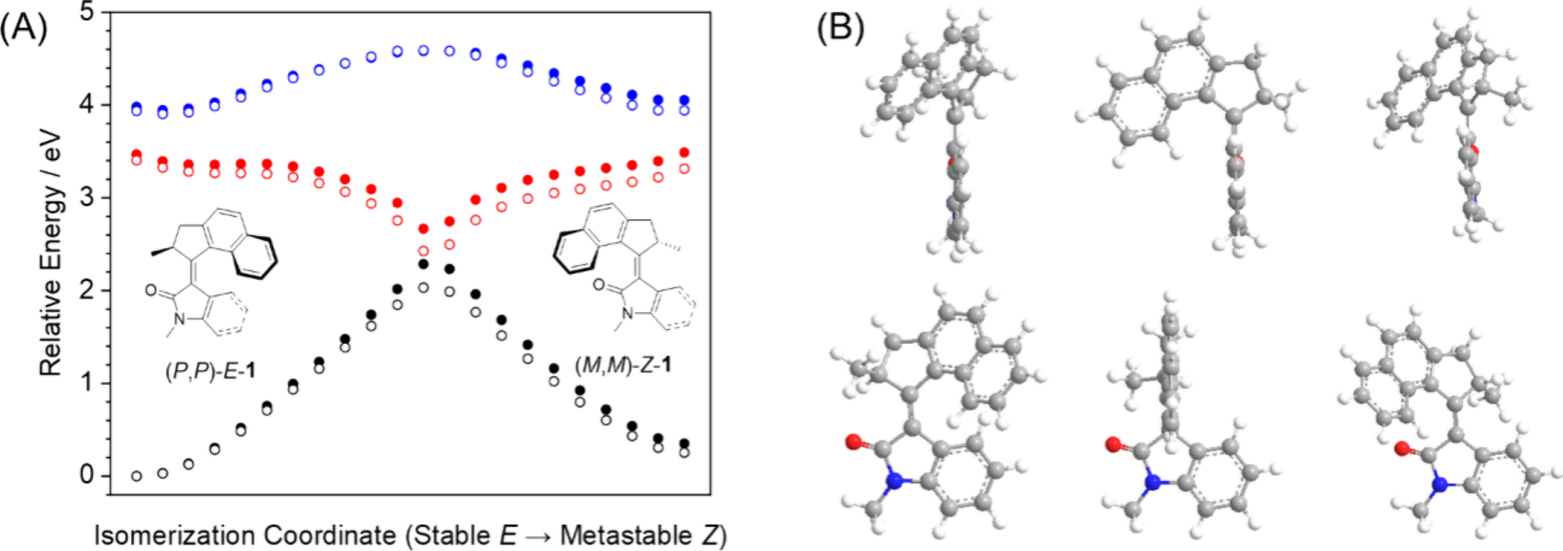
(A) Relative energies calculated at the ωB97XD/6–31+G­(d,p) level of theory for the interpolated structures of motor **1** in the S_0_ (black), S_1_ (red), and S_2_ (blue) electronic states. Calculations were performed for the gas phase (solid circles) and a DMSO solution using a PCM model (hollow circles). (B) Structures of the optimized (*P*,*P*)*-E*-**1** diastereomer, the interpolated isomerization saddle point on S_0_ and the optimized (*M*,*M*)*-Z*-**1** diastereomer. In each case, two views of the same structure are shown.

A previous TRIR and TD-DFT study of **6** showed the presence of a low-lying S_2_ excited state, a small S_1_–S_2_ energy gap (corresponding to absorption of light in the mid-IR) and a change of electronic character as the excited-state population moves from the FC state to the dark state.^23^ The TRIR ESA bands we observe for the oxindole motors **1** – **5** resemble the wide and featureless ESA bands attributed to an S_1_ → S_2_ electronic transition for fluorene motor **6**. However, we prefer a different interpretation of the ESA bands in our TRIR spectra, in part because our calculations (Figure 6A) show that the energy separation of the S_1_ and S_2_ PESs as a function of the C = C rotational coordinate is too great for an ESA contribution in the mid-IR region. To test the effects of solvation by DMSO, *E* and *Z* isomer structures were optimized using a DMSO IEFPCM, and the calculated energies of intermediate structures also used this IEFPCM. The general trends for these calculations incorporating DMSO solvation are the same as for our gas phase calculations, although the saddle point energy on the S_0_ ground state and the S_1_ minimum energy are slightly lowered, while the S_2_ state remains largely unaffected. The photon energy corresponding to our mid-IR probe window of 1400–1800 cm^–1^ is 0.17–0.23 eV, whereas the smallest S_1_ – S_2_ energy gap calculated in our interpolation study is 0.52 eV for the (*P*,*P*)-*E* diastereomer, and widens rapidly to >1 eV as the dark state region is approached. We therefore propose that the ESA bands in our TRIR spectra arise from vibrational transitions for a nonthermal distribution of vibrational quanta of the molecules in the dark-state region of S_1_. The full widths at half-maximum for the 1490 cm^–1^ and 1550 cm^–1^ TRIR ESA bands are 29 cm^–1^ and 41 cm^–1^ respectively. The expected line width due to lifetime broadening is ∼ 2 cm^–1^, calculated using the average dark state lifetime (<τ_ds_> = 3.1 ps, see below). Consequently, lifetime broadening is not considered the main cause of the width of the ESA bands. The broadening of the TRIR ESA bands might instead result from the dark state population possessing a wide range of structures, in accordance with previous observations of analogous motor molecules.^23,24,27^ The dark state is populated within <0.75 ps after photoexcitation, and will be vibrationally hot and nonthermal because this rate of growth exceeds the rate of vibrational cooling by coupling to the solvent bath. The widths of the ESA bands might therefore reflect a potential energy well near to the global minimum of the S_1_ adiabatic PES that is shallow along other distortion coordinates, allowing the excited molecules to adopt a range of geometries. This interpretation of vibrational energy redistribution and cooling in the region of the S_1_ minimum is also consistent with our observed shifts in the TRIR bands to higher wavenumber with increasing time delay, as is shown in Figure 5 D.

The nonexponential nature of the excited-state dynamics can be explained by the population evolving along the S_1_ adiabatic potential faster than the rate of vibrational relaxation, as was also observed for motor **5**.^15^ The dark state is formed by twisting the central alkene bond to form a structure with large twist angle, possessing low oscillator strength for radiative decay to S_0_. The dynamics of this process are observed in the TA spectra as the rapid growth then initial decay in the intensity of the 480 nm ESA band, and the slower rise of the 575 nm ESA band in the early time spectra (<0.6 ps). If, as seems likely, the FC state and dark state structures have different Franck–Condon factors for excitation to the higher lying S_n_ states, changes will be seen in the relative intensities of the two components of the broad ESA feature as the population relaxes from the FC region to the dark state. The SE band detected at longer wavelengths is observed in the early time spectra and is no longer detected at 0.75 ps after excitation. In this time, the excited-state population has left the bright FC region and has rapidly relaxed to the dark-state geometry.

The decay kinetics of the excited-state population of motor **1** are best described using three time constants τ_1_, τ_2_ and τ_3_. The dark state decays biexponentially with time constants τ_1_ and τ_2_. Nonsingle-exponential decay kinetics of the dark states of other overcrowded alkene motors have been noted previously.^5,7,12,24^ The observed decay kinetics can be interpreted in a few different ways. One proposition is that a proportion of the excited-state population finds a direct route to the conical intersection with the S_0_ state, while the remainder misses the CI seam and takes indirect pathways to access the CI. These molecules thus spend a greater amount of time in the S_1_ excited state. This hypothesis is supported by previously reported TSH calculations of two motors;^20,26^ of the two potentially accessible S_1_ /S_0_ CIs in the vicinity of the S_1_ minimum, only one was found to be operative, nevertheless biexponential decay kinetics through this CI were calculated. Multiple minima on the S_1_ adiabatic PES could also account for biphasic S_1_ decay, as reported from previous TA and FSRS studies of **6** that show different dark state lifetimes and structures if the motor is initially excited from the stable (*P*,*P*)-*E* or metastable (*M*,*M*)-Z diastereomers.^25^ If there are multiple minima on the S_1_ PES then the excited-state population can plausibly take distinct routes to the S_1_/S_0_ CI. Another possible explanation is that one of the time constants is caused by solvation of partial charge across the ethylenic bond as the C = C bond twists in the excited state. Solvent polarity has been shown to play a significant role in the lifetime of the dark state in motors believed to have charge transfer character.^12,41^

After the excited-state population has undergone IC to S_0_ where it forms the photoproduct (*M*,*M*)-*Z*-**1** or reforms the unreacted starting diastereomer (*P*,*P*)-*E*-**1**, the ground-state population has excess vibrational energy that is transferred to the surrounding solvent with a time constant τ_3_. This additional time constant contributes to the observed decay kinetics of the shorter wavelength component of the ESA band in our TA spectra, due to spectral overlap with the longer wavelength edge of the GSB feature. It is also observed in the decay of the broad ESA bands seen by TRIR spectroscopy because of spectral overlap with GSB and HGS bands. The isomerization quantum yield for this motor is deduced to be low because the photoproduct absorption is weak in the TA spectra, and the GSB almost entirely recovers. Hence, most of the excited-state population reforms the starting diastereomer (*P*,*P*)-*E*-**1**, in accordance with the previously reported low quantum yields determined using a potassium ferrioxalate actinometer and the methodology outlined by Stranius and Börjesson.^14,42^

### Solvent Dependence of the S_1_–State Relaxation Dynamics

Three solvents were selected to probe the solvent dependence of the excited-state dynamics in the five *N*-methyl oxindole motors **1** – **5**. Cyclohexane is a representative nonpolar and nonviscous solvent (ε = 2.02 at 20 °C and η = 0.894 mPa s at 25 °C), methanol is a polar and nonviscous solvent (ε = 33.0 at 20 °C and η = 0.544 mPa s at 25 °C), and DMSO is both polar and viscous (ε = 47.24 at 20 °C and η = 1.987 mPa s at 25 °C).^43^

Table 1 summarizes the results of our studies of the solvent-dependent photodynamics of motor **1**. In methanol, biexponential decay of the dark state is again observed (characterized by time constants τ_1_ and τ_2_). The time constants are comparable to those reported in DMSO; albeit both lifetimes are shorter, particularly the τ_1_ time component. Comparing the relative amplitudes of the two decay components, in methanol the shorter τ_1_ accounts for 89% of the decay kinetics compared to 61% for **1** in DMSO. In contrast, τ_1_ accounts for 100% of the decay kinetics in cyclohexane, where the decay of the dark state is monoexponential, with time constant τ_1_. The additional τ_2_ time constant may therefore be associated with polar solvents. This second lifetime component has the effect of increasing the average lifetime of the dark state as a greater proportion of the dark-state population decays via the excited-state dynamics associated with τ_2_.

**Table 1:** Summary of the Excited-State Dynamics of Motor **1** after Excitation with 375 nm Light in Three Solvents: Cyclohexane, DMSO, and Methanol^c^

		τ_1_/ps	τ_2_/ps	τ_3_/ps	<τ_ds_>/ps
cyclohexane	TA	1.16 ± 0.02			1.16 ± 0.02
	TRIR	1.11 ± 0.01^a^		12.7 ± 1.00^a^	1.11 ± 0.01^a^
		1.13 ± 0.01^b^		13.6 ± 0.24^b^	1.13 ± 0.01^b^
DMSO	TA	1.60 ± 0.10 (61%)	5.76 ± 0.52 (39%)		3.21 ± 0.42
	TRIR	1.65 ± 0.07^a^ (52%)	6.58 ± 0.25^a^ (48%)	11.4 ± 0.10^b^†	3.10 ± 0.12^a^
		1.63 ± 0.05^b^ (62%)	5.38 ± 0.06^b^ (38%)		3.43 ± 0.10^b^
methanol	TA	0.73 ± 0.02 (89%)	5.12 ± 0.54 (11%)		1.22 ± 0.09
	TRIR	0.78 ± 0.01^a^ (84%)	4.95 ± 0.26^a^ (16%)	9.06 ± 0.13^a^†	1.47 ± 0.06^a^

a ESA lifetime from TRIR spectra recorded in the 1400–1600 cm^–1^ spectral window.

b ESA lifetime from TRIR spectra recorded in the 1600–1800 cm^–1^ spectral window.

c The percentages express the proportion of the dark state that decays *via* directed motion (τ_1_) or indirect motion (τ_2_) toward the S_1_/S_0_ CI. These percentages are calculated from the ratio of the preexponential factors for τ_1_ and τ_2_ decay components, which quantify the bi-exponential lifetimes of the dark state. The average dark state lifetime (<τ_ds_>) is calculated from the weighted average of the τ_1_ and τ_2_ time constants. † Lifetime derived from global fitting of TRIR GSB and HGS absorptions.

As the molecules undergo relaxation in the S_1_ state, the ESA bands in the TRIR spectra shift 5–10 cm^–1^ to higher wavenumber in both methanol and DMSO, which is indicative of vibrational redistribution or cooling, whereas there is no notable shift in cyclohexane. Cyclohexane couples weakly to the vibrational modes of the dark-state population compared to methanol and DMSO. As a result, energy transfer from the vibrationally hot dark-state molecules to the solvent is slower in cyclohexane. Consequently, the dark-state population retains enough total internal energy, or retains excess vibrational energy in favorable vibrational mode(s), to access the S_1_/S_0_ CI directly. This weaker solvent coupling is also observed in the rate of vibrational cooling of the HGS molecules, where the τ_3_ time component is larger for **1** in cyclohexane compared to the polar solvents. Solvent-induced relaxation is expected to have a greater effect on the dark-state molecules, which are thought to have greater CT character and therefore interact more strongly with the polar solvent molecules. In these polar solvents, S_1_-state molecules can transfer more of their excess vibrational energy to the surrounding solvent shell during the subpicosecond structural evolution from the FC region to geometries corresponding to the dark state, causing a proportion of the population to miss the CI on the first pass (τ_1_), so requiring a more indirect route to the CI (τ_2_). These indirect dynamics correspond to intramolecular vibrational energy redistribution in the vicinity of the S_1_ minimum (Figure 2), and will also be affected by coupling to the solvent. This effect is compounded by the greater viscosity (and hence solvent friction) in DMSO, which has previously been shown to increase the excited-state lifetimes of other overcrowded alkene motors.^33^

### Structure-Dependence of the S_1_–State Relaxation Dynamics of Oxindole Motors

The observations summarized here for the excited-state dynamics of motor **1** largely hold true for the four other oxindole motors studied, for which the structures are shown in Figure 7. The time-resolved spectra for motors **1** – **5** (Figures S11 – S25), and the summarized excited-state decay kinetic data for all motors (Tables S2 – S6) are shown in the SI. Table 2 shows the corresponding average lifetimes of the dark state for each motor **1** – **5** in the three solvents. The excited-state decay kinetics are compared in Figure 8.

**Table 2:** Solvent-Dependence of the Average Dark-State Lifetimes (τ_DS_) of Motors **1**–**5** ^c^

		**1**	**2**	**3**	**4**	**5**
		τ_DS_/ps	τ_DS_/ps	τ_DS_/ps	τ_DS_/ps	τ_DS_/ps
cyclohexane	TA	1.16 ± 0.02	4.01 ± 0.07	1.13 ± 0.02	3.43 ± 0.04	0.48 ± 0.01
	TRIR	1.11 ± 0.01^a^	3.99 ± 0.12^a^	1.08 ± 0.03^a^	3.44 ± 0.12^a^	0.45 ± 0.02^b^
		1.13 ± 0.01^b^	4.08 ± 0.13^b^			
DMSO	TA	3.21 ± 0.42	10.8 ± 3.7	3.76 ± 0.26	13.1 ± 1.1	1.25 ± 0.13
	TRIR	3.10 ± 0.12^a^	10.9 ± 1.22^a^	4.74 ± 0.88^a^	15.2 ± 0.51^a^	1.79 ± 0.07
		3.43 ± 0.10^b^				1.27 ± 0.06
methanol	TA	1.22 ± 0.09	3.58 ± 0.54	0.89 ± 0.07	3.75 ± 0.33	0.51 ± 0.07
	TRIR	1.47 ± 0.06^a^	4.32 ± 0.92^a^	1.19 ± 0.16^a^	5.50 ± 0.78^a^	0.79 ± 0.03^a^

a ESA lifetime from TRIR spectra recorded in the 1400–1600 cm^–1^ spectral window.

b ESA lifetime from TRIR spectra recorded in the 1600–1800 cm^–1^ spectral window.

c The τ_1_ and τ_2_ time constants used to calculate the average dark-state lifetimes are extracted from the fitting of the decay of the two ESA bands in the TA spectra and global fitting of the broad ESA bands in the TRIR spectra.

**Figure 7:**
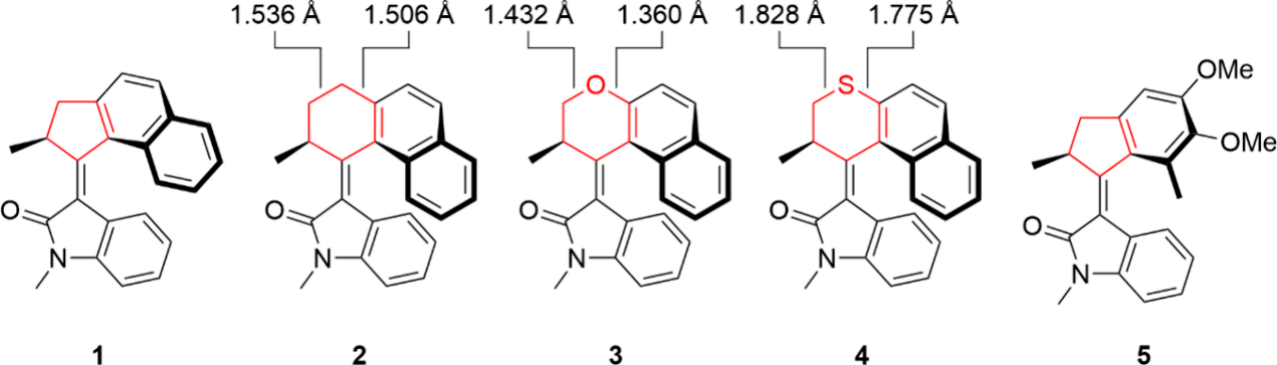
Structures of *N*-methyl oxindole motors **1**–**5** with the cycloalkene moiety highlighted in red. The bond lengths shown were calculated using isolated ground-state molecules optimized at the ωB97XD/6–31+G­(d,p) level of theory.

**Figure 8:**
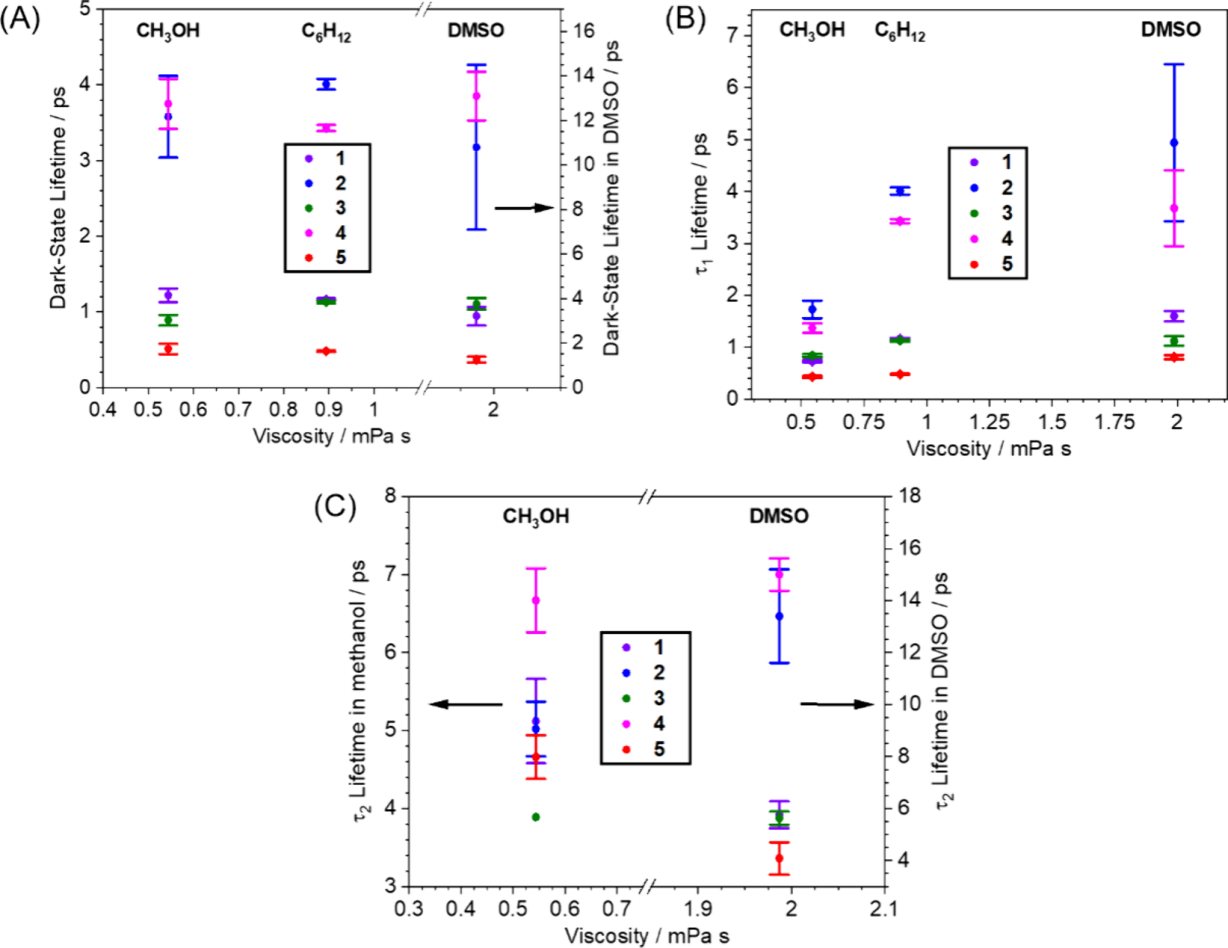
(A) The average dark state lifetimes (τ_DS_) of motors **1**–**5** are plotted against the viscosity of the solvents: methanol (0.544 mPa s, left), cyclohexane (0.894 mPa s, central) and DMSO (1.987 mPa s, right), highlighting the influence of solvent friction on the rate of decay of the dark state. The dark state decay kinetic data obtained in DMSO are plotted on a separate axis (right) for clarity. (B) The τ_1_ lifetimes of motors **1**–**5** are plotted against the viscosity of the solvents: methanol (left), cyclohexane (central) and DMSO (right). (C) The τ_2_ lifetimes of motors **1**–**5** are plotted against the viscosity of the solvents methanol (left) and DMSO (right). The τ_2_ decay kinetic data are plotted on separate axes for the two solvents.

For all five motors, the decay component characterized by τ_2_ is not found for solutions in the nonpolar cyclohexane, but it is always observed in both polar DMSO and methanol solvents. These consistent dynamics suggests that all the oxindole motors share the same solvent-dependent excited-state behavior, where the direct IC from S_1_ → S_0_ is dominant in the nonpolar cyclohexane, and the indirect S_1_ → S_0_ dynamics are only observed in more polar and viscous solvents. For all five oxindole motors, in DMSO the major component of the biexponential decay of the dark state involves the slower kinetics characterized by τ_2_, whereas for methanol solutions the τ_1_ component is the more important. The τ_1_ time constant, attributed to direct IC to the ground state, is the shortest in methanol when compared to both cyclohexane and DMSO for motors **1** – **4.**

The average dark-state lifetime increases by a factor of ≥ 2 in DMSO compared to cyclohexane and methanol for motors **1** – **4**, while the increase in the dark-state lifetime going from methanol to cyclohexane is marginal. In both these latter two solvents, direct approach to the S_1_/S_0_ CI is typically the dominant, or only relaxation pathway. Interestingly, motor **5** displays the weakest solvent dependence of the five motors studied, but is consistently the most rapid to relax from S_1_ to S_0_. This motor was designed to possess greater excited-state charge-transfer character than oxindole motors **1** – **4**, so its dynamics might be expected to be more sensitive to solvent polarity. Previously reported computational studies for motor **5** suggests that it exhibits less twist-pyramidalization motion than the other oxindole motors,^15^ and instead has more bond length alteration (BLA) character. Fluorene motor designs show greater precessional rotation compared to the oxindole motors **1** – **4** which rotate more axially, and motor **5** has the smallest amount of precession of the oxindole motors. These different rotational dynamics for **5** may be more volume conserving, thereby reducing the effect of solvent viscosity on the dark state lifetime.

There is a trend relating the relative steric bulk of the cycloalkene moiety of the rotor component in each motor (highlighted in red in Figure 7) to its dark state lifetime. For motors with added steric strain in the cycloalkene ring of the rotor there are increases in τ_1_, τ_2_ and the average lifetime of the dark state. Looking at Figure 8, the rates of excited-state decay generally follow **5** < **1** ≈ **3** < **2** ≈ **4**. The two fastest decaying motors both have a five-membered cyclopentene ring connected to the central alkene bond. This five-membered ring is less flexible than a six-membered ring due to a lack of conformational freedom. The motor **5** has significantly faster excited-state decay lifetimes than the other motors, which could be due to a combination of the enhanced BLA rotational mechanism and to the relative size of the rotor unit because this is the only motor studied that does not contain the large naphthyl group. The ether-containing motor **3** does possess a six-membered dihydro-pyran ring, yet its excited-state decay is almost as rapid as the **1** motor. The two motors with the consistently slowest decay kinetics are the **2** and **4** motors, which have the greatest degree of strain of the rotor ring due to the additional axial hydrogen of the methylene moiety of motor **2** and the longer C–S bonds of motor **4.**

For a first-generation motor, ultrafast time-resolved fluorescence experiments established a clear relationship between the biexponential excited-state lifetimes and the polarity and the viscosity of the chosen solvent.^41^ This study showed that nonpolar solvents introduce an energetic barrier on the S_1_ adiabatic PES that impedes the excited-state isomerization reaction. In contrast, higher polarity solvents expedite the decay of the dark state because a charge-transfer state is formed upon pyramidalization of the central alkene bond. The polar solvent molecules solvate the partial charges that develop across the alkene bond, thereby reducing the energy barrier in the S_1_ state, accelerating the rate of IC to S_0_, and reducing the lifetime of the excited state. This explanation is consistent with previous work on ethylene and stilbene.^28–31^ This prior work also noted that more viscous solvents lead to an increase in the excited-state lifetime. Another time-resolved study of the fluorene-based motor **6** reported similar results to those found here; the dark state decayed biexponentially, and there was little difference observed in the dark-state decay kinetics when comparing solutions in cyclohexane to ethanol. However, when comparing these results to a more viscous solvent decalin, there was a marked increase in both the dark-state decay time constants τ_1_ and τ_2_.

For the motor molecules **1**, **3** and **5** studied here, there is a slight decrease in the τ_1_ lifetimes when comparing cyclohexane (ε = 2.02 at 20 °C) solutions to methanol (ε = 33.0 at 20 °C) solutions (Figure 8). However, for motors **2** and **4** this decrease is more marked. This analysis suggests that the excited-state dynamics responsible for the τ_1_ lifetime are more sensitive to the solvent polarity for **2** and **4** when compared to the other oxindole motors. Pooler et al. showed that the isomerization mechanism of the oxindole class of motors differs from those of the fluorene motors, with a diminished degree of pyramidalization and with greater axial rotation in place of the precession seen for the fluorene motors. A lower degree of pyramidalization reduces the zwitterionic character across the olefinic bond at the S_1_ minimum geometry.^15^ The evidence presented here for the oxindole motors generally agrees with these findings. The **1**, **3** and **5** motors show a general independence of their τ_1_ lifetimes, which would suggest that their isomerization mechanisms are more axial and include less zwitterionic character. However, the **2** and **4** motors show a greater degree of solvent polarity dependence which would suggest that their isomerization mechanisms may be closer to the twist and pyramidalization mechanism that was previously observed for the fluorene motor **6.**

### Coherent Oscillatory Dynamics

For motors **2** – **4**, the TA measurements reveal damped oscillations in the early time (<2 ps) decay kinetics. These oscillations are observed in the ESA bands at visible wavelengths where the ground-state molecules do not absorb, so are attributed to S_1_ population. Examples are shown in Figure 9 and in the SI (Figures S43 – S45), with fits to derive estimated frequencies, the accuracies of which are limited by the temporal resolution of our measurements. To estimate the frequencies, the time-dependent integrated band intensities were fitted using an exponential decay function. The residuals from these exponential fits were then fitted using a damped sine function. The resulting values are reported in Table 3 for cyclohexane and DMSO solutions. Similar oscillations have been reported from previous time-resolved measurements for motor **6**, finding two oscillations with wavenumbers of 113 ± 7 cm^–1^ and 180 ± 12 cm^–1^,^7,15,22,33^ and motor **5** extracting values of 190 ± 10 cm^–1^ in methanol and 185 ± 12 cm^–1^ in hexane.^24^ The oscillations have been attributed to coherent vibrational motions in the electronically excited state which rapidly dephase. In the case of both **5** and **6**, the oscillations were assigned to a pyramidalization mode, which has been shown to be an important coordinate for motion from the FC state toward the dark state. Therefore, this is a plausible assignment for the oscillations observed here for motors **2** – **4**. In the TA spectra reported here, no oscillations were observed for motors **1** and **5** which could be because of insufficient temporal resolution in the time-resolved experiments, or reflect a smaller displacement of the pyramidalization coordinate, which is consistent with a more axial isomerization mechanism.

**Figure 9:**
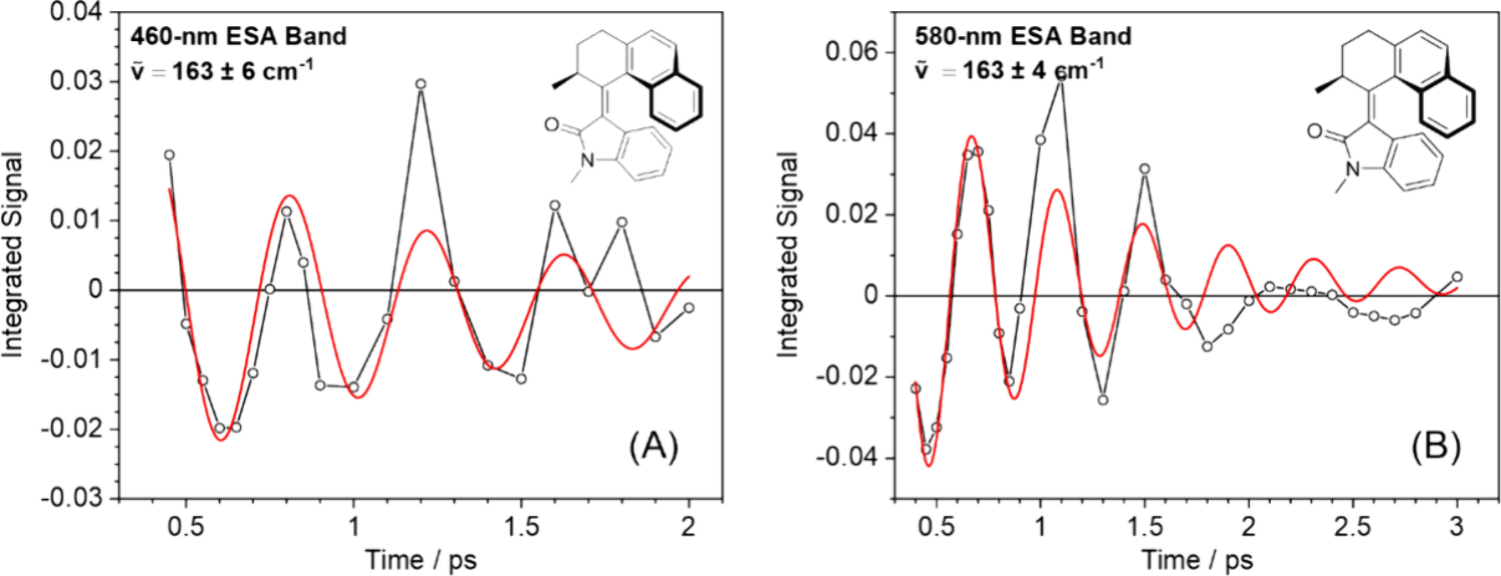
Oscillatory dynamics of motor **2** in DMSO. Damped sine function fits (red) are to the oscillatory components (black) of the integrated intensities of the ESA bands centered at (A) 460 and (B) 580 nm.

**Table 3:** Wavenumbers (in cm^–1^) of the Observed Damped Oscillations Extracted from TA ESA Decay Kinetics

	**2**/cm^–1^	**3**/cm^–1^	**4**/cm^–1^
cyclohexane		219 ± 7^a^	214 ± 4^a^
		221 ± 7^b^	201 ± 5^b^
DMSO	163 ± 6^a^	277 ± 8^a^	
	163 ± 4^b^	273 ± 10^b^	

a Shorter-wavelength and.

b Longer wavelength ESA bands.

### Isomerization Quantum Yields

The recovery of GSB features in our TRIR spectra provides a direct probe of the photoinduced isomerization efficiencies of the oxindole motors **1** – **5** if the photoproduct (*M*,*M*)-*Z* and starting (*P*,*P*)-*E* diastereomers have distinguishable IR absorption bands, or if the photoproduct (*M*,*M*)-*Z* diastereomer has a smaller IR transition moment for a given mode. Accurately determining Φ_iso_ values for overcrowded alkene motors using other methods is difficult because of their overlapping UV–vis absorption bands and the short lifetimes of the metastable diastereomers. Although isomerization quantum yields can be estimated using UV–vis spectroscopy under constant sample irradiation by a pump light with a known photon flux, the overlapping absorption spectra of the stable and metastable diastereomers for many motor designs mean that both forward and backward isomerization reactions are promoted, leading to a PSS and a lower-limit estimation of the Φ_iso_. This method also relies on using a calculated molar absorption coefficient for the metastable diastereomer because it is impractical to isolate enough of this pure compound to measure its UV–vis spectrum experimentally. An added complication for metastable diastereomers of some motor molecules is their short half-lives for THI, requiring the sample solution to be cooled during quantitative spectroscopic analysis.

Here, we use an alternative technique to quantify the Φ_iso_ from the time-dependent intensities of the carbonyl GSB in the TRIR spectra. The carbonyl vibrational transition is detected as a sharp GSB band at higher wavenumber than the broad ESA bands, and these two contributions to the TRIR spectra are typically not superimposed. The isolated GSB features were fitted using a Gaussian function to integrate their band intensities, and the fractional degree of parent diastereomer recovery was then readily determined from TRIR spectra obtained at time delays when all the excited-state dynamics and vibrational cooling are complete. This analysis reports the fractional loss of the initial diastereomer, but can be used to estimate the quantum yield for isomerization if no other photochemical pathways are open. The estimation of Φ_iso_ is valid if the corresponding carbonyl band of the photoproduct metastable diastereomer is sufficiently shifted to avoid spectral overlap. Using the calculated frequencies corresponding to the carbonyl stretching mode for each motor (Table 4), we see that for most of the motors studied these bands should be well separated for the starting (*P*,*P*)-*E* and photoproduct (*M*,*M*)-*Z* diastereomers. However, for motors **1** and **5** the difference in the calculated frequencies for the carbonyl stretch in the two diastereomers is <5 cm^–1^, meaning the isomerization yield values estimated for these molecules could be smaller than the true Φ_iso_ values.

**Table 4:** Calculated Wavenumbers (in cm^–1^) of the Carbonyl Stretch Mode for the Stable (*P*,*P*)-*E* Diastereomer and the Metastable (*M*,*M*)-*Z* Diastereomer of Each Motor^a^

	**1**/cm^–1^	**2**/cm^–1^	**3**/cm^–1^	**4**/cm^–1^	**5**/cm^–1^
stable *E*	1704.1	1704.0	1692.5	1704.7	1703.4
metastable *Z*	1706.1	1715.5	1713.6	1719.5	1699.3
ΔIR wavenumber	1.9	11.6	21.1	14.8	4.2

a A scaling factor of 0.952 was used to correct for anharmonicity. This value was chosen for the wB97XD/631+G­(d,p) functional and basis set used for these calculations in accord with the NIST Computational Chemistry Comparison and Benchmark Database.^44^

Table 5 shows the results of this quantum yield estimation. The reported values confirm that the initial photochemical isomerization step is inefficient, with more than 90% of molecules returning to the initial diastereomer for all the motors. The highest recorded quantum yield measured is 8.7% for motor **5** in methanol, in agreement with Φ_iso_ = 8% obtained using the methodology outlined by Stranius and Börjesson and by the Feringa group.^15,42^ This result suggests that even though there may be some spectral overlap of the carbonyl stretching mode IR absorption bands between the (*P*,*P*)-*E* and (*M*,*M*)-*Z* diastereomers, our GSB-recovery technique provides an accurate determination of Φ_iso_.

**Table 5:** Solvent-Dependent Quantum Yields for Isomerization (Φ_iso_) for *N*-Methyl Oxindole Motors **1**–**5** ^a^

	Φ_iso_/%				
	1	2	3	4	5
cyclohexane	2.7	6.5	2.2	2.9	6.2
DMSO	3.0	0.4	3.7	0.2	3.5
methanol	5.6	6.6	4.2	2.5	8.7

a Values were obtained by analysis of the recovery of the carbonyl GSB features in TRIR spectra.

In general, the data in Table 4 show that the lowest isomerization quantum yield for each motor is recorded in DMSO and the highest is found in methanol. However, this is not a perfect trend. The lowest isomerization quantum yield for the motors **1** and **3** was obtained in cyclohexane solution, whereas for **4** the highest recorded quantum yield was found in cyclohexane solution.

The lowest isomerization quantum yields determined were for motors **2** and **4** dissolved in DMSO. Interestingly, these are the two motor-solvent combinations with the longest average dark-state lifetimes, largest τ_2_ time constants for all of the motors studied, and the largest proportion of the τ_2_ time component of the biexponential decay kinetics. A previous time-resolved study highlighted a correlation between the dark-state lifetime and quantum yield for overcrowded alkene motors with a fluorene stator, finding that the motors with longer dark-state lifetimes have greater Φ_iso_ values.^7^ For oxindole motors **2** and **4** in DMSO, the opposite is observed. The previous study compared analogues of the fluorene motor **6** with varying functional group substitution at the 5′ position in the same solvent, whereas in the current study the elongated dark-state kinetics are not induced by chemical substitution, but instead by use of a more viscous solvent. A possible explanation for the suppressed isomerization quantum yield in DMSO could be greater solvent friction impeding the motion of the rotor, leading to a bifurcation upon IC to the ground state that promotes more of the excited-state molecules to return to the starting diastereomer (*P*,*P*)-*E* instead of forming the isomerization photoproduct (*M*,*M*)-*Z.*

The Φ_iso_ = 0.3% value determined here for **2** dissolved in DMSO and irradiated with 345 nm light is lower than the previously reported 2.3% when irradiated with 420 nm light in the same solvent and with almost identical concentration.^14^ The two methods for estimating Φ_iso_ values are very different, and come with significant uncertainties; nevertheless, this comparison suggests that although the longer wavelength irradiation leads to a less favorable PSS, it could also result in a higher Φ_iso_. Verification that the Φ_iso_ values are wavelength dependent could come from further TRIR measurements using 420 nm excitation.

## Conclusions

Ultrafast TA and TRIR spectroscopic study of *N*-methyl oxindole motors **1** – **5** revealed their excited-state dynamical behavior, relaxation time scales, and isomerization quantum yields. Measurements were conducted in three solvents (cyclohexane, methanol and DMSO) to explore how solute–solvent interactions influence the photoisomerization. The early time kinetics are similar to those for fluorene-based molecular motors, with the excited state *N*-methyl oxindole motor molecules evolving in the adiabatic S_1_ excited state from the Franck–Condon region to a region of different electronic character in the vicinity of the S_1_ minimum energy geometry in <1 ps. A linear interpolation of potential energies in the S_0_, S_1_ and S_2_ states supports this picture of adiabatic dynamics toward the S_1_ state minimum before nonadiabatic relaxation to the S_0_ state, in a dark region of the adiabatic S_1_ excited state with low oscillator strength for radiative decay back to S_0_.

The dark state lifetime depends on both the polarity and viscosity of the surrounding solvent. For photoexcitation in cyclohexane, the decay of the dark state population is monoexponential, with a time constant indicative of direct dynamics from the FC region to the seam of S_1_ – S_0_ conical intersections. In contrast, in polar solvents, two time constants describe the dark state lifetime. These two distinct decay time scales are attributed to direct and indirect mechanisms for approach to the S_1_/S_0_ CI, the latter caused by solvent-induced vibrational energy redistribution and quenching as confirmed by shifts in the ESA bands observed by TRIR. The greater the steric bulk of the rotor component of the oxindole motor, the longer is the dark state lifetime. Coherent oscillations were observed in the ESA bands of the TA spectra for motors **2** – **4**, and are attributed to low-frequency vibrational motions orthogonal to the motor isomerization coordinate in the S_1_ state. The extent of GSB recovery in TRIR spectra gives a direct estimate of the quantum yield for the first photoinduced *E* → *Z* isomerization step, Φ_iso_, for these motors. These yields are all small (<9% for all examples studied) and are sensitive to the choice of solvent.

## Supplementary Material



## Data Availability

Data are available at the University of Bristol data repository, data.bris, at 10.5523/bris.37ojctet2tx4326o67jiq5ooid.

## Notes

Additional experimental and computational details for the study of motors **1**–**5** in three solvents; general experimental procedures, synthetic protocols, and characterization data including NMR, UV–vis, and FTIR spectra; further transient absorption (TA) and time-resolved infrared (TRIR) experimental data, including spectral decomposition and kinetic analyses; and fitted oscillatory dynamics of excited-state species and theoretical insights from TD-DFT calculations of interpolated oxindole motor structures (PDF)
