# Correction to “Solvent-Dependent Ultrafast Photochemical Dynamics of *N*‑Methyl Oxindole Overcrowded Alkene Molecular Motors”

**DOI:** 10.1021/acs.jpca.6c04048

**Published:** 2026-07-01

**Authors:** Connah J. Harris, Beatrice S. L. Collins, Andrew J. Orr-Ewing

The published manuscript incorrectly acknowledged EPSRC grant EP/S024107. The corrected acknowledgments can be found below.

