# Supplementary material for: Solvent-Dependent Ultrafast Photochemical Dynamics of N‑Methyl Oxindole Overcrowded Alkene Molecular Motors
Source: J Phys Chem A. 2025 Jun 16;129(25):5530–44. doi: 10.1021/acs.jpca.5c02679 (PMC12207583; doi:10.1021/acs.jpca.5c02679)
Supplement: Supplementary file 1 [file jp5c02679_si_001.pdf]

Supporting Information:

Solvent-Dependent Ultrafast Photochemical Dynamics of *N*-Methyl Oxindole Overcrowded Alkene Molecular Motors

Connah J. Harris, Beatrice S. L. Collins and Andrew J. Orr-Ewing*

School of Chemistry, University of Bristol, Cantock's Close, Bristol BS8 1TS, United Kingdom

* a.orr-ewing@bristol.ac.uk

Table of Contents

S1 General Experimental Details S3

S1.1 Solvents, reagents and starting materials S3

S1.2 Chromatography S3

S1.3 Nuclear magnetic resonance spectroscopy S3

S1.4 UV-Vis spectroscopy S3

S1.5 Naming of Compounds S3

S2 Synthetic Procedures S4

S3 NMR Spectra S19

S4 Further TA and TRIR Experimental S33

S5 Normalized UV-Vis Spectra of the Oxindole Motors S34

S6 FTIR Spectra of the Oxindole Motors S38

S7 TA and TRIR Spectra and Kinetics of the Oxindole Motors S43

S7.1 Motor 1 in cyclohexane, DMSO and methanol solutions S43

S7.2 Motor 2 in cyclohexane, DMSO and methanol solutions S46

S7.3 Motor 3 in cyclohexane, DMSO and methanol solutions S49

S7.4 Motor 4 in cyclohexane, DMSO and methanol solutions S52

S7.5 Motor 5 in cyclohexane, DMSO and methanol solutions S55

S8 Examples of TA and TRIR Spectral Decomposition of the Spectra S58

S9 Kinetics of Excited-State Relaxation for the Oxindole Motors S61

S9.1 Motor 1 in cyclohexane, DMSO and methanol solutions S61

S9.2 Motor 2 in cyclohexane, DMSO and methanol solutions S64

S9.3 Motor 3 in cyclohexane, DMSO and methanol solutions S67

S9.4 Motor 4 in cyclohexane, DMSO and methanol solutions S70

S9.5 Motor 5 in cyclohexane, DMSO and methanol solutions S73

S10 Summary of the Measured Excited-State Kinetics of the Oxindole Motors S76

S11 Fitted Oscillatory Dynamics of the TA ESA Bands S78

S12 Characterization of the Excited-State PESs Using TD-DFT Calculations of Interpolated Oxindole Motor Structures S80

S13 References S82

S1 General Experimental Details

S1.1 Solvents, reagents and starting materials

For the synthesis of *N*-methyl oxindole motors **1** – **5**, all reactions with air- or moisture-sensitive materials were performed under a N_2 atmosphere with dried glassware using Schlenk techniques. Unless otherwise stated, all reagents and solvents were obtained from commercial sources and used as received. Water is de-ionized and brine refers to a saturated solution of an aqueous solution of NaCl. Solutions were evaporated under reduced pressure using a Büchi or IKA rotary evaporator. Select dry solvents (CH_2Cl_2, THF, MeCN and hexane) were provided by the University of Bristol School of Chemistry Grubbs apparatus (Anhydrous Engineering Ltd. modified Grubbs system of double alumina and alumina-copper catalysed drying columns).

S1.2 Chromatography

Flash column chromatography was carried out using silica gel (SiO_2, 40 – 63 µm) and was purchased from Sigma-Aldrich. Where possible, reactions were followed by thin-layer chromatography (TLC) using Merck Kieselgel 60 F_{254} fluorescent treated silica gel, which was visualised under a UV lamp at 256 nm or by staining with aqueous basic potassium permanganate.

S1.3 Nuclear magnetic resonance spectroscopy

^1H and ^{13}C nuclear magnetic resonance (NMR) spectra were recorded on Jeol JNM-ECZ 400 MHz, Varian CNMR 400 MHz and Bruker Advance DPX 400 MHz and Bruker Avance III HD 500 MHz equipped with CryoProbe spectrometers at 25 °C. Chemical shifts are given in parts per million (ppm) and referenced to: $CHCl_3$ (δ 7.26 ppm for ^1H), $CDCl_3$ (δ 77.2 ppm for ^{13}C), CD_3CN (δ 1.94 ppm for ^1H), CHD_2CD_3SO (δ 2.50 ppm for ^1H) and $(CD_3)_2SO$ (δ 39.52 ppm for ^{13}C). Coupling constants (*J*) are quoted in Hertz (Hz) and refer to multiplicities (s = singlet, br. s = broad singlet, d = doublet, t = triplet, q = quartet, quin = quintet, sex = sextet, h = heptet, m = multiplet, dd = doublet of doublets, etc.). The ^1H NMR data are reported as follows: chemical shift (multiplicity, coupling constants, number of protons, proton assignment).

S1.4 UV-Vis spectroscopy

All spectra were recorded on a Thermo Scientific Genesys 10S UV-Vis spectrophotometer using a Harrick cell. The sample concentrations and spacers used are reported in Table S1.

S1.5 Naming of Compounds

Compound names were generated by ChemBioDraw 20.0 software (PerkinElmer), following the IUPAC nomenclature.

S2 Synthetic Procedures

The oxindole motors **1** – **5** were synthesized following established procedures from the Feringa group,[2,8,14,15,34–36] and the corresponding synthetic routes are illustrated in Scheme S1.

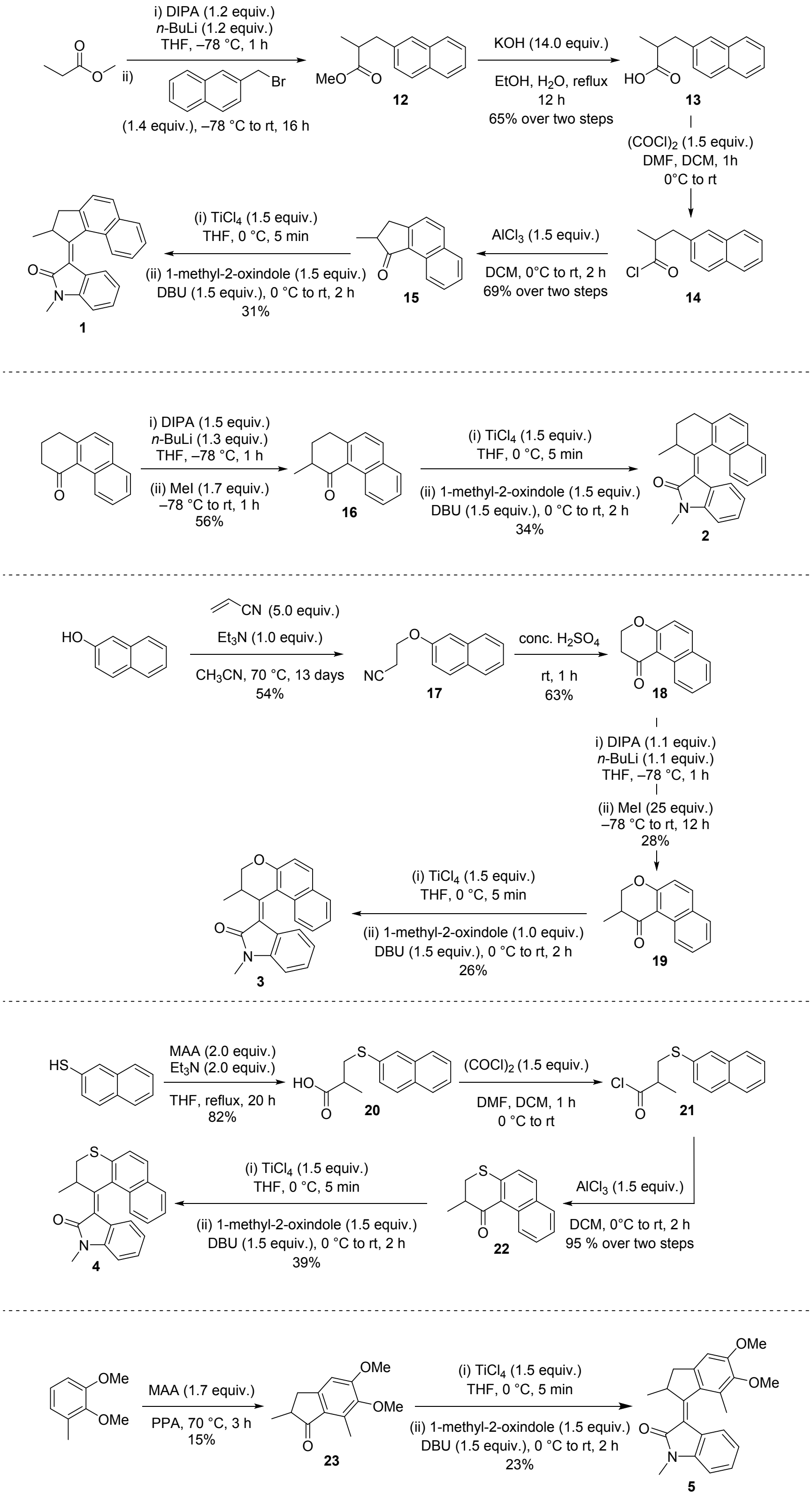

Scheme S1: Synthesis of oxindole motors **1** – **5**.

Methyl 2-methyl-3-(naphthalen-2-yl)propanoate 12

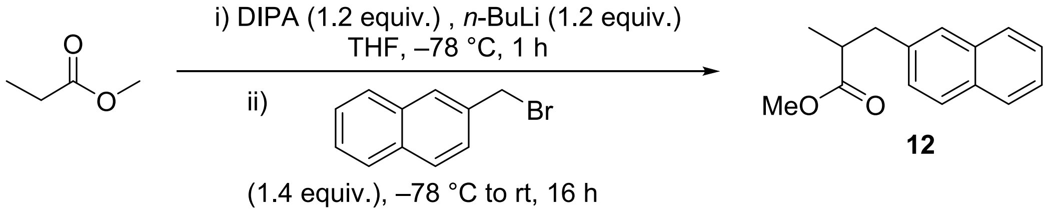

To a solution of diisopropylamine (0.84 mL, 6.0 mmol) in THF (30 mL) at –78 °C was added *n*-BuLi (1.6 M in hexane, 3.75 mL, 6.0 mmol) dropwise and the solution was stirred at –78 °C for 15 minutes. After this time, methyl propanoate (0.48 mL, 5.0 mmol) was added dropwise and the resulting mixture was stirred for 1 h at –78 °C. After this time, a solution of 2-(bromomethyl)naphthalene (1.55 g, 7.00 mmol) in THF (10 mL) was added dropwise, and the reaction mixture allowed to warm to rt. After stirring for 16 h at rt, the reaction was quenched by the addition of a saturated aqueous solution of NH_4Cl (100 mL), and the aqueous phase was extracted with Et_2O (3 × 30 mL). The organic phases were combined, dried over $MgSO_4$, filtered and concentrated under reduced pressure to yield **12** as a yellow oil. The crude product was carried forward without purification.

2-Methyl-3-(naphthalen-2-yl)propanoic acid 13

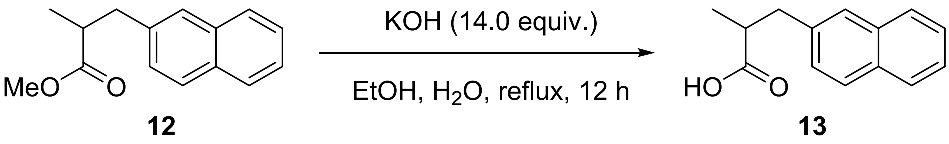

Crude methyl 2-methyl-3-(naphthalen-2-yl)propanoate **12** (5.0 mmol) was treated with potassium hydroxide (4.0 g, 71.3 mmol), ethanol (20 mL) and water (20 mL) and heated to reflux for 12 h. After this time the reaction mixture was acidified with 30% HCl and extracted with Et_2O (3 × 20 mL). The combined organic phases were extracted with 2 M NaOH solution, and the combined aqueous phases were then reacidified using 1M HCl and subsequently extracted with Et_2O (3 × 20 mL). The combined organic phases were then concentrated under reduced pressure to yield 2-methyl-3-naphthalen-2-ylpropionic acid **13** as a white solid (0.70 g, 3.25 mmol, 65% over two steps).

mp: 89.2 – 90.6 °C. **^{1}H NMR** (400 MHz, $CDCl_3$) δ 7.84 – 7.75 (m, 3H), 7.64 (s, 1H), 7.51 – 7.40 (m, 2H), 7.33 (dd, *J* = 8.4, 1.7 Hz, 1H), 3.31 – 3.19 (m, 1H), 2.94 – 2.80 (m, 2H), 1.22 (d, *J* = 6.6 Hz, 3H). Carboxylic acidic proton was not observed due to the fast exchange time. **^{13}C NMR** (101 MHz, $CDCl_3$) δ 182.0, 136.7, 133.6, 132.4, 128.2, 127.8, 127.7, 127.6, 127.5, 126.2, 125.6, 41.2, 39.6, 16.7.

Analytical data are in agreement with the literature.[1]

2-Methyl-3-(naphthalen-2-yl)propanoyl chloride 14

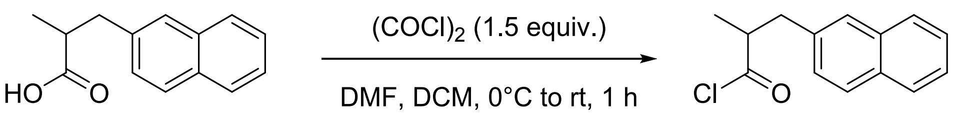

A solution of 2-methyl-3-naphthalen-2-ylpropionic acid **13** (0.68 g, 3.2 mmol) in DCM (30 mL) was cooled to 0 °C and treated with a drop of DMF followed by the dropwise addition of oxalyl chloride (0.41 mL, 4.8 mmol). The mixture was allowed to warm to rt and stirred for 1 h. After this time, the solvent and excess oxalyl chloride were removed under reduced pressure to provide the crude product 2-methyl-3-naphthalen-2-ylpropionyl chloride **14** as a brown oil. The crude product was carried forward without purification.

2-Methyl-2,3-dihydro-1*H*-cyclopenta[*a*]naphthalen-1-one 15

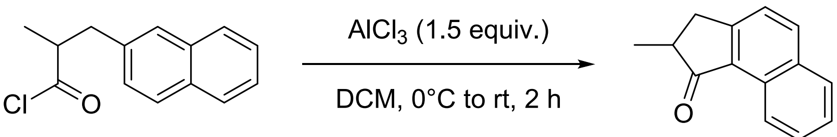

To a solution of crude 2-methyl-3-naphthalen-2-ylpropionyl chloride **14** (3.2 mmol) in DCM (30 mL) at 0 °C was carefully added $AlCl_3$ (0.63 g, 4.8 mmol) portion wise. After the addition, the mixture was allowed to warm to rt and stirred for 2 h. The mixture was then re-cooled to 0 °C and the reaction was carefully quenched with H_2O (50 mL). The organic phase was separated, and the aqueous phase was extracted with DCM (3 x 30 mL). The combined organic phases were washed with $NaHCO_{3(aq)}$ (50 mL), brine (50 mL), dried over Mg_2SO_4, filtered and concentrated under reduced pressure. The crude product was purified by column chromatography (SiO_2, hexane:EtOAc, 20:1) to yield 2,3-dihydro-2-methyl-1H-benz[e]inden-1-one **15** (0.44 g, 2.3 mmol, 69% over two steps) as an orange oil.

^{1}H NMR (400 MHz, $CDCl_3$) δ 9.16 (d, *J* = 8.4 Hz, 1H), 8.03 (d, *J* = 8.3 Hz, 1H), 7.88 (d, *J* = 8.3 Hz, 1H), 7.67 (ddd, *J* = 8.3, 7.0, 1.3 Hz, 1H), 7.55 (ddd, *J* = 8.3, 7.0, 1.3 Hz, 1H), 7.49 (d, *J* = 8.3 Hz, 1H), 3.47 (dd, *J* = 18.1, 8.0 Hz, 1H), 2.87 – 2.76 (m, 2H), 1.38 (d, *J* = 7.3 Hz, 3H). **^{13}C NMR** (101 MHz, $CDCl_3$) δ 210.2, 156.8, 135.9, 132.8, 130.3, 129.7, 129.0, 128.2, 126.7, 124.1, 124.1, 42.5, 35.5, 16.8.

Analytical data are in agreement with the literature.[2]

(*E*)-1-methyl-3-(2-methyl-2,3-dihydro-1H-cyclopenta[α]naphthalen-1-ylidene)indolin-2-one 1

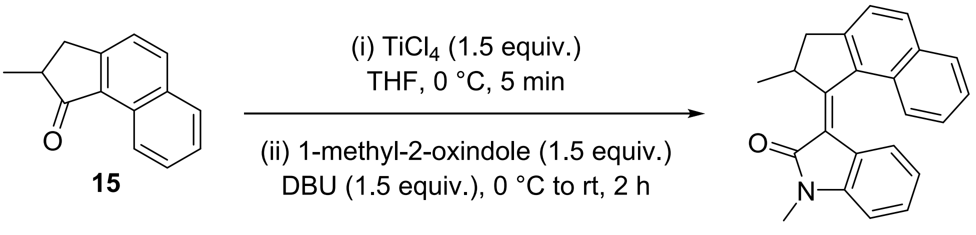

15 **1**

To a solution of 2,3-dihydro-2-methyl-1H-benz[e]inden-1-one **15** (0.098 g, 0.50 mmol) in THF (3 mL) at 0 °C was added titanium(IV) tetrachloride (0.75 mL, 1 M in toluene, 0.75 mmol) dropwise and the resulting solution was stirred for 5 minutes, after which the solution had turned dark orange. A solution of 1-methyl-2-oxindole (0.110 g, 0.75 mmol) in THF (3 mL) was then added dropwise to the reaction mixture at 0 °C, followed by 1,8-diazabicyclo[5.4.0]undec-7-ene (DBU) (112 µL, 0.75 mmol), after which a black precipitate formed. The reaction mixture was then allowed to warm to rt and stirred for a further 2 h. The reaction was then quenched with aqueous 1M HCl and the organic phase was separated and the aqueous phase was extracted with EtOAc (3 × 30 mL). The combined organic phases were washed with H_2O (30 mL), brine (30 mL), dried over $MgSO_4$, filtered and concentrated under reduced pressure. The crude mixture was purified using column chromatography (SiO_2, hexane:EtOAc, 5:1) to yield (*E*)-1-methyl-3-(2-methyl-2,3-dihydro-1H-cyclopenta[a]naphthalen-1-ylidene)indolin-2-one **1** (0.051 g, 0.16 mmol, 31%) as an orange solid.

mp: 151.5 – 154.7 °C. **^{1}H NMR** (500 MHz, $CDCl_3$) δ 7.99 (d, *J* = 8.7 Hz, 1H), 7.97 (d, *J* = 7.4 Hz, 1H), 7.62 (dd, *J* = 8.3 Hz, 1H), 7.58 (d, *J* = 8.3 Hz, 1H), 7.52 (ddd, *J* = 7.5, 6.8, 1.3 Hz, 1H), 7.42 (ddd, *J* = 7.5, 6.8, 1.3 Hz, 1H), 7.17 (td, *J* = 7.7, 1.2 Hz, 1H), 6.85 (d, *J* = 7.7, 1H), 6.65 (td, *J* = 7.7, 1.2 Hz, 1H), 6.39 (d, *J* = 7.7 Hz, 1H), 4.6 (quin, *J* = 6.6 Hz, 1H), 3.50 (dd, *J* = 15.6, 6.6 Hz, 1H), 3.37 (s, 3H), 2.77 (d, *J* = 15.7 Hz, 1H), 1.29 (d, *J* = 6.6 Hz, 3H). **^{13}C NMR** (126 MHz, $CDCl_3$) δ 168.9, 162.1, 150.7, 142.8, 135.0, 132.9, 132.8, 129.3, 129.0, 127.8, 127.6, 127.0, 125.8, 125.2, 124.2, 122.3, 120.7, 119.9, 107.3, 43.8, 41.7, 26.0, 19.6.

Analytical data are in agreement with the literature.[3]

3-Methyl-2,3-dihydrophenanthren-4(1H)-one 16

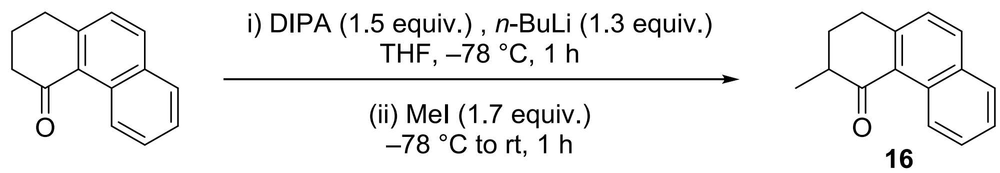

16

To a solution of diisopropylamine (0.54 mL, 3.85 mmol) in THF (15 mL) at 0 °C was added *n*-BuLi (1.6 M, 2.00 mL, 3.30 mmol) dropwise and the solution was stirred at 0 °C for 30 minutes. After this time, the reaction mixture was cooled to –78 °C and a solution of 2,3-dihydro-4(1*H*)-phenanthrenone (0.50 g, 2.52 mmol) in THF (10 mL) was added dropwise and the resulting mixture was stirred for 1 h at –78 °C. After this time, iodomethane (0.27 mL, 4.34 mmol) was added dropwise and the reaction was allowed to warm to rt and stirred at rt for a further 1 h. The reaction was then quenched with saturated aqueous NH_4Cl (15 mL) and extracted with Et_2O (3 x 30mL). The combined organic phases were washed with brine (30 mL), dried with anhydrous $MgSO_4$, filtered and concentrated under reduced pressure. The crude product was purified by column chromatography (SiO_2, hexane:EtOAc, 20:1) to yield 2,3-dihydro-3-methyl-4(*1H*)-phenanthrenone **16** (0.30 g, 1.40 mmol, 56%) as a yellow oil.

^{1}H NMR (400 MHz, $CDCl_3$) δ 9.35 (d, *J* = 8.7 Hz, 1H), 7.90 (d, *J* = 8.4 Hz, 1H), 7.80 (d, *J* = 8.2 Hz, 1H), 7.61 (ddd, *J* = 8.5, 6.8, 1.6 Hz, 1H), 7.48 (ddd, *J* = 8.0, 6.9, 1.3 Hz, 1H), 7.29 (d, *J* = 8.4 Hz, 1H), 3.22 (ddd, *J* = 17.5, 10.8, 4.9 Hz, 1H), 3.13 (dt, *J* = 17.3, 4.6 Hz, 1H), 2.82 – 2.71 (m, 1H), 2.30 – 2.22 (m, 1H), 2.04 – 1.91 (m, 1H), 1.32 (d, *J* = 6.7 Hz, 3H). **^{13}C NMR** (101 MHz, $CDCl_3$) δ 203.6, 146.0, 134.0, 132.9, 131.5, 128.8, 128.4, 127.4, 127.1, 126.7, 125.9, 44.0, 31.3, 30.5, 16.1.

Analytical data are in agreement with the literature.[4]

(*E*)-1-methyl-3-(3-methyl-2,3-dihydrophenanthren-4(1H)-ylidene)indolin-2-one 2

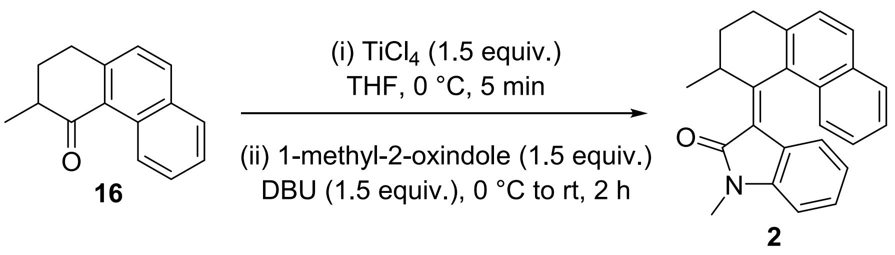

To a solution of 2,3-dihydro-3-methyl-4(*1H*)-phenanthrenone **16** (0.11 g, 0.50 mmol) in THF (3 mL) at 0 °C was added titanium(IV) tetrachloride (0.75 mL, 1 M in toluene, 0.75 mmol) dropwise and the resulting solution was stirred for 5 minutes, after which the solution had turned dark orange. A solution of 1-methyl-2-oxindole (0.110 g, 0.75 mmol) in THF (3 mL) was then added dropwise to the reaction mixture at 0 °C, followed by 1,8-diazabicyclo[5.4.0]undec-7-ene (DBU) (110 µL, 0.75 mmol), after which a black precipitate formed. The reaction mixture was then allowed to warm to rt and stirred for a further 2 h. The reaction was then quenched with aqueous 1M HCl and the organic phase was separated and the aqueous phase was extracted with EtOAc (3 × 30 mL). The combined organic phases were washed with H_2O (30 mL), brine (30 mL), dried over $MgSO_4$, filtered and concentrated under reduced pressure. The crude mixture was purified using column chromatography (SiO_2, hexane:EtOAc, 5:1) to yield (*E*)-1-methyl-3-(3-methyl-2,3-dihydrophenanthren-4(*1H*)-ylidene)indolin-2-one **2** (0.058 g, 0.17 mmol, 34%) as a bright yellow solid.

mp: 160.8 – 165.6 °C. **^{1}H NMR** (400 MHz, $CDCl_3$) δ 7.89 (d, *J* = 8.0 Hz, 1H), 7.79 (d, *J* = 8.5 Hz, 1H), 7.45 – 7.38 (m, 2H), 7.30 (ddd, *J* = 8.3, 6.8, 1.3 Hz, 1H), 7.03 (td, *J* = 7.7, 1.1 Hz, 1H), 6.74 (d, *J* = 7.7 Hz, 1H), 6.34 (td, *J* = 7.7, 1.0 Hz, 1H), 5.70 (d, *J* = 7.7 Hz, 1H), 4.97 (m, 1H), 3.33 (s, 3H), 2.78 (ddd, *J* = 14.5, 4.6, 3.1 Hz, 1H), 2.64 – 2.51 (m, 1H), 2.43 – 2.32 (m, 1H), 1.20 – 1.09 (m, 1H), 1.15 (d, *J* = 6.8 Hz, 3H). **^{13}C NMR** (101 MHz, $CDCl_3$) δ 168.0, 156.0, 142.5, 141.0, 132.1, 131.7, 130.5, 129.6, 128.7, 128.0, 127.2, 126.0, 125.3, 125.1, 123.6, 122.7, 122.2, 121.2, 107.3, 32.5, 29.5, 25.9, 20.9.

Analytical data are in agreement with the literature.[3]

3-(Naphthalen-2-yloxy)propanenitrile 17

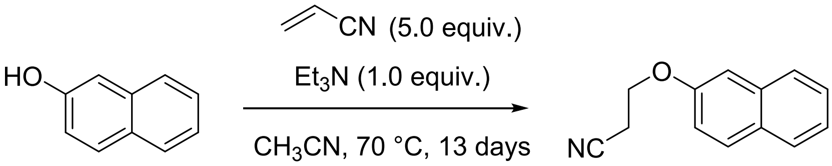

17

A solution of 2-naphthol (1.0 g, 6.9 mmol), acrylonitrile (2.3 mL, 35 mmol), and triethylamine (0.96 mL, 6.9 mmol) in acetonitrile (5.0 mL) was heated to 70 °C and stirred at this temperature for 13 days with the progress of the reaction being monitored by TLC. Upon completion of the reaction, the solution was poured into ice-cold water, precipitating a light red solid. The precipitate was filtered, dissolved in DCM (100 mL) and washed with 10% NaOH solution (3 x 30 mL). The combined aqueous washes were extracted with DCM (30 mL). The organic phases were combined and the solvent was removed under reduced pressure to yield a red solid. The solid was recrystallised from acetone to yield 3-(naphthalen-2-yloxy)propanenitrile **17** (0.73 g, 3.70 mmol, 54%).

mp: 110.6 – 112.2 °C. **^{1}H NMR** (400 MHz, $CDCl_3$) δ 7.82 – 7.71 (m, 3H), 7.47 (ddd, *J* = 8.2, 6.9, 1.3 Hz, 1H), 7.38 (ddd, *J* = 8.2, 6.9, 1.2 Hz, 1H), 7.17 (dd, *J* = 8.9, 2.6 Hz, 1H), 7.12 (d, *J* = 2.6 Hz, 1H), 4.32 (t, *J* = 6.4 Hz, 2H), 2.90 (t, *J* = 6.4 Hz, 2H). **^{13}C NMR** (101 MHz, $CDCl_3$) δ 155.8, 134.4, 130.0, 129.5, 127.9, 127.0, 126.8, 124.3, 118.7, 117.3, 107.2, 62.7, 18.7.

Analytical data are in agreement with the literature.[5]

2,3-Dihydro-1*H*-benzo[*f*]chromen-1-one 18

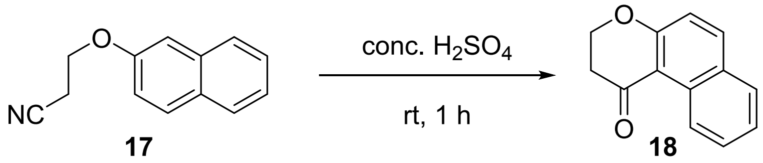

To a stirred solution of conc. H_2SO_4 (85% wt., 21.5 mL) was added 3-(naphthalen-2-yloxy)propanenitrile **17** (1.50 g, 7.6 mmol) in small portions over 1 h. The reaction mixture was stirred at rt for 1 h and subsequently poured onto ice. After warming up to rt, the solution was extracted with DCM (3 x 100 mL). The combined organic phases were washed with H_2O (100 mL), brine (100 mL), dried over $MgSO_4$, filtered and concentrated under reduced pressure affording a brown oil, which was purified by column chromatography (SiO_2, hexane:EtOAc = 85:15) to yield 2,3-dihydro-1*H*-benzo[*f*]chromen-1-one **18** (0.95 g, 4.8 mmol, 63%) as an orange oil.

^{1}H NMR (400 MHz, $CDCl_3$) δ 9.46 (d, *J* = 8.7 Hz, 1H), 7.91 (d, *J* = 9.0 Hz, 1H), 7.75 (d, *J* = 8.0 Hz, 1H), 7.64 (ddd, *J* = 8.5, 6.9, 1.5 Hz, 1H), 7.43 (ddd, *J* = 8.0, 7.0, 1.1 Hz, 1H), 7.10 (d, *J* = 9.0 Hz, 1H), 4.65 – 4.60 (m, 2H), 2.94 – 2.88 (m, 2H). **^{13}C NMR** (101 MHz, $CDCl_3$) δ 193.0, 164.0, 137.5, 131.7, 129.8, 129.3, 128.5, 126.1, 124.9, 118.9, 113.0, 67.1, 39.1.

Analytical data are in agreement with the literature.[6]

2-Methyl-2,3-dihydro-1*H*-benzo[*f*]chromen-1-one 19

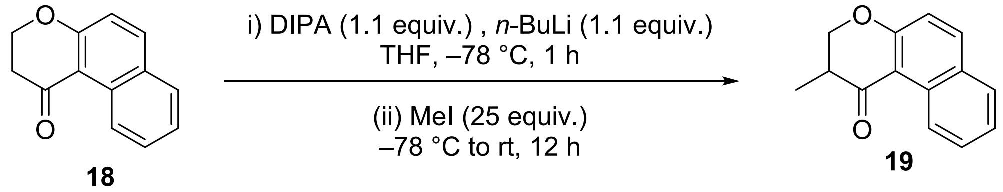

To a solution of diisopropylamine (0.7 mL, 5.3 mmol) in THF (2 mL) at −78 °C was added *n*-BuLi (1.6 M, 5.3 mmol, 3.3 mL) dropwise and the solution was stirred at −78 °C for 30 minutes. After this time, a solution of 2,3-dihydro-1*H*-benzo[*f*]chromen-1-one **18** (0.95 g, 4.8 mmol) in THF (6.0 mL) was added dropwise and the resulting mixture was stirred for 5 minutes at –78 °C. After this time, iodomethane (7.5 mL, 120 mmol) was added dropwise and the reaction was allowed to warm to rt and stirred at rt for a further 12 h. The solvent was then removed under reduced pressure and the crude product was re-dissolved in DCM (200 mL), washed with H_2O (200 mL) and the organic phases was dried over $MgSO_4$, filtered and concentrated under reduced pressure. The obtained yellow oil was purified by column chromatography (SiO_2, hexane:EtOAc, 20:1) to yield 2-methyl-2,3-dihydro-1*H*-benzo[*f*]chromen-1-one **19** (0.6 g, 2.7 mmol, 28%) as a yellow oil.

^{1}H NMR (400 MHz, $CDCl_3$) δ 9.48 (d, *J* = 8.7, Hz, 1H), 7.90 (d, *J* = 8.9 Hz, 1H), 7.74 (d, *J* = 8.0 Hz, 1H), 7.63 (ddd, *J* = 8.5, 7.0, 1.5 Hz, 1H), 7.42 (ddd, *J* = 8.1, 7.0, 1.2 Hz, 1H), 7.09 (d, *J* = 9.0 Hz, 1H), 4.59 (dd, *J* = 11.2, 5.1 Hz, 1H), 4.26 (t, *J* = 10.9 Hz, 1H), 2.99 – 2.88 (m, 1H), 1.28 (d, *J* = 7.0 Hz, 3H). **^{13}C NMR** (101 MHz, $CDCl_3$) δ 196.2, 163.7, 137.3, 131.9, 129.6, 129.4, 128.5, 126.0, 124.9, 118.8, 112.1, 72.2, 41.4, 11.4.

Analytical data are in agreement with the literature.[6]

(*E*)-1-Methyl-3-(2-methyl-2,3-dihydro-1*H*-benzo[*f*]chromen-1-ylidene)indolin-2-one 3

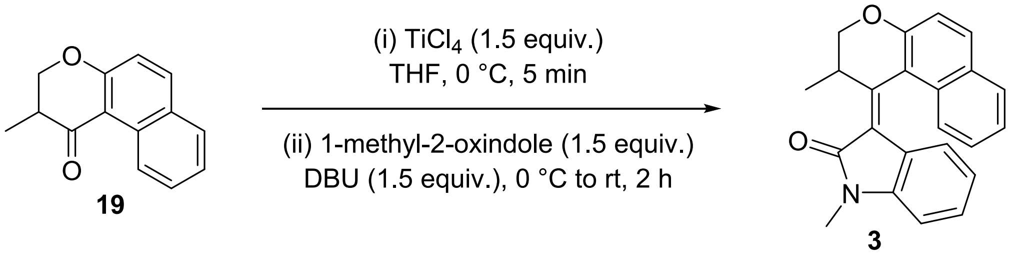

To a solution of 2-methyl-2,3-dihydro-1*H*-benzo[*f*]chromen-1-one **19** (0.43 g, 2.00 mmol) in THF (12 mL) at 0 °C was added titanium(IV) tetrachloride (3.00 mL, 1 M in toluene, 3.00 mmol) dropwise and the resulting solution was stirred for 5 minutes, after which the solution had turned dark orange. A solution of 1-methyl-2-oxindole (0.44 g, 3.00 mmol) in THF (12 mL) was then added dropwise to the reaction mixture at 0 °C, followed by 1,8-diazabicyclo[5.4.0]undec-7-ene (DBU) (0.45 mL, 3.00 mmol), after which a black precipitate formed. The reaction mixture was then allowed to warm to rt and stirred for a further 2 h. The reaction was then quenched with aqueous 1M HCl and the organic phase was separated and the aqueous phase was extracted with EtOAc (3 × 30 mL). The combined organic phases were washed with H_2O (30 mL), brine (30 mL), dried over $MgSO_4$, filtered and concentrated under reduced pressure. The crude mixture was purified using column chromatography (SiO_2, hexane:EtOAc, 10:1) to yield (*E*)-1-methyl-3-(2-methyl-2,3-dihydro-1*H*-benzo[*f*]chromen-1-ylidene)indolin-2-one **3** (0.17 g, 0.51 mmol, 26%) as an orange solid.

mp: 138.4 – 140.7 °C. **^{1}H NMR** (400 MHz, $CDCl_3$) δ 7.85 (d, *J* = 8.5 Hz, 1H), 7.80 (dd, *J* = 8.1, 1.0 Hz, 1H), 7.58 (d, *J* = 8.5 Hz, 1H), 7.31 (ddd, *J* = 8.1, 6.9, 1.0 Hz, 1H), 7.23 (ddd, *J* = 8.5, 6.9, 1.5 Hz, 1H), 7.13 – 7.07 (m, 2H), 6.78 (d, *J* = 7.7 Hz, 1H), 6.48 (td, *J* = 7.7 Hz, 1H), 6.06 (d, *J* = 7.7 Hz, 1H), 5.27 – 5.15 (m, 1H), 4.46 (dd, *J* = 11.6, 3.5 Hz, 1H), 4.40 (dd, *J* = 11.5, 1.7 Hz), 3.33 (s, 3H), 1.25 (d, *J* = 7.0 Hz, 3H). **^{13}C NMR** (101 MHz, $CDCl_3$) δ 168.3, 155.2, 149.1, 142.4, 133.1, 130.3, 128.9, 128.7, 128.3, 127.5, 125.1, 124.6, 123.9, 122.4, 121.3, 118.4, 112.9, 107.4, 77.4, 73.0, 29.4, 26.0, 16.3.

Analytical data are in agreement with the literature.[3]

2-Methyl-3-(naphthalen-2-ylthio)propanoic acid 20

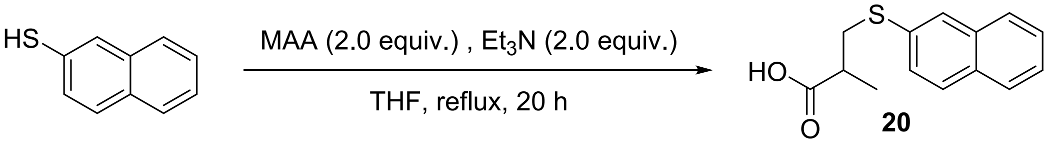

To a solution of naphthalen-2-thiol (1.00 g, 6.2 mmol) in THF (30 mL) was added triethylamine (1.74 mL, 12.5 mmol) and methacrylic acid (1.00 mL, 12.5 mmol). The mixture was heated to reflux and stirred at this temperature for 20 h. After this time, the reaction mixture was cooled to 0 °C and quenched by addition of 1 M HCl (30 mL). The organic and aqueous layers were separated and the aqueous layer was extracted with EtOAc (3 × 30 mL). The combined organic phases were washed with brine (30 mL), dried with $MgSO_4$, filtered and concentrated under reduced pressure. The crude product was purified by recrystallization from hexane to yield 3-((naphthalen-2-yl)thio)-2-methylpropanoic acid **20** (1.30 g, 5.1 mmol, 82%) as a white solid.

mp: 87.3 – 90.1 °C. **^{1}H NMR** (400 MHz, $CDCl_3$) δ 7.84–7.73 (m, 4H), 7.52–7.41 (m, 3H), 3.39 (dd, *J* = 13.4, 6.9 Hz, 1H), 3.02 (dd, *J* = 13.4, 7.1 Hz, 1H), 2.76 (sex, *J* = 7.0 Hz, 1H), 1.33 (d, *J* = 7.0 Hz, 3H). Carboxylic acidic proton was not observed due to the fast exchange time. **^{13}C NMR** (101 MHz, $CDCl_3$) δ 180.9, 133.9, 133.0, 132.2, 128.8, 128.5, 128.1, 127.9, 127.4, 126.8, 126.1, 39.7, 37.1, 16.7.

Analytical data are in agreement with the literature.[6]

2-Methyl-3-(naphthalen-2-ylthio)propanoyl chloride 21

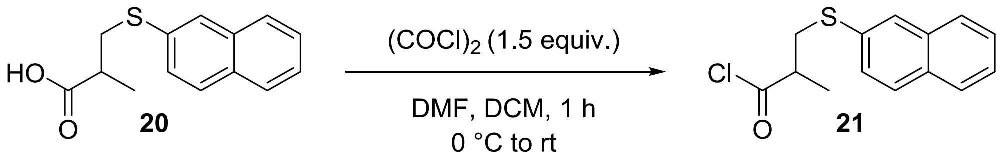

A solution of 2-methyl-3-(naphthalen-2-ylthio)propanoic acid **20** (1.00 g, 4.1 mmol) in DCM (30 mL) was cooled to 0 °C and treated with a drop of DMF followed by the dropwise addition of oxalyl chloride (0.51 mL, 6.1 mmol). The mixture was allowed to warm to rt and stirred for 1 h. After this time, the solvent and excess oxalyl chloride were removed under reduced pressure to provide the crude product 2-methyl-3-(naphthalen-2-ylthio)propanoyl chloride **21** as a brown oil. The crude product was carried forward without purification.

2-Methyl-2,3-dihydro-1H-benzo[*f*]thiochromen-1-one 22

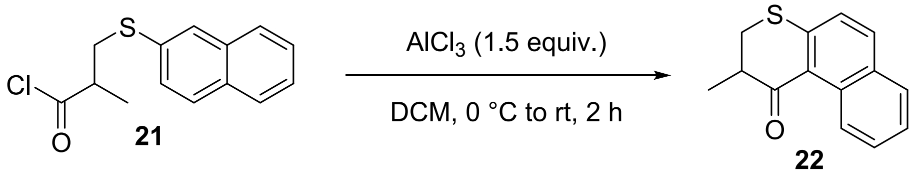

To a solution of crude 2-methyl-3-(naphthalen-2-ylthio)propanoyl chloride **21** (4.1 mmol) in DCM (30 mL) at 0 °C was carefully added $AlCl_3$ (0.816 g, 6.08 mmol) portion wise. After the addition, the mixture was allowed to warm to rt and stirred for 2 h. The mixture was then re-cooled to 0 °C and the reaction was carefully quenched with H_2O (30 mL). The organic phase was separated, and the aqueous phase was extracted with DCM (3 x 30 mL). The combined organic phases were combined, washed with $NaHCO_{3(aq)}$ (50 mL), brine (50 mL), dried over Mg_2SO_4, filtered and concentrated under reduced pressure. The crude product was purified by column chromatography (SiO_2, hexane:EtOAc, 10:1) to yield 2-methyl-2,3-dihydro-1H-benzo[*f*]thiochromen-1-one **22** (0.88 g, 3.9 mmol, 95% over two steps) as an orange oil.

^{1}H NMR (400 MHz, $CDCl_3$) δ 9.08 (d, *J* = 8.8 Hz, 1H), 7.74 (t, *J* = 8.7 Hz, 2H), 7.58 (ddd, *J* = 8.6, 6.9, 1.5 Hz, 1H), 7.43 (ddd, *J* = 8.1, 6.9, 1.1 Hz, 1H), 7.23 (d, *J* = 8.8 Hz, 1H), 3.28 – 3.04 (m, 3H), 1.39 (d, *J* = 6.5 Hz, 3H). **^{13}C NMR** (101 MHz, $CDCl_3$) δ 199.4, 144.1, 133.4, 132.6, 131.8, 129.1, 128.5, 125.8, 125.6, 125.43, 125.2, 43.0, 32.9, 15.4.

Analytical data are in agreement with the literature.[6]

(*E*)-1-methyl-3-(2-methyl-2,3-dihydro-1H-benzo[f]thiochromen-1-ylidene)indolin-2-one 4

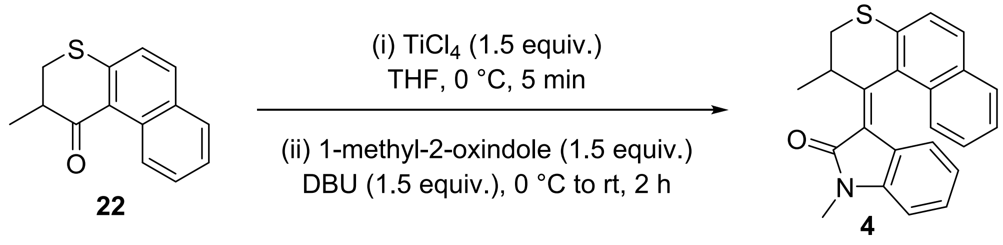

To a solution of 2-methyl-2,3-dihydro-1H-benzo[*f*]thiochromen-1-one **22** (0.114 g, 0.50 mmol) in THF (3 mL) at 0 °C was added titanium(IV) tetrachloride (0.75 mL, 1 M in toluene, 0.75 mmol) dropwise and the resulting solution was stirred for 5 minutes, after which the solution had turned dark orange. A solution of 1-methyl-2-oxindole (0.110 g, 0.75 mmol) in THF (3 mL) was then added dropwise to the reaction mixture at 0 °C, followed by 1,8-diazabicyclo[5.4.0]undec-7-ene (DBU) (112 µL, 0.75 mmol), after which a black precipitate formed. The reaction mixture was then allowed to warm to rt and stirred for a further 2 h. The reaction was then quenched with aqueous 1M HCl and the organic phase was separated and the aqueous phase was extracted with EtOAc (3 × 30 mL). The combined organic phases were washed with H_2O (30 mL), brine (30 mL), dried over $MgSO_4$, filtered and concentrated under reduced pressure. The crude mixture was purified using column chromatography (SiO_2, hexane:EtOAc , 10:1) to yield (*E*)-1-methyl-3-(2-methyl-2,3-dihydro-1H-benzo[f]thiochromen-1-ylidene)indolin-2-one **4** (0.07 g, 0.20 mmol, 39%) as a yellow solid.

mp: 151.5 – 154.7 °C. **^{1}H NMR** (400 MHz, $CDCl_3$) δ 7.84 (d, *J* = 8.2 Hz, 1H), 7.81 (d, *J* = 8.6 Hz, 1H), 7.72 (d, *J* = 8.6 Hz, 1H), 7.47 (d, *J* = 8.6 Hz, 1H), 7.39 (ddd, *J* = 8.0, 6.9, 1.1 Hz, 1H), 7.28 (ddd, *J* = 8.2, 6.9, 1.3 Hz, 1H), 7.03 (td, *J* = 7.8, 1.1 Hz, 1H), 6.71 (d, *J* = 7.7 Hz, 1H), 6.34 (td, *J* = 7.8, 1.0 Hz, 1H), 5.66 (sex, *J* = 6.7 Hz, 1H), 5.60 (d, *J* = 7.8 Hz, 1H), 3.33 (dd, *J* = 12.0, 6.7 Hz, 1H), 3.30 (s, 3H), 2.74 (dd, *J* = 12.0, 6.7 Hz, 1H), 1.16 (d, *J* = 6.7 Hz, 3H). **^{13}C NMR** (101 MHz, $CDCl_3$) δ 167.9, 153.2, 142.6, 137.3, 131.6, 131.4, 130.4, 129.3, 128.7, 128.6, 127.7, 126.9, 125.5, 124.3, 124.0, 123.4, 121.9, 121.6, 107.5, 36.0, 33.4, 26.0, 18.1.

Analytical data are in agreement with the literature.[3]

5,6-dimethoxy-2,7-dimethyl-2,3-dihydro-1H-inden-1-one 23

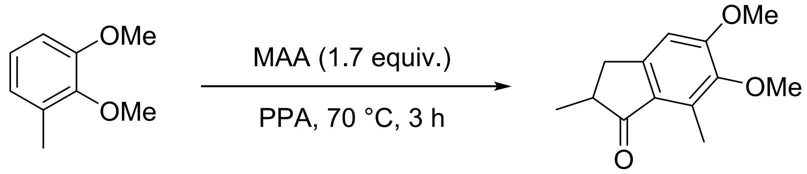

To a solution of polyphosphoric acid (PPA, 115% H_3PO_4, 2 mL) at 70 °C was added methacrylic acid (0.58 mL, 6.9 mmol, 1.7 equiv.) and 2,3-dimethoxytoluene (0.60 mL, 4.1 mmol, 1.0 equiv.). The reaction mixture was stirred for 3 h and then allowed to cool to rt and quenched by the addition of ice and left to stir for a further 12 h. The reaction mixture was then extracted with EtOAc (3 × 100 mL), and the combined organic phases were washed with saturated aq. $NaHCO_3$ (50 mL), brine (50 mL), dried over MgSO4, filtered through Celite and concentrated under reduced pressure to give a light brown solid. The crude product was recrystallized from heptane to yield 5,6-dimethoxy-2,7-dimethyl-2,3-dihydro-1H-inden-1-one **23** (0.39 g, 15%) as a brown solid.

mp: 102.6 – 104.2 °C. **^{1}H NMR** (400 MHz, $CDCl_3$) δ 6.74 (s, 1H), 3.92 (s, 3H), 3.76 (s, 3H), 3.25 (dd, *J* = 16.4, 7.4 Hz, 1H), 2.69 – 2.57 (m, 2H), 2.56 (s, 3H), 1.27 (d, *J* = 7.4 Hz, 3H). **^{13}C NMR** (101 MHz, $CDCl_3$) δ 209.2, 158.2, 152.0, 147.0, 132.3, 127.4, 106.0, 60.6, 56.0, 42.8, 34.7, 16.8, 10.8.

Analytical data are in agreement with the literature.[7]

(E)-3-(5,6-dimethoxy-2,7-dimethyl-2,3-dihydro-1H-inden-1-ylidene)-1-methylindolin-2-one 5

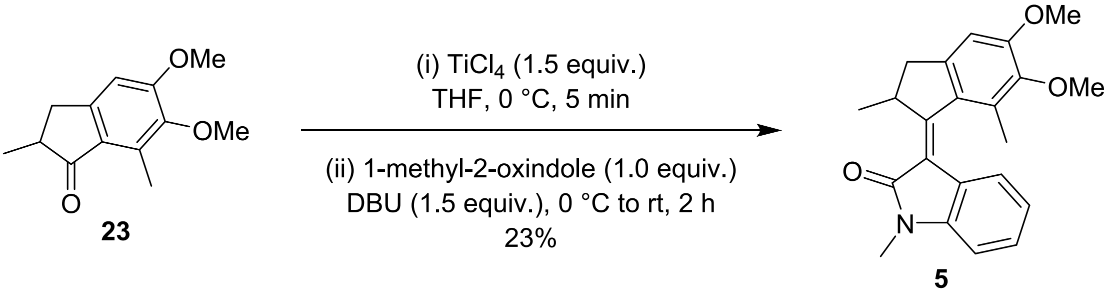

To a solution of 5,6-dimethoxy-2,7-dimethyl-2,3-dihydro-1H-inden-1-one **23** (0.22 g, 1.0 mmol) in THF (4.5 mL) at 0 °C was added titanium(IV) tetrachloride (1.5 mL, 1 M in toluene, 1.5 mmol) dropwise and the resulting solution was stirred for 5 minutes, after which the solution had turned dark orange. A solution of 1-methyl-2-oxindole (0.22 g, 1.0 mmol) in THF (3 mL) was then added dropwise to the reaction mixture at 0 °C, followed by 1,8-diazabicyclo[5.4.0]undec-7-ene (DBU) (0.22 mL, 1.5 mmol), after which a black precipitate formed. The reaction mixture was then allowed to warm to rt and stirred for a further 2 h. The reaction was then quenched with aqueous 1M HCl and the organic phase was separated and the aqueous phase was extracted with EtOAc (3 × 30 mL). The combined organic phases were washed with H_2O (30 mL), brine (30 mL), dried over $MgSO_4$, filtered and concentrated under reduced pressure. The crude mixture was purified using flash column chromatography (hexane:EtOAc, 10:1) to yield (*E*)-1-methyl-3-(2-methyl-2,3-dihydro-1H-benzo[f]thiochromen-1-ylidene)indolin-2-one **5** (0.080 g, 23%) as a yellow solid.

mp 177.0 – 178.1 °C. **^{1}H NMR** (500 MHz, $CDCl_3$) δ 7.20 (td, *J* = 7.6, 1.1 Hz, 1H), 7.02 (d, *J* = 7.6 Hz, 1H), 6.89 (td, *J* = 7.6, 1.1 Hz, 1H), 6.86 (s, 1H), 6.82 (d, *J* = 7.6 Hz, 1H), 4.39 (m, 1H), 3.95 (s, 3H), 3.86 (s, 3H), 3.30 (s, 3H), 3.23 (dd, *J* = 15.1, 6.2 Hz, 1H), 2.55 (d, *J* = 15.1 Hz, 1H), 2.18 (s, 3H), 1.23 (d, *J* = 6.8 Hz, 3H). **^{13}C NMR** (126 MHz, $CDCl_3$) δ 168.8, 163.4, 155.3, 147.1, 146.0, 142.3, 132.1, 131.7, 127.6, 123.0, 122.3, 121.0, 118.5, 107.5, 107.1, 60.3, 56.0, 43.6, 41.3, 25.9, 19.3, 16.1.

Analytical data are in agreement with the literature.[7]

S3 NMR Spectra

2-Methyl-3-(naphthalen-2-yl)propanoic acid 13; ^{1}H NMR, 400 MHz, $CDCl_3$

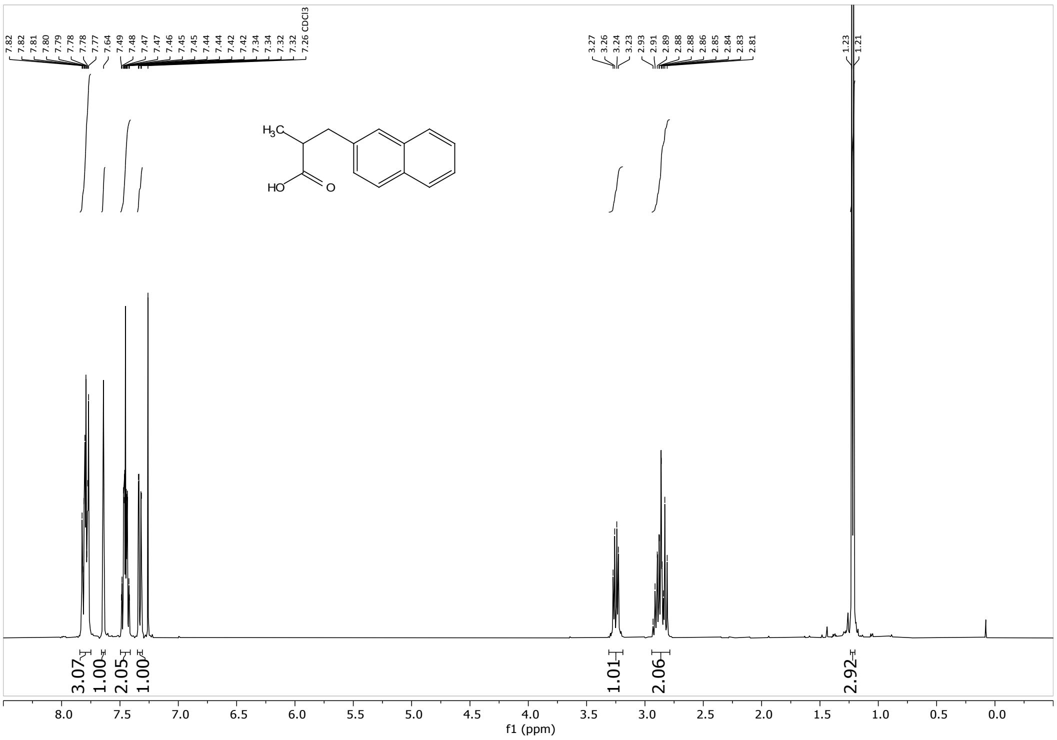

2-Methyl-3-(naphthalen-2-yl)propanoic acid 13; ^{13}C NMR, 101 MHz, $CDCl_3$

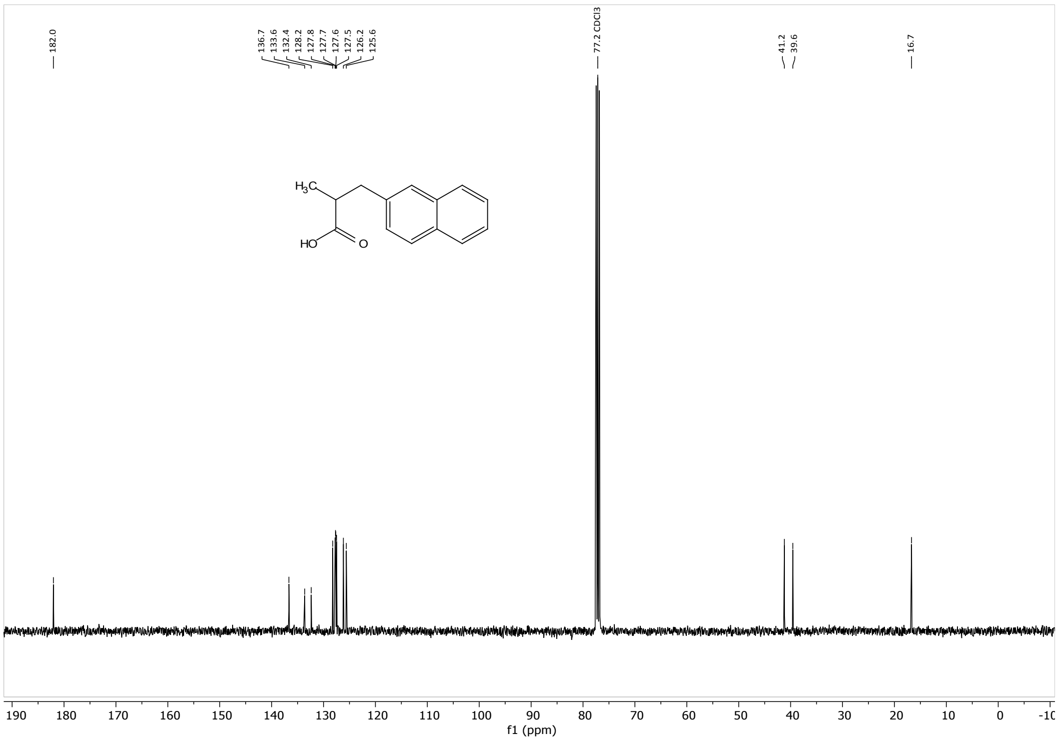

2-Methyl-2,3-dihydro-1*H*-cyclopenta[*α*]naphthalen-1-one 15; ^{1}H NMR, 400 MHz, $CDCl_3$

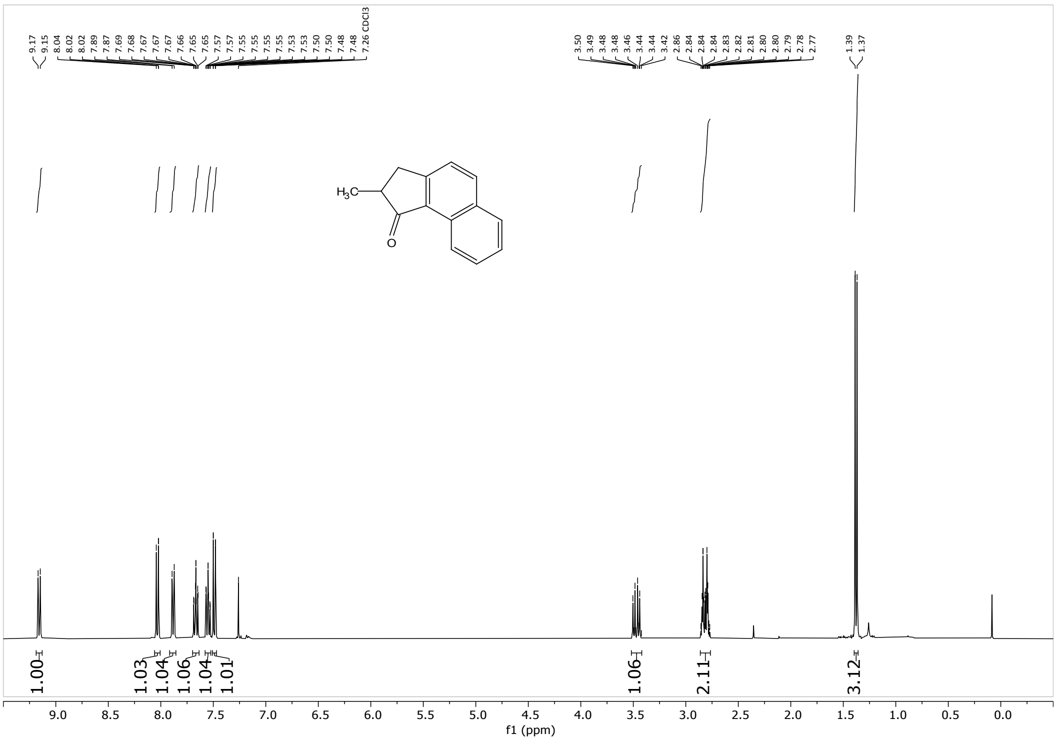

2-Methyl-2,3-dihydro-1*H*-cyclopenta[*α*]naphthalen-1-one 15; ^{13}C NMR, 101 MHz, $CDCl_3$

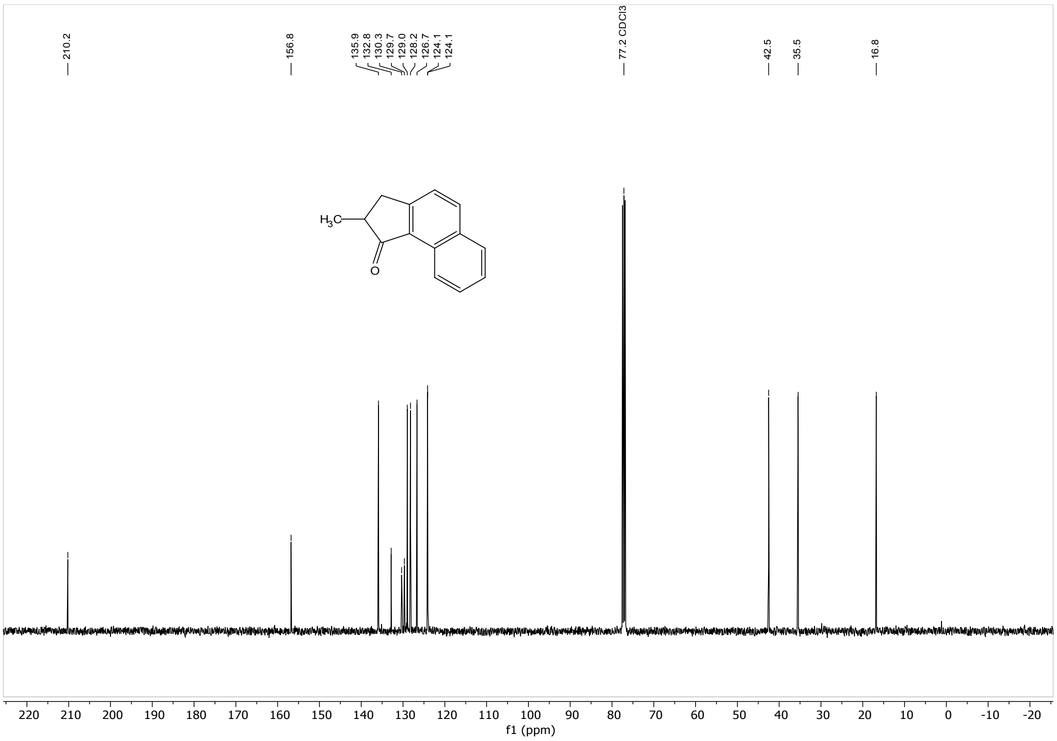

(*E*)-1-methyl-3-(2-methyl-2,3-dihydro-1H-cyclopenta[α]naphthalen-1-ylidene)indolin-2-one 1; ^{1}H NMR, 500 MHz, $CDCl_3$

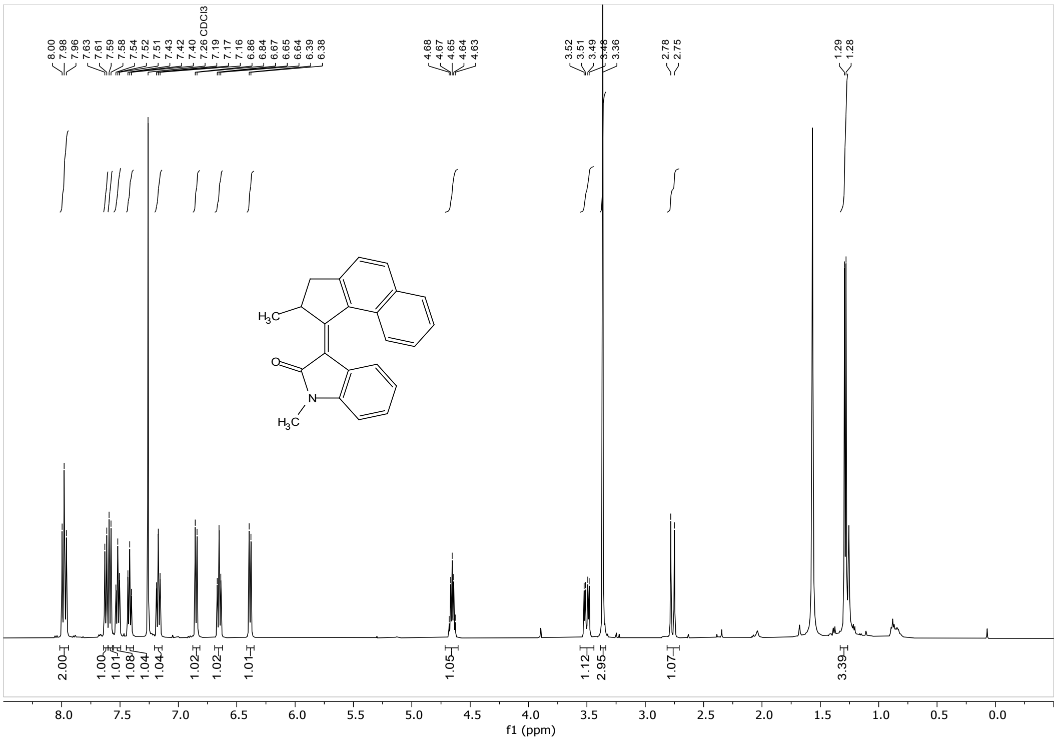

(*E*)-1-methyl-3-(2-methyl-2,3-dihydro-1H-cyclopenta[α]naphthalen-1-ylidene)indolin-2-one 1; ^{13}C NMR, 126 MHz, $CDCl_3$

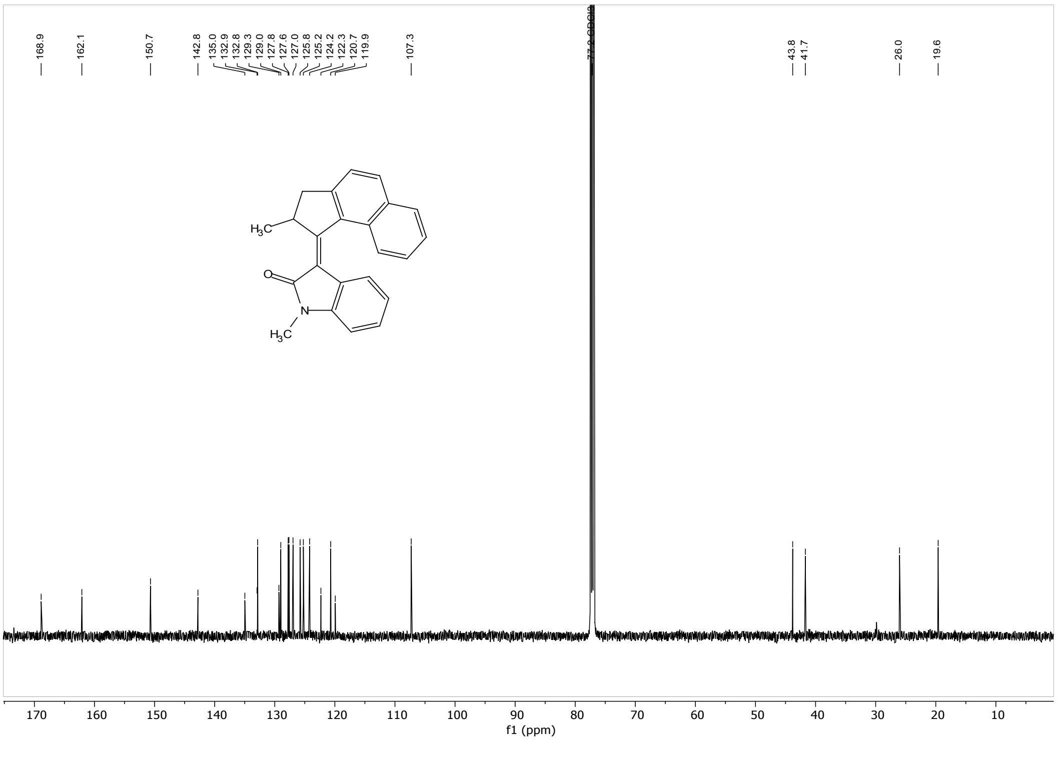

3-Methyl-2,3-dihydrophenanthren-4(1H)-one 16; ^{1}H NMR, 400 MHz, $CDCl_3$

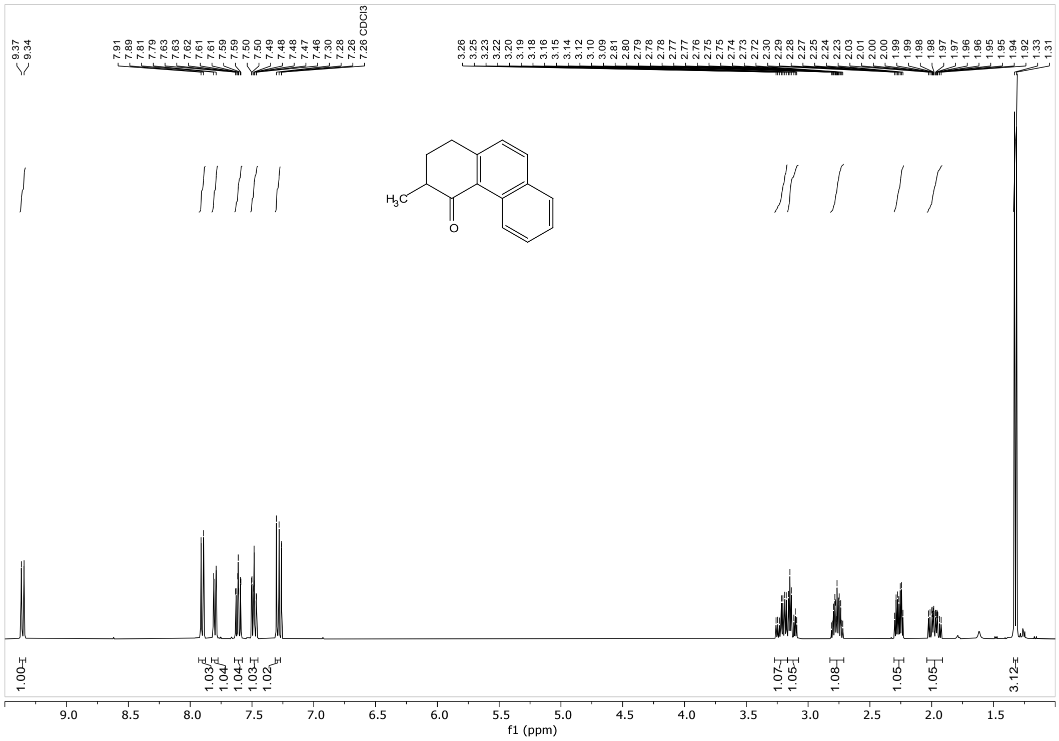

3-Methyl-2,3-dihydrophenanthren-4(1H)-one 16; ^{13}C NMR, 101 MHz, $CDCl_3$

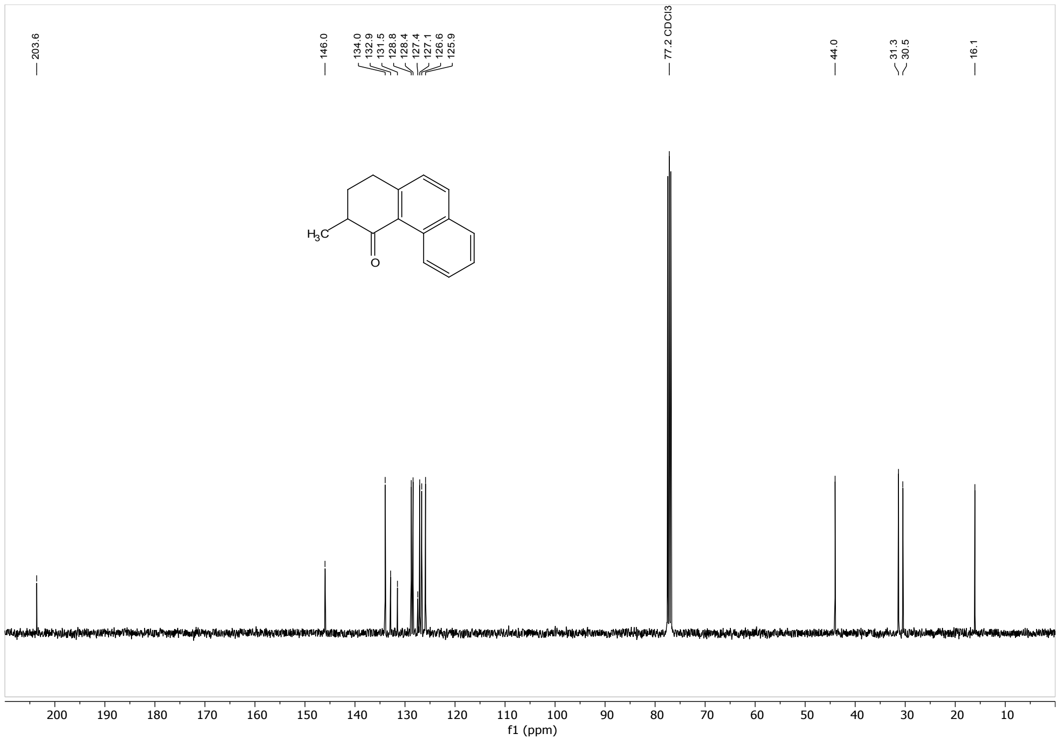

(*E*)-1-methyl-3-(3-methyl-2,3-dihydrophenanthren-4(1H)-ylidene)indolin-2-one 2; ^{1}H NMR, 400 MHz, $CDCl_3$

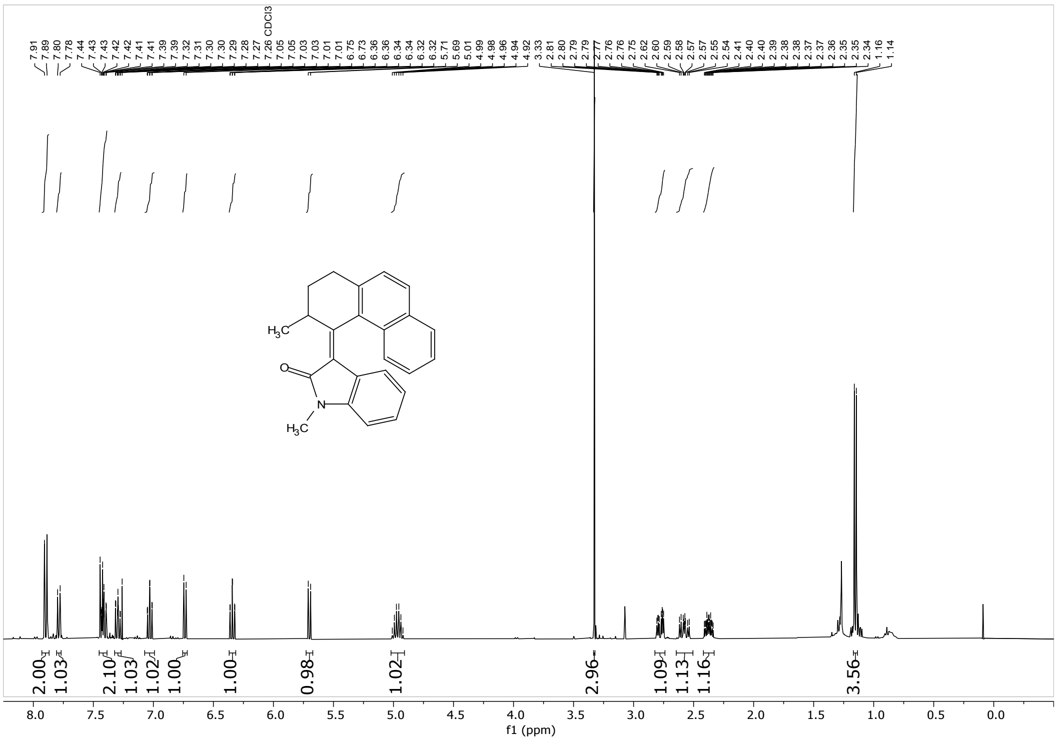

(*E*)-1-methyl-3-(3-methyl-2,3-dihydrophenanthren-4(1H)-ylidene)indolin-2-one 2; ^{13}C NMR, 101 MHz, $CDCl_3$

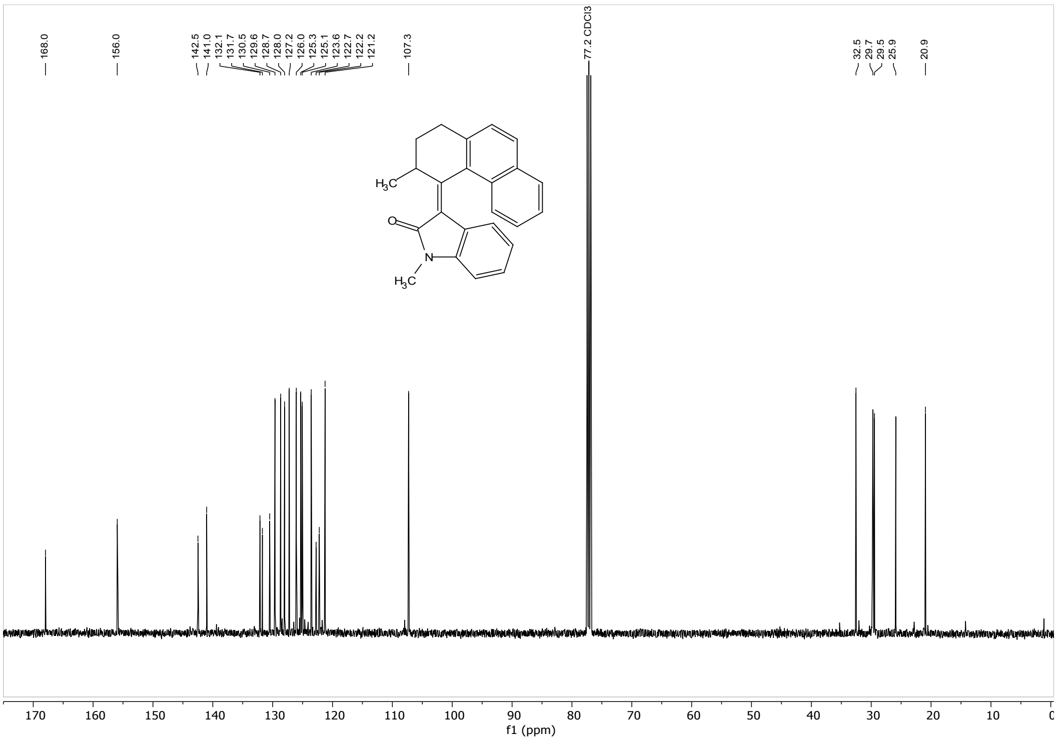

3-(Naphthalen-2-yloxy)propanenitrile 17; ^{1}H NMR, 400 MHz, $CDCl_3$

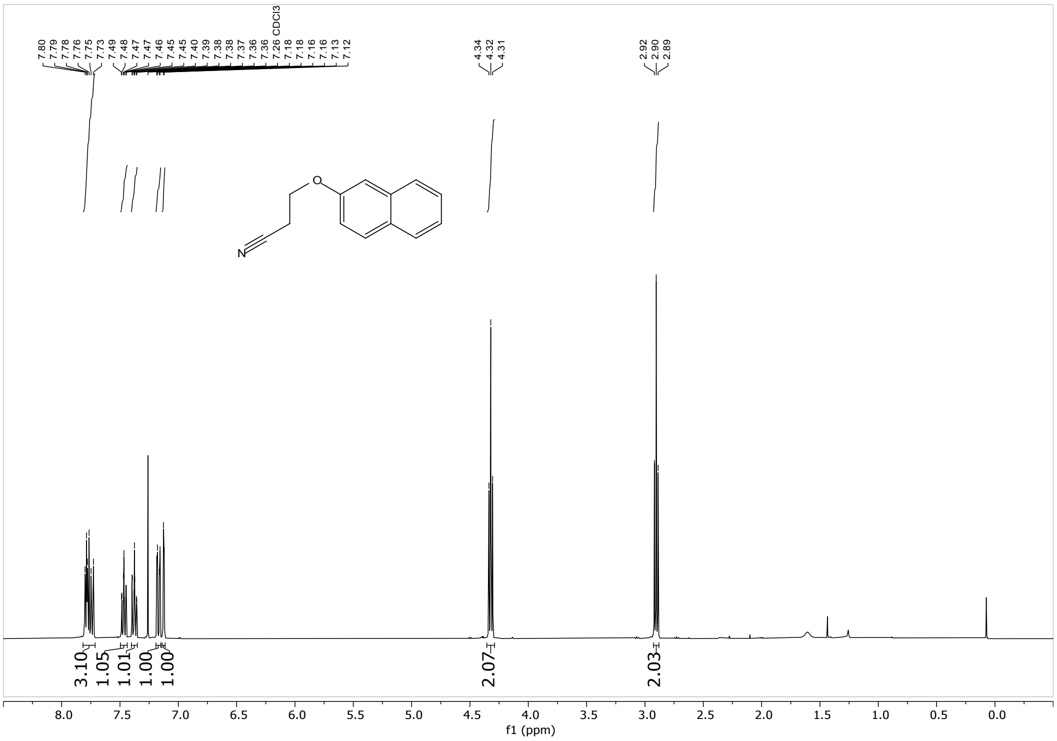

3-(Naphthalen-2-yloxy)propanenitrile 17; ^{13}C NMR, 101 MHz, $CDCl_3$

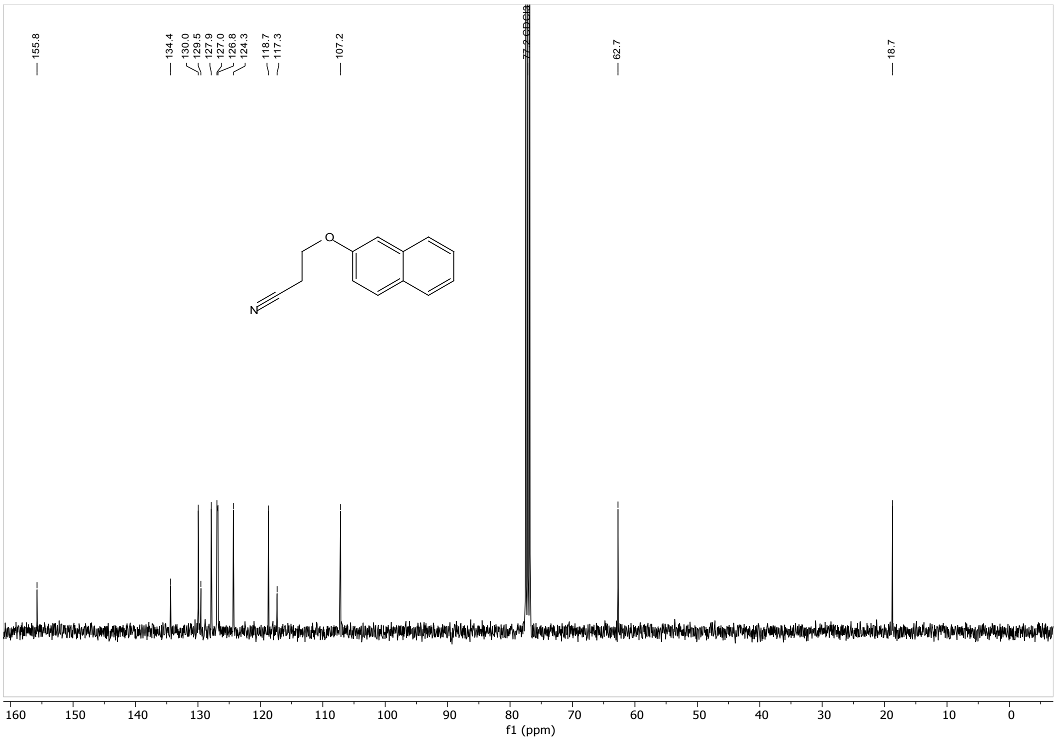

2,3-Dihydro-1*H*-benzo[*f*]chromen-1-one 18; ^{1}H NMR, 400 MHz, $CDCl_3$

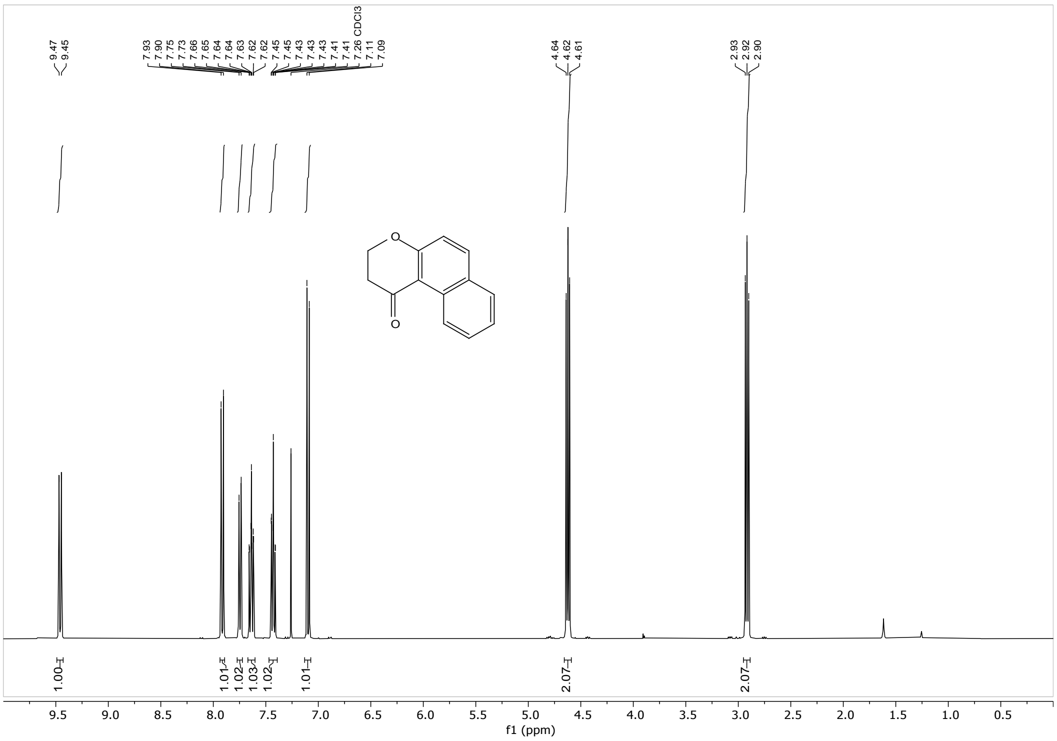

2,3-Dihydro-1*H*-benzo[*f*]chromen-1-one 18; ^{13}C NMR, 101 MHz, $CDCl_3$

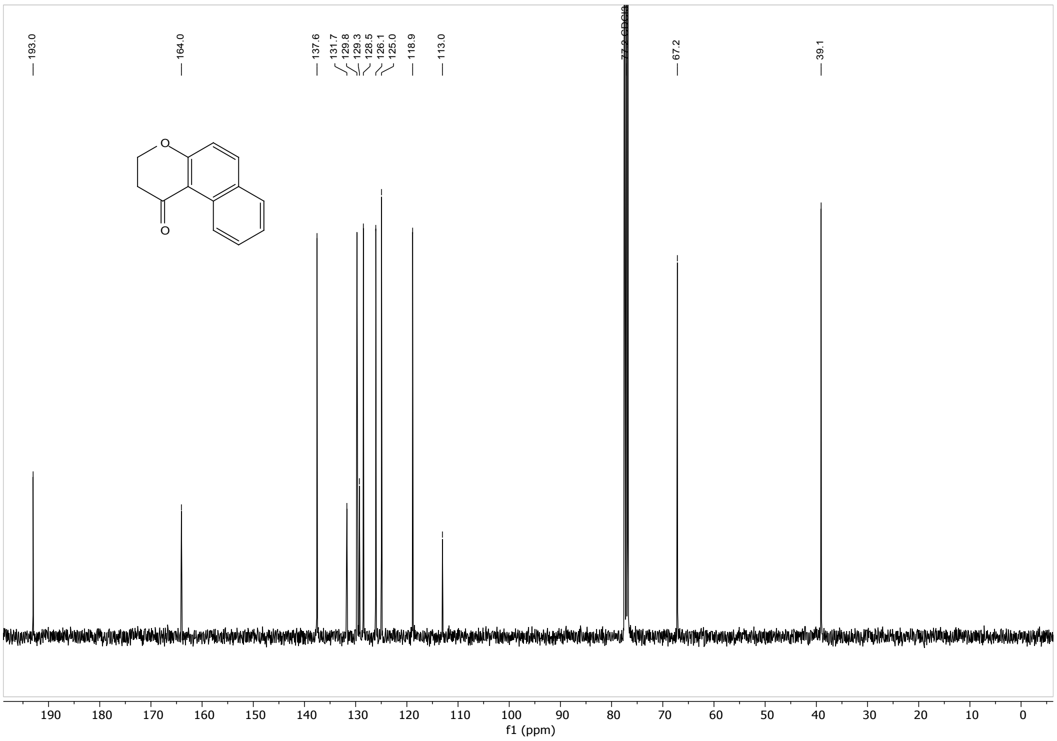

2-Methyl-2,3-dihydro-1*H*-benzo[*f*]chromen-1-one 19; ^{1}H NMR, 400 MHz, $CDCl_3$

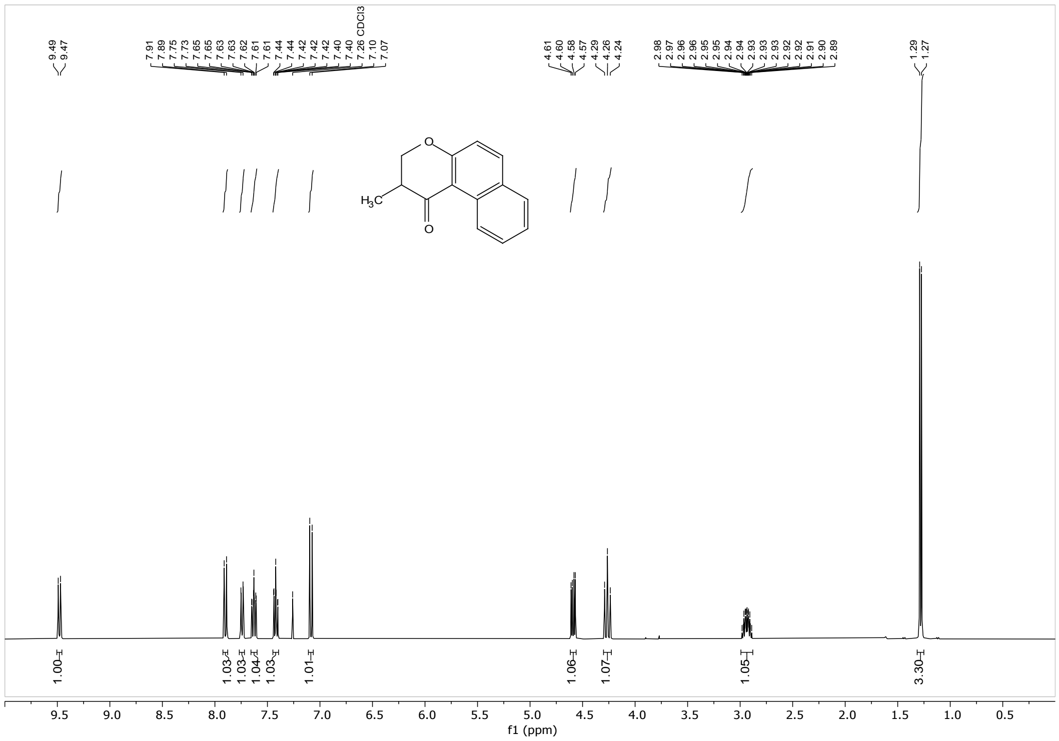

2-Methyl-2,3-dihydro-1*H*-benzo[*f*]chromen-1-one 19; ^{13}C NMR, 101 MHz, $CDCl_3$

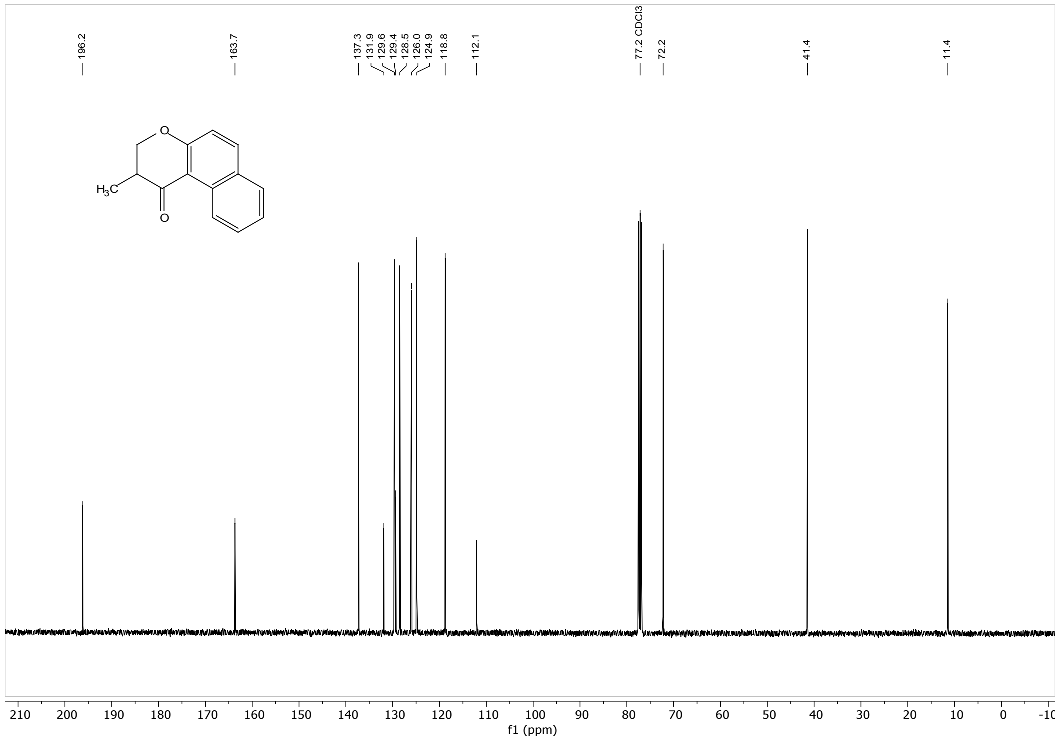

(*E*)-1-Methyl-3-(2-methyl-2,3-dihydro-1*H*-benzo[*f*]chromen-1-ylidene)indolin-2-one 3; ^{1}H NMR, 400 MHz, $CDCl_3$

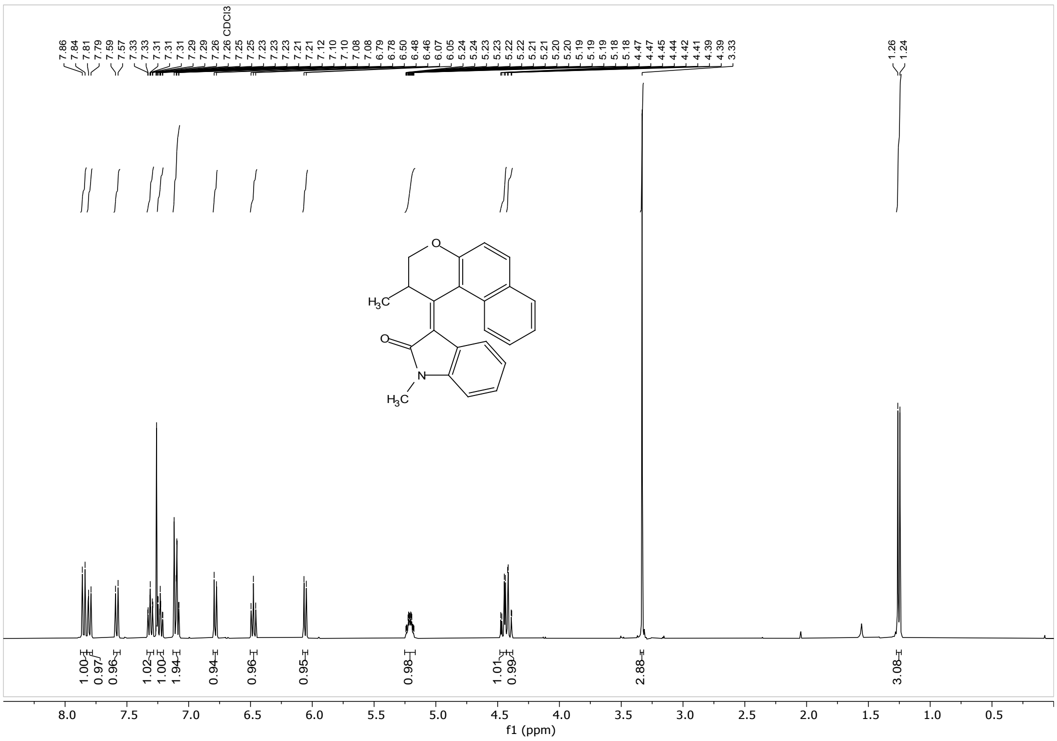

(*E*)-1-Methyl-3-(2-methyl-2,3-dihydro-1*H*-benzo[*f*]chromen-1-ylidene)indolin-2-one 3; ^{13}C NMR, 101 MHz, $CDCl_3$

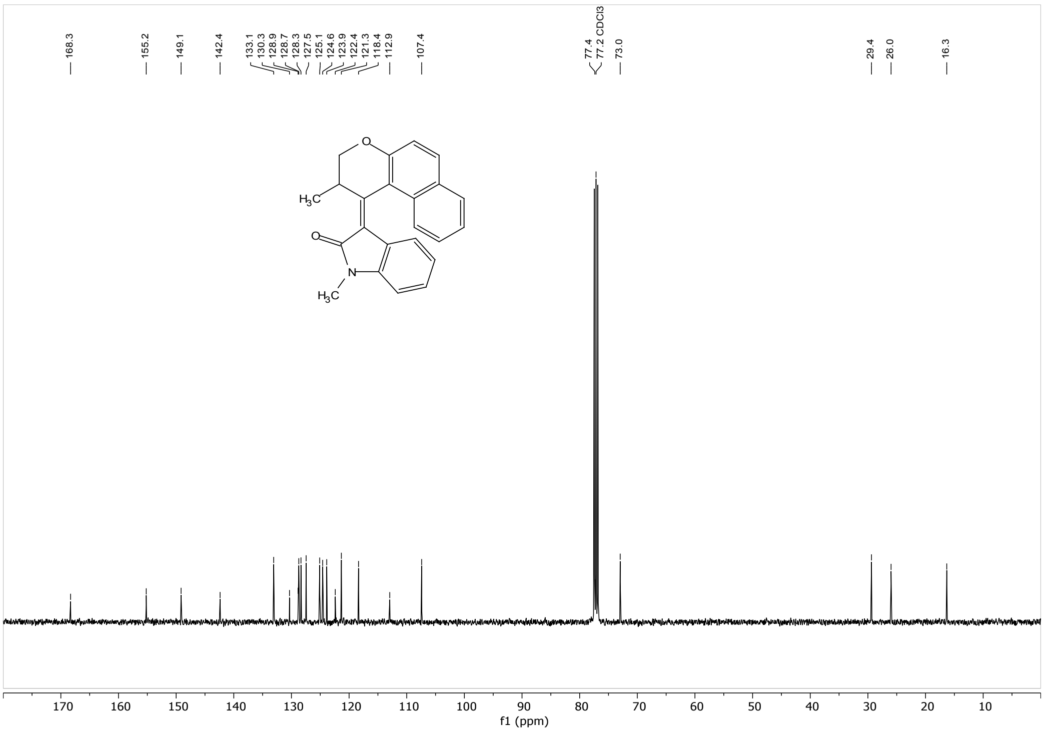

2-Methyl-3-(naphthalen-2-ylthio)propanoic acid 20; ^{1}H NMR, 400 MHz, $CDCl_3$

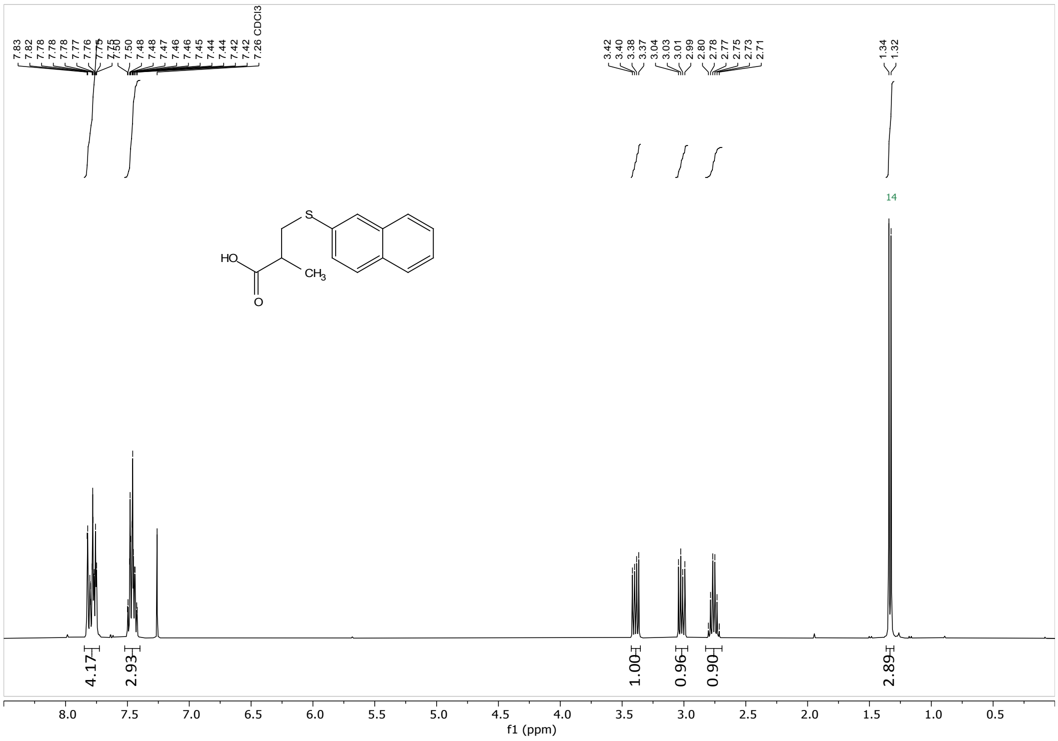

2-Methyl-3-(naphthalen-2-ylthio)propanoic acid 20; ^{13}C NMR, 101 MHz, $CDCl_3$

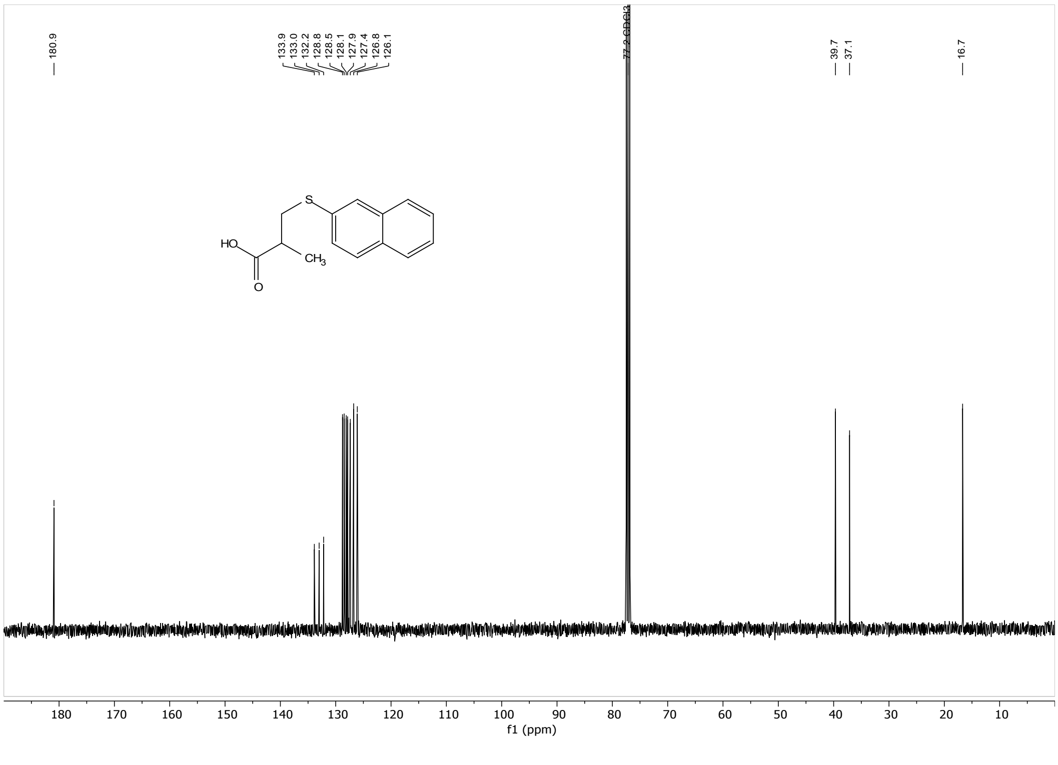

2-Methyl-2,3-dihydro-1H-benzo[*f*]thiochromen-1-one 22; ^{1}H NMR, 400 MHz, $CDCl_3$

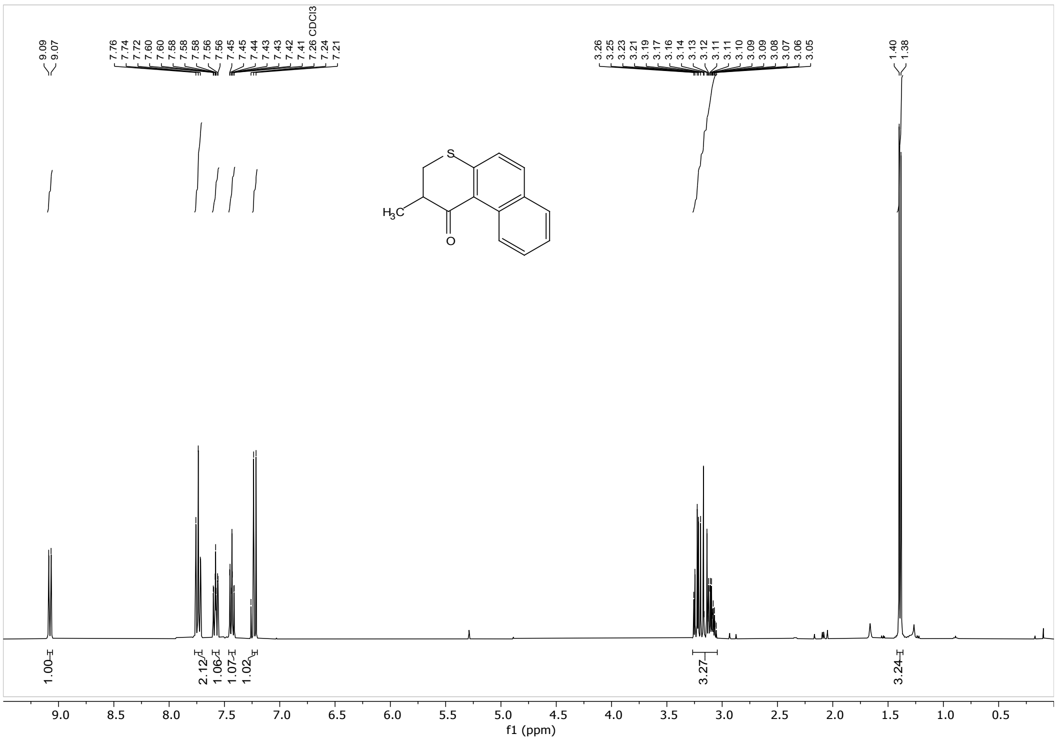

2-Methyl-2,3-dihydro-1H-benzo[*f*]thiochromen-1-one 22; ^{13}C NMR, 101 MHz, $CDCl_3$

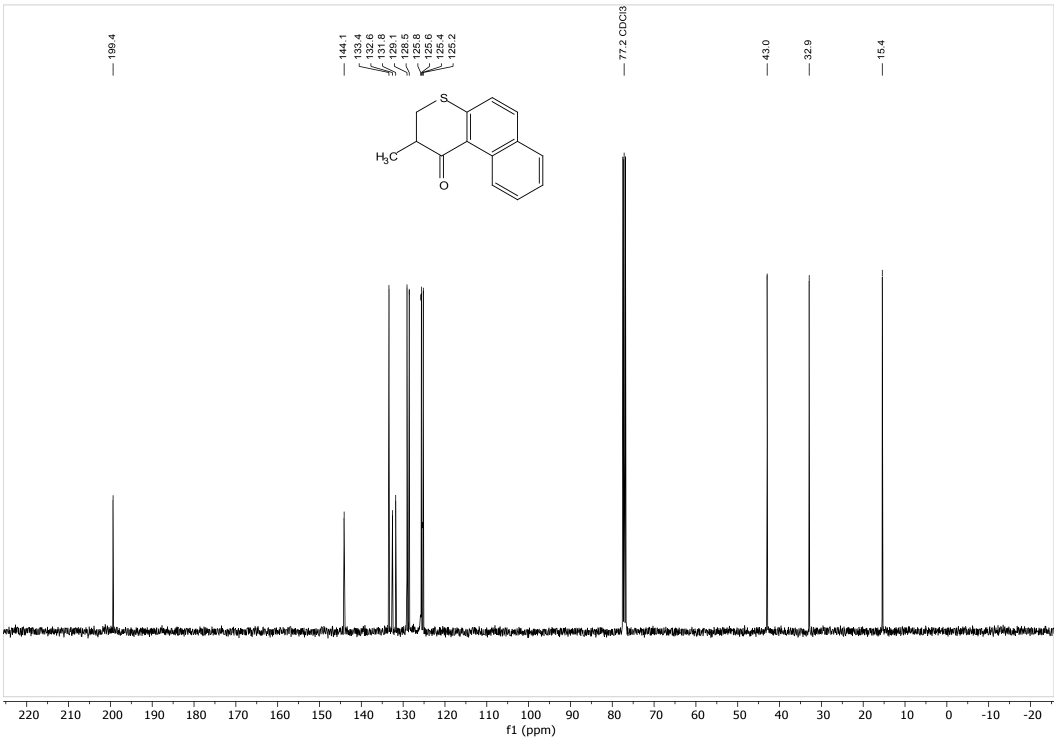

(*E*)-1-methyl-3-(2-methyl-2,3-dihydro-1H-benzo[f]thiochromen-1-ylidene)indolin-2-one 4; ^{1}H NMR, 400 MHz, $CDCl_3$

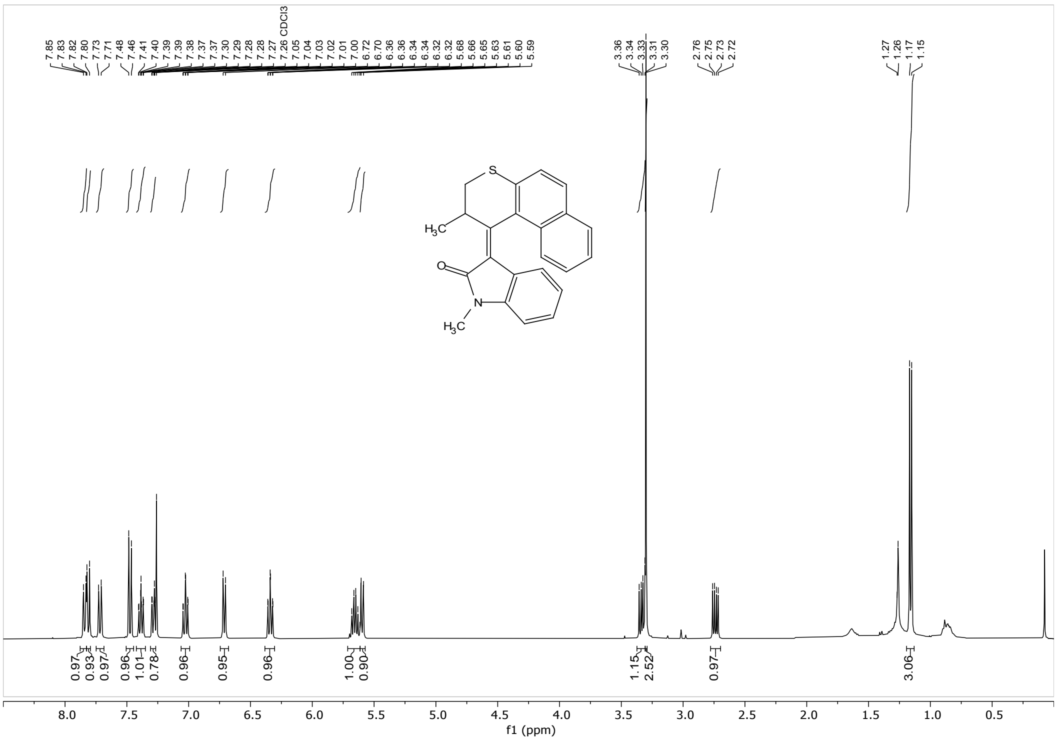

(*E*)-1-methyl-3-(2-methyl-2,3-dihydro-1H-benzo[f]thiochromen-1-ylidene)indolin-2-one 4; ^{13}C NMR, 101 MHz, $CDCl_3$

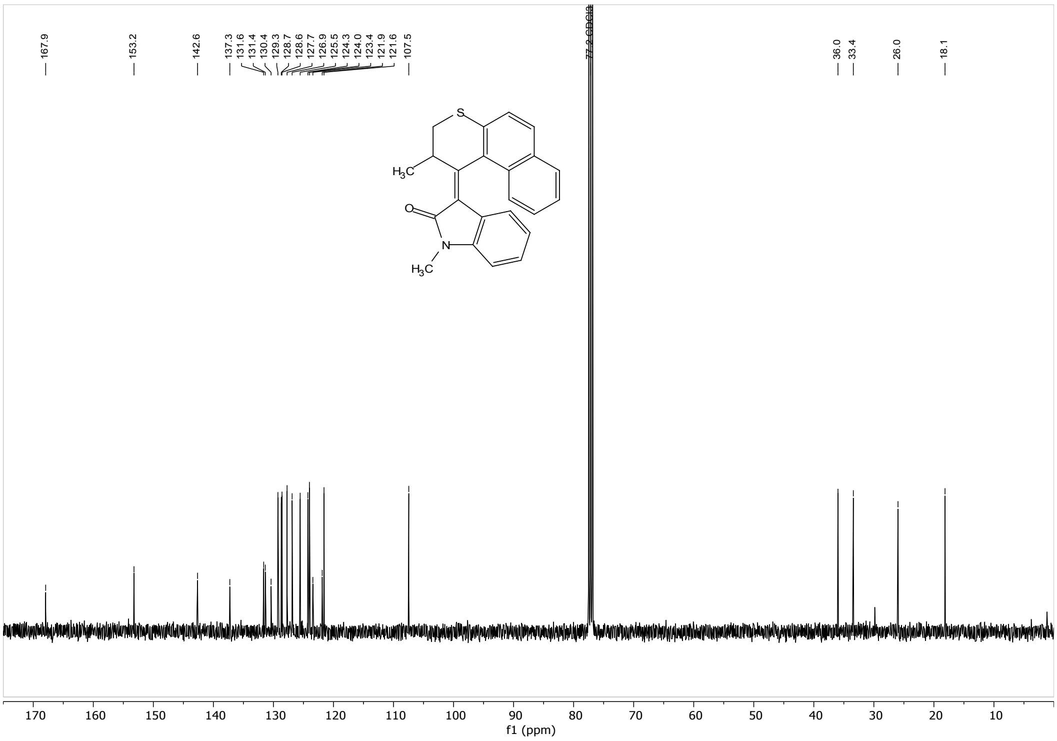

5,6-Dimethoxy-2,7-dimethyl-2,3-dihydro-1H-inden-1-one 23; ^{1}H NMR, 400 MHz, $CDCl_3$

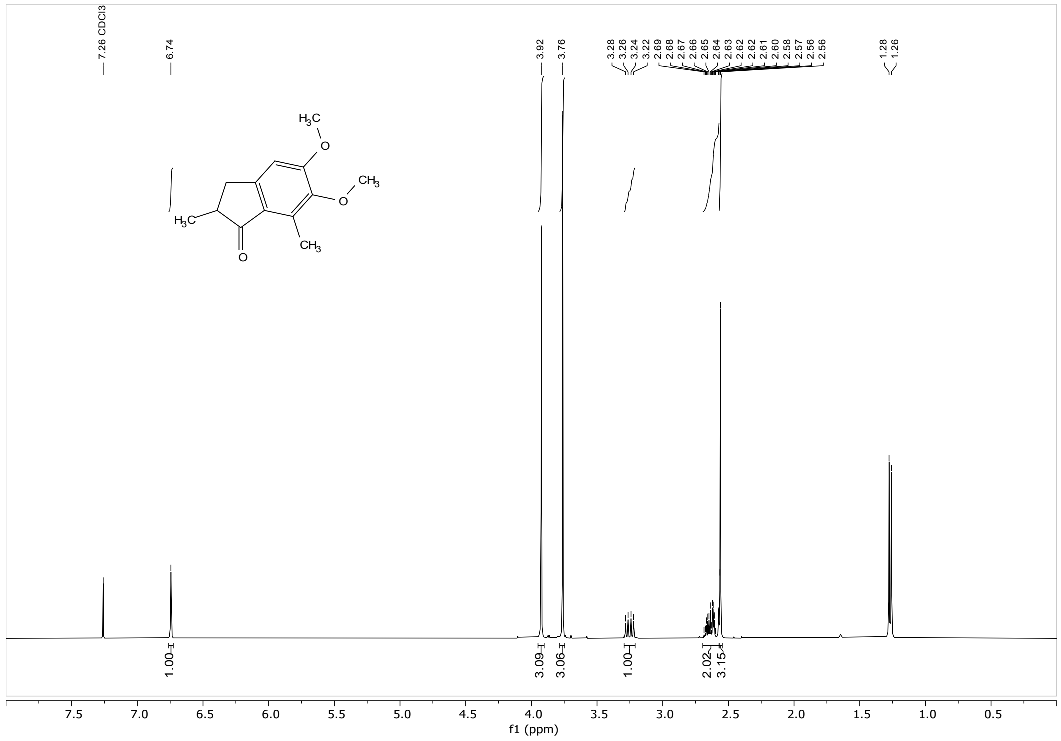

5,6-Dimethoxy-2,7-dimethyl-2,3-dihydro-1H-inden-1-one 23; ^{13}C NMR, 101 MHz, $CDCl_3$

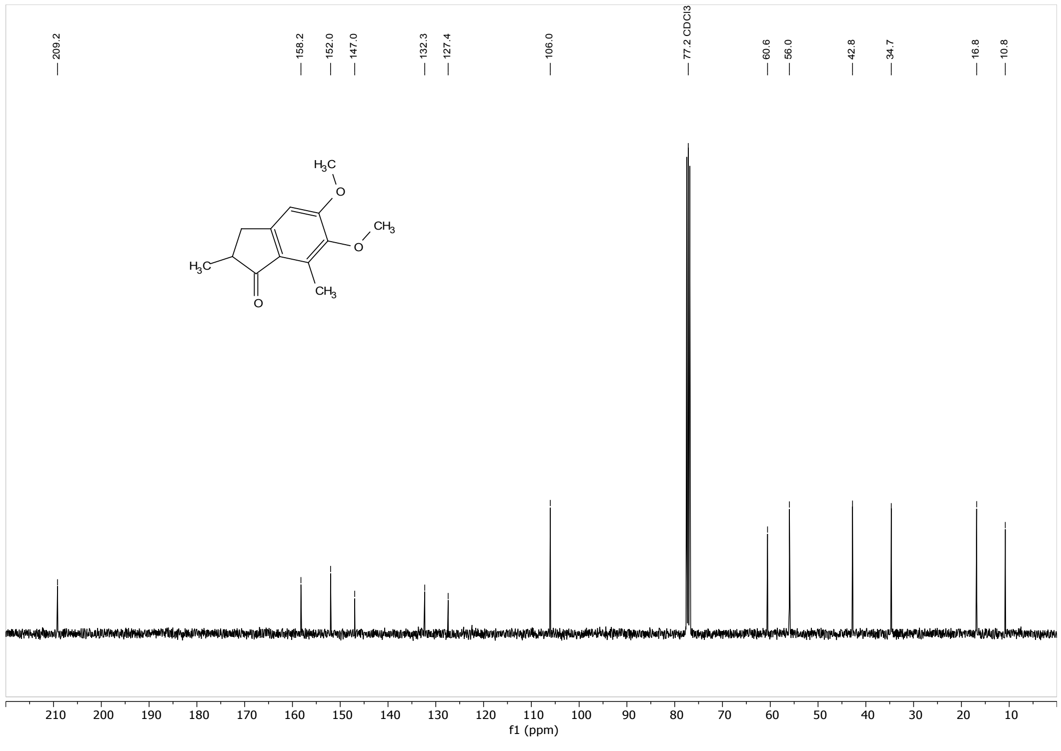

(*E*)-3-(5,6-dimethoxy-2,7-dimethyl-2,3-dihydro-1H-inden-1-ylidene)-1-methylindolin-2-one 5; ^{1}H NMR, 500 MHz, $CDCl_3$

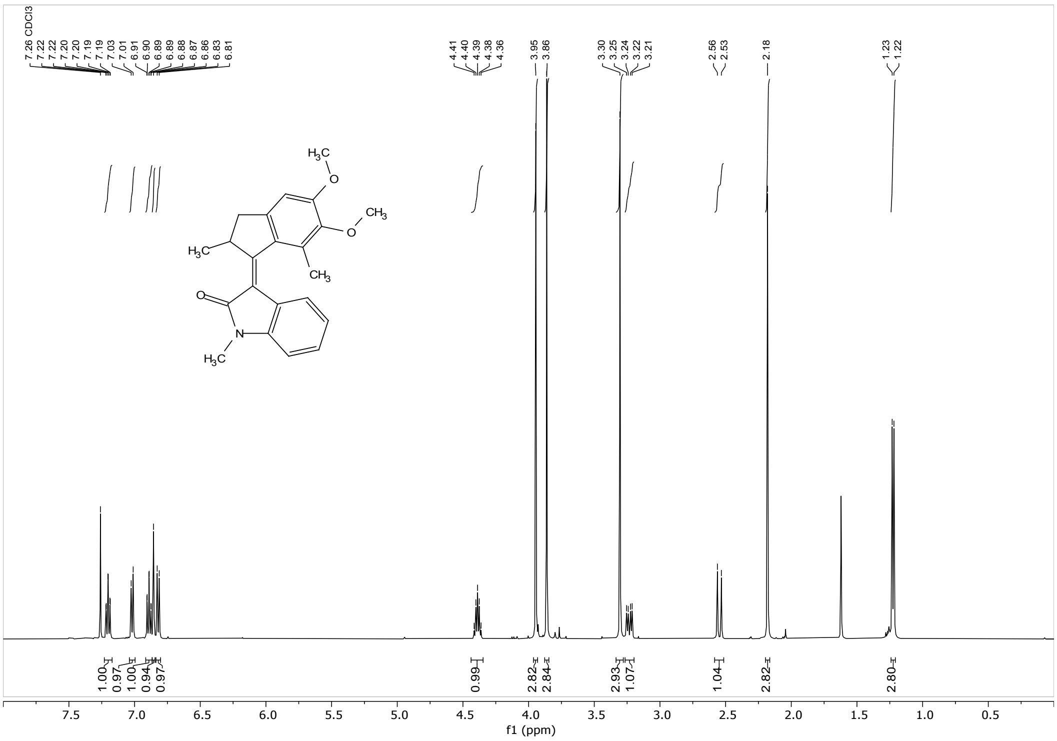

(*E*)-3-(5,6-dimethoxy-2,7-dimethyl-2,3-dihydro-1H-inden-1-ylidene)-1-methylindolin-2-one 5; ^{13}C NMR, 126 MHz, $CDCl_3$

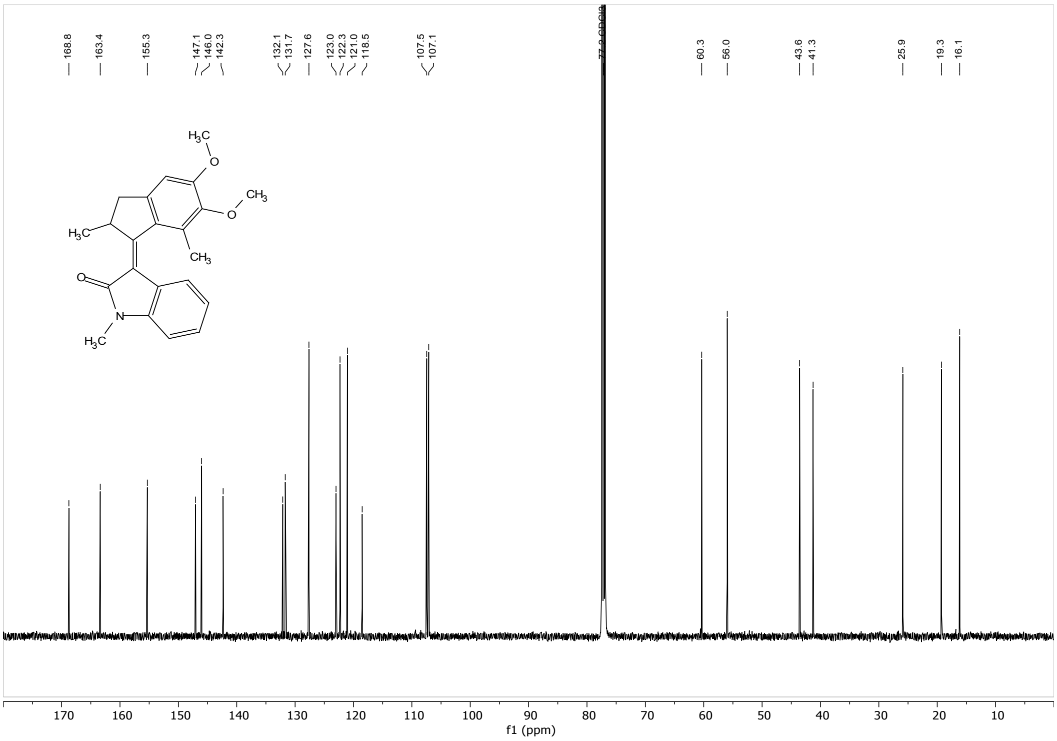

S4 Further TA and TRIR Experimental

Time-resolved spectroscopy data were processed using the Kinetics Observed After Light Absorption (KOALA) program,[9] and kinetic data were fitted using Origin 2020b software.[10] The WLC probe light was chirp-corrected using the KOALA program.[9]

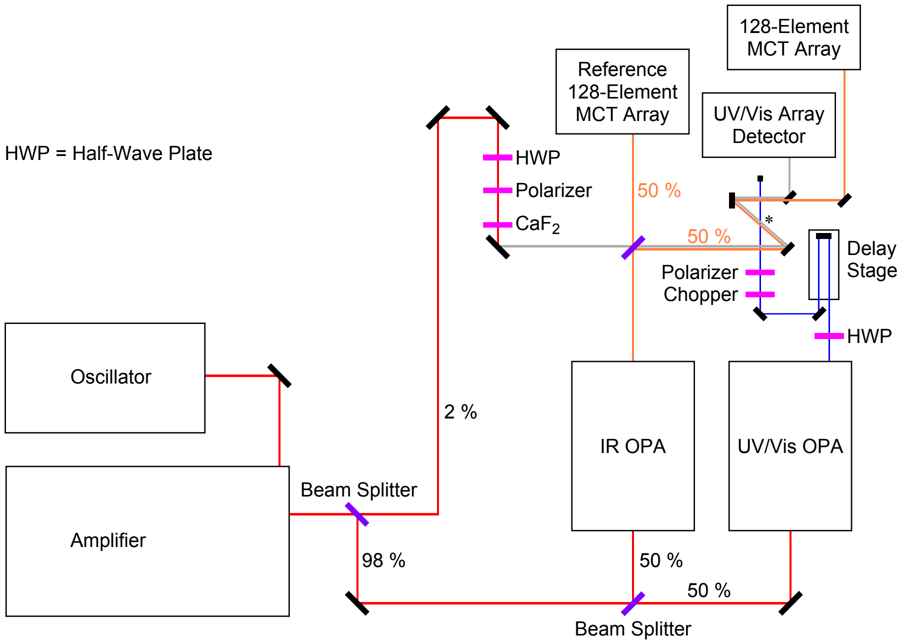

Figure S1: Diagram illustrating the layout of the TA and TRIR setups described in the main text, showing the optical paths of the: 800-nm light (red), white light continuum (grey), UV/Vis pump light (blue) and IR probe light (orange). An asterisk (*) shows the position of the sample stage.

The photochemical dynamics of *N*-methyl oxindole motors **1** – **5** were studied using TA spectroscopy in three solvents: cyclohexane, dimethyl sulfoxide (DMSO) and methanol. Deuterated cyclohexane-d_{12}, DMSO-d_6 and methanol-d_4 solvents were used for TRIR spectroscopy experiments. TA and TRIR spectra were collected following UV or visible excitation of samples continuously circulating through a stainless-steel Harrick cell fitted with CaF_2 windows sealed by Kalrez o-rings, and with window separation determined by Teflon spacers. Sample circulation used a peristaltic pump (Cole Palmer, Masterflex) and PTFE tubing. The excitation wavelengths, sample concentrations, and window spacers used are shown in Table S1. Combinations of sample concentrations and window spacings were chosen to ensure absorbances < 0.5 at the pump laser wavelength.

Table S1: Summary of the excitation wavelengths, sample concentrations and width of Teflon spacers used in the acquisition of the TA and TRIR spectra of the oxindole motors **1** – **5**.

Oxindole Motor	Excitation Wavelength / nm	Sample Concentration / mM	Window Spacer / µm
**1**	375	1.4	250
**2**	345	2.7	250
**3**	410	2.1	250
**4**	410	2.8	500
**5**	375	1.5	250

S5 Normalized UV-Vis Spectra of the Oxindole Motors

A)

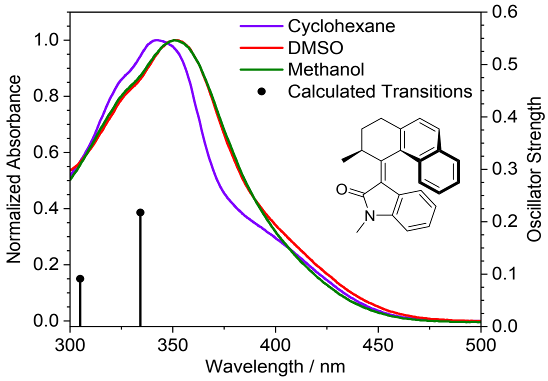

B)

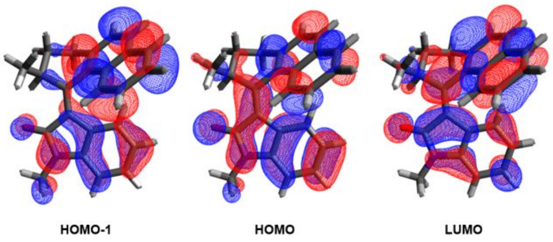

Ci)

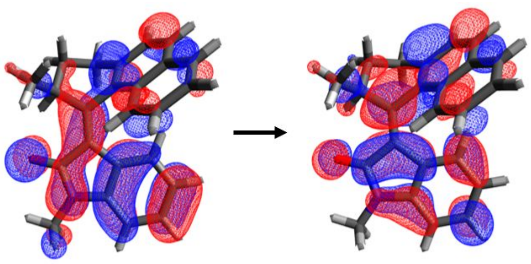

Cii)

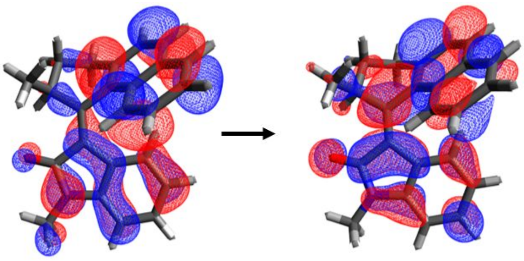

Figure S2: **A)** Normalized steady-state UV-Vis absorption spectra of motor **2** in three solvents: cyclohexane (violet), DMSO (red) and methanol (green). The vertical black lines show electronic transitions calculated using TD-DFT at the ωB97XD/6-31+G(d,p) level of theory in the gas phase. **B)** The calculated HOMO-1, HOMO and LUMO molecular orbitals. The calculated NTOs for the **Ci)** first and **Cii)** second electronic transitions for motor **2**.

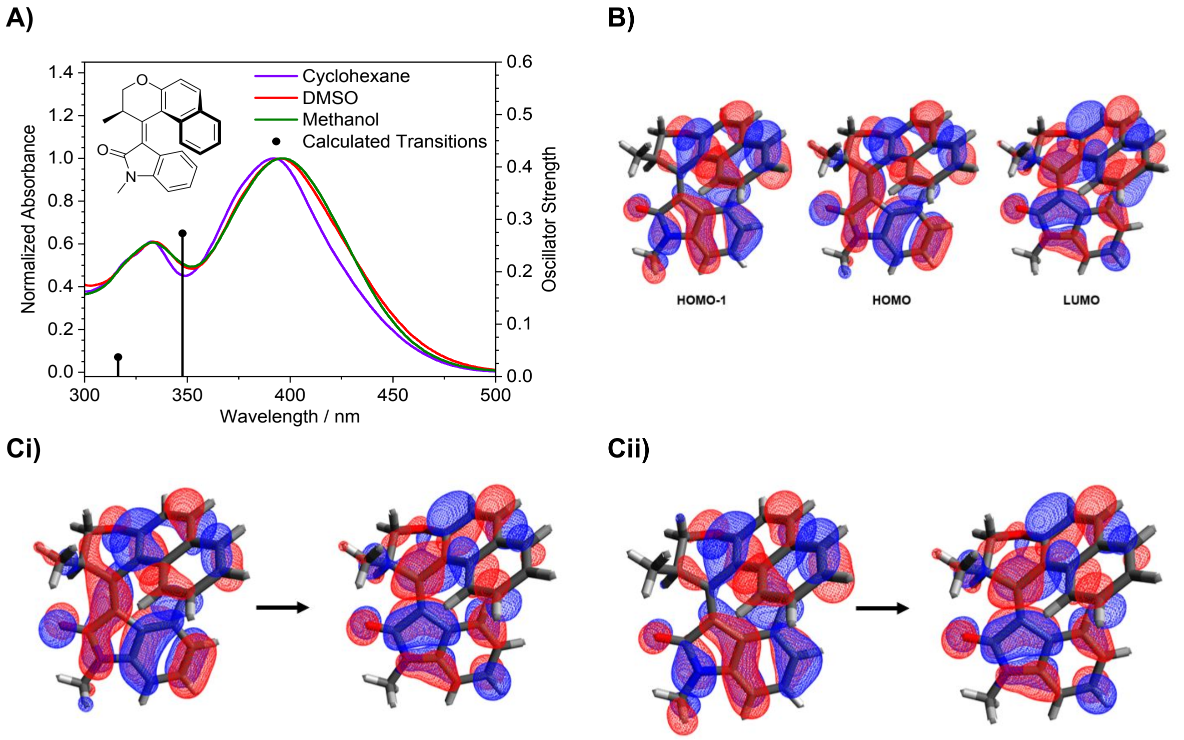

Figure S3: **A)** Normalized steady-state UV-Vis absorption spectra of motor **3** in three solvents: cyclohexane (violet), DMSO (red) and methanol (green). The vertical black lines show electronic transitions calculated using TD-DFT at the ωB97XD/6-31+G(d,p) level of theory in the gas phase. **B)** The calculated HOMO-1, HOMO and LUMO molecular orbitals. The calculated NTOs for the **Ci)** first and **Cii)** second electronic transitions for motor **3**.

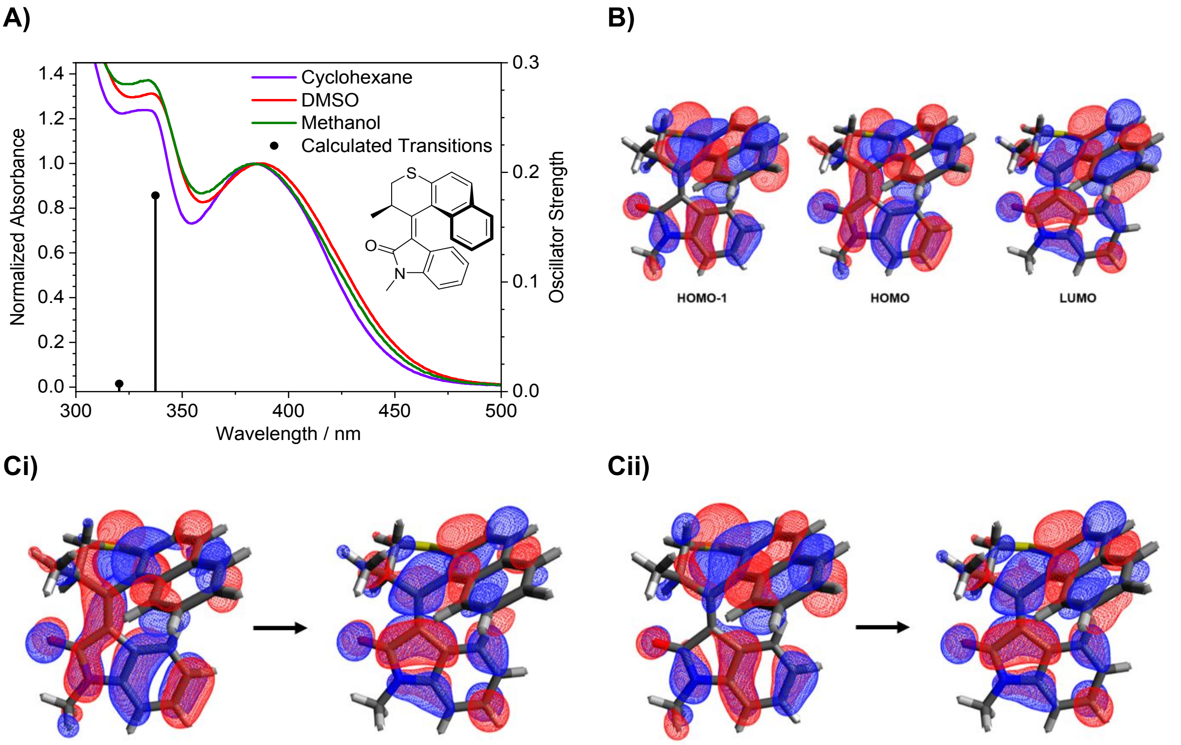

Figure S4: **A)** Normalized steady-state UV-Vis absorption spectra of motor **4** in three solvents: cyclohexane (violet), DMSO (red) and methanol (green). The vertical black lines show electronic transitions calculated using TD-DFT at the ωB97XD/6-31+G(d,p) level of theory in the gas phase. **B)** The calculated HOMO-1, HOMO and LUMO molecular orbitals. The calculated NTOs for the **Ci)** first and **Cii)** second electronic transitions for motor **4**.

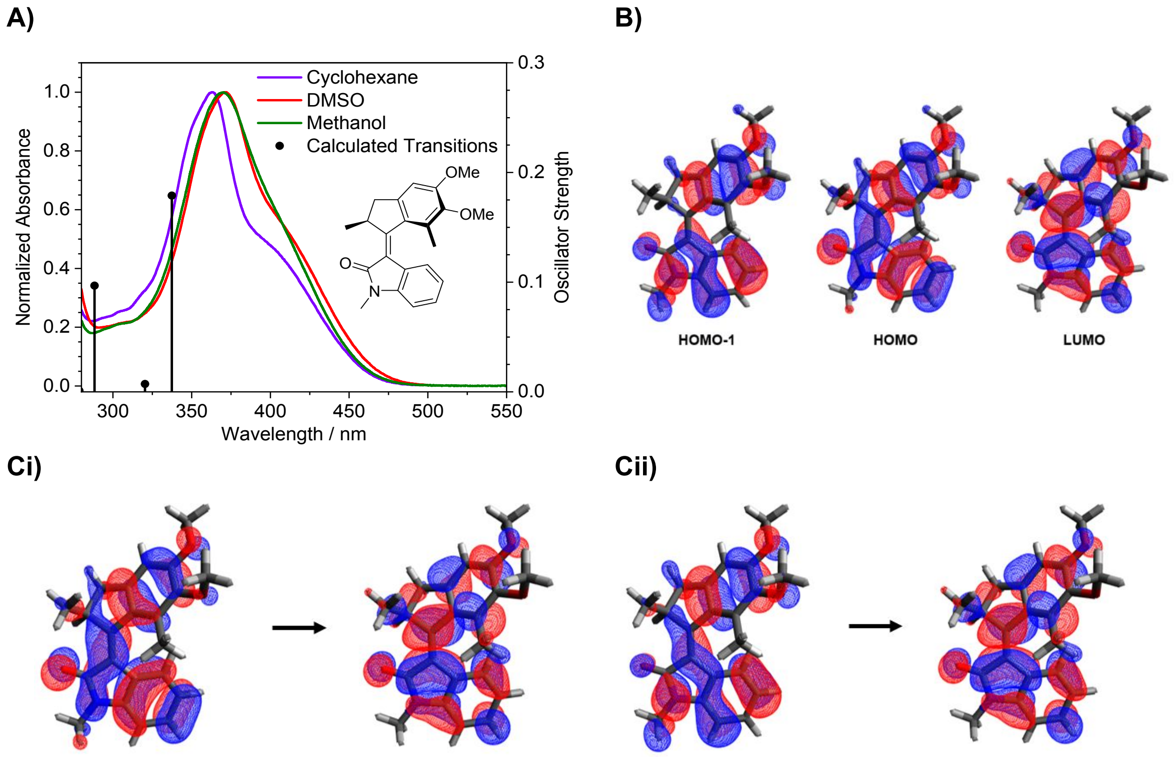

Figure S5: **A)** Normalized steady-state UV-Vis absorption spectra of motor **5** in three solvents: cyclohexane (violet), DMSO (red) and methanol (green). The vertical black lines show electronic transitions calculated using TD-DFT at the ωB97XD/6-31+G(d,p) level of theory in the gas phase. **B)** The calculated HOMO-1, HOMO and LUMO molecular orbitals. The calculated NTOs for the **Ci)** first and **Cii)** second electronic transitions for motor **5**.

S6 FTIR Spectra of the Oxindole Motors

A)

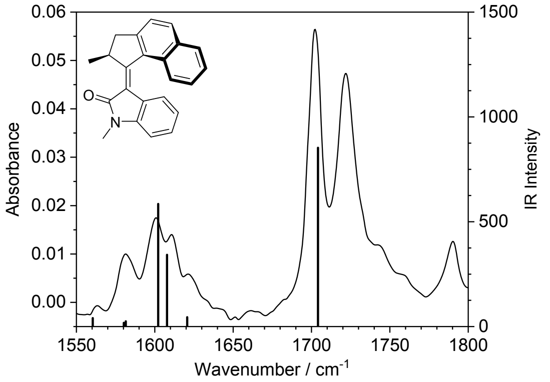

B)

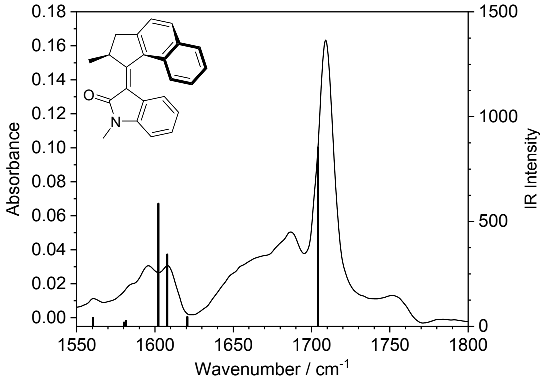

C)

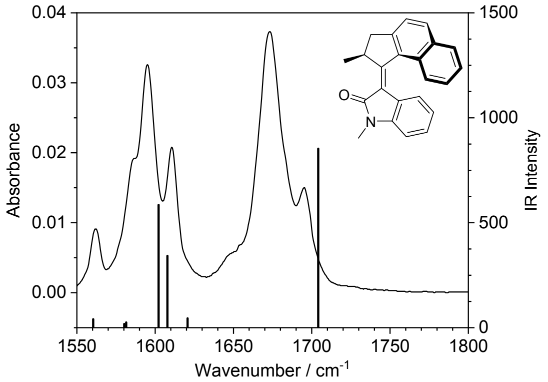

Figure S6: FTIR Spectra for solutions of motor **1** dissolved in **A)** cyclohexane-d_{12} (1.4 mM), **B)** DMSO-d_6 (1.5 mM) and **C)** methanol-d_4 (1.5 mM) all with a path length of 250 μm. The calculated vibrational transitions (shown as black bars) were computed at the ωB97XD/6-31+G(d,p) level of theory for an isolated molecule using a 0.952 scaling factor. The 1704 cm^{-1} transition corresponds to a C=O vibration with a considerable component of C=C vibration of the central alkene bond. The transitions observed from 1550 – 1630 cm^{-1} are attributed to aromatic ring modes, each with a large contribution of vibration of the central C=C alkene bond.

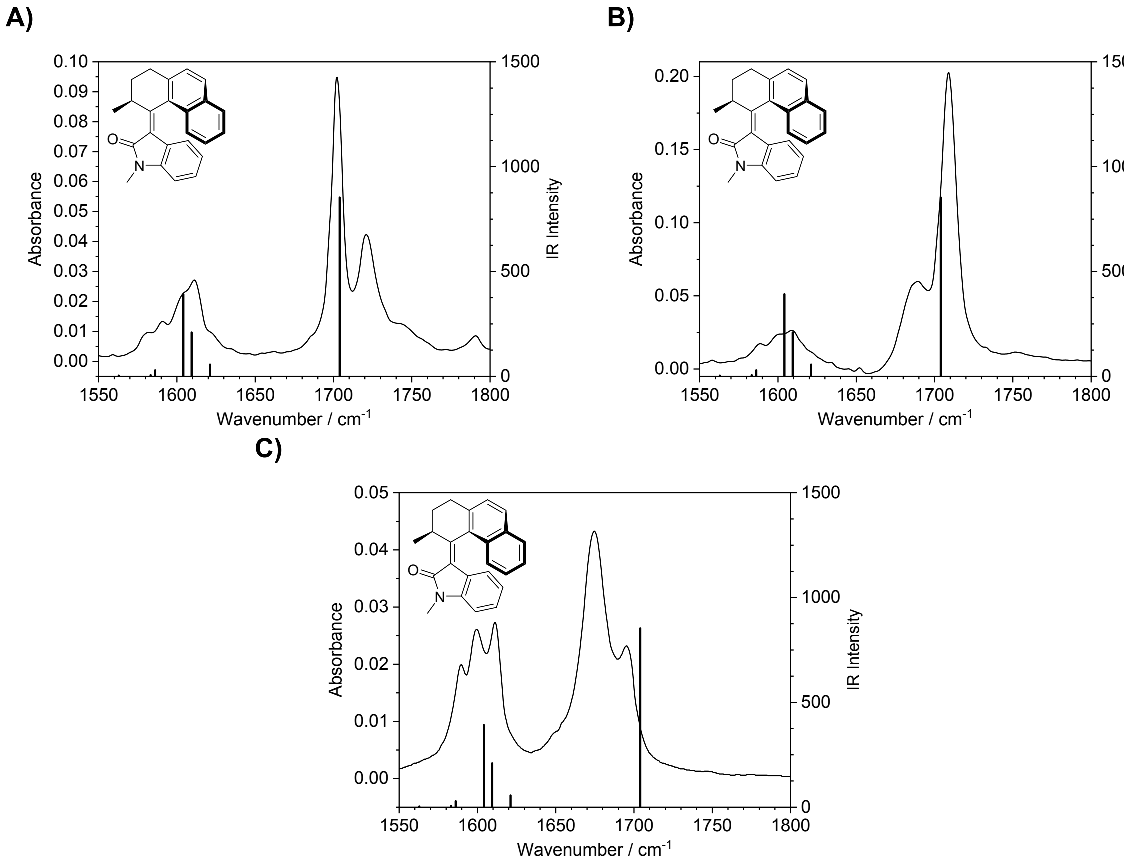

Figure S7: FTIR Spectra for solutions of motor **2** dissolved in **A)** cyclohexane-d_{12} (2.7 mM), **B)** DMSO-d_6 (2.7 mM) and **C)** methanol-d_4 (2.6 mM) all with a path length of 250 µm. The calculated vibrational transitions (shown as black bars) were computed at the ωB97XD/6-31+G(d,p) level of theory for an isolated molecule using a 0.952 scaling factor. The 1704 cm^{-1} transition corresponds to a C=O vibration with a considerable component of C=C vibration of the central alkene bond. The transitions observed from 1580 – 1630 cm^{-1} are attributed to aromatic ring modes, each with a large contribution of vibration of the central C=C alkene bond.

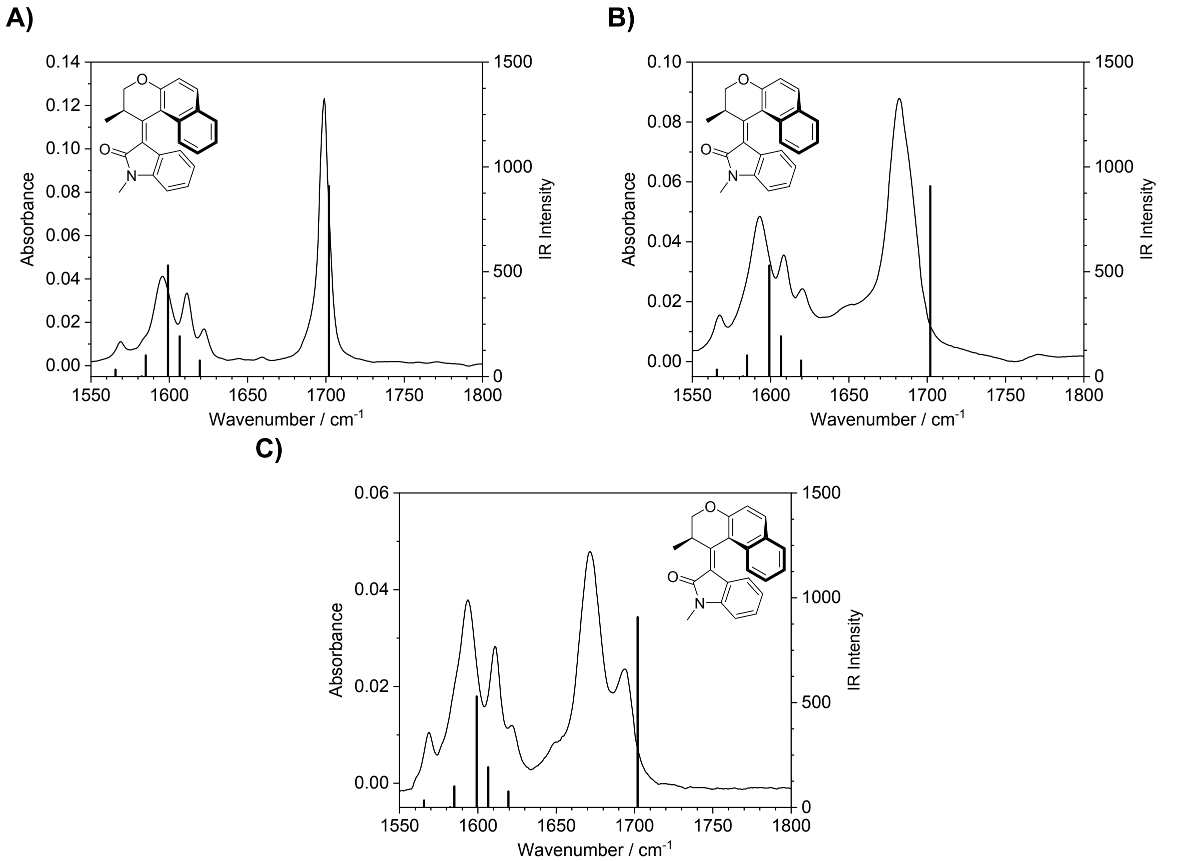

Figure S8: FTIR Spectra for solutions of motor **3** dissolved in **A)** cyclohexane-d_{12} (2.4 mM), **B)** DMSO-d_6 (2.1 mM) and **C)** methanol-d_4 (2.1 mM) all with a path length of 500 μm. The calculated vibrational transitions (shown as black bars) were computed at the ωB97XD/6-31+G(d,p) level of theory for an isolated molecule using a 0.952 scaling factor. The 1702 cm^{-1} transition corresponds to a C=O vibration with a considerable component of C=C vibration of the central alkene bond. The transitions observed from 1560 – 1630 cm^{-1} are attributed to aromatic ring modes, each with a large contribution of vibration of the central C=C alkene bond.

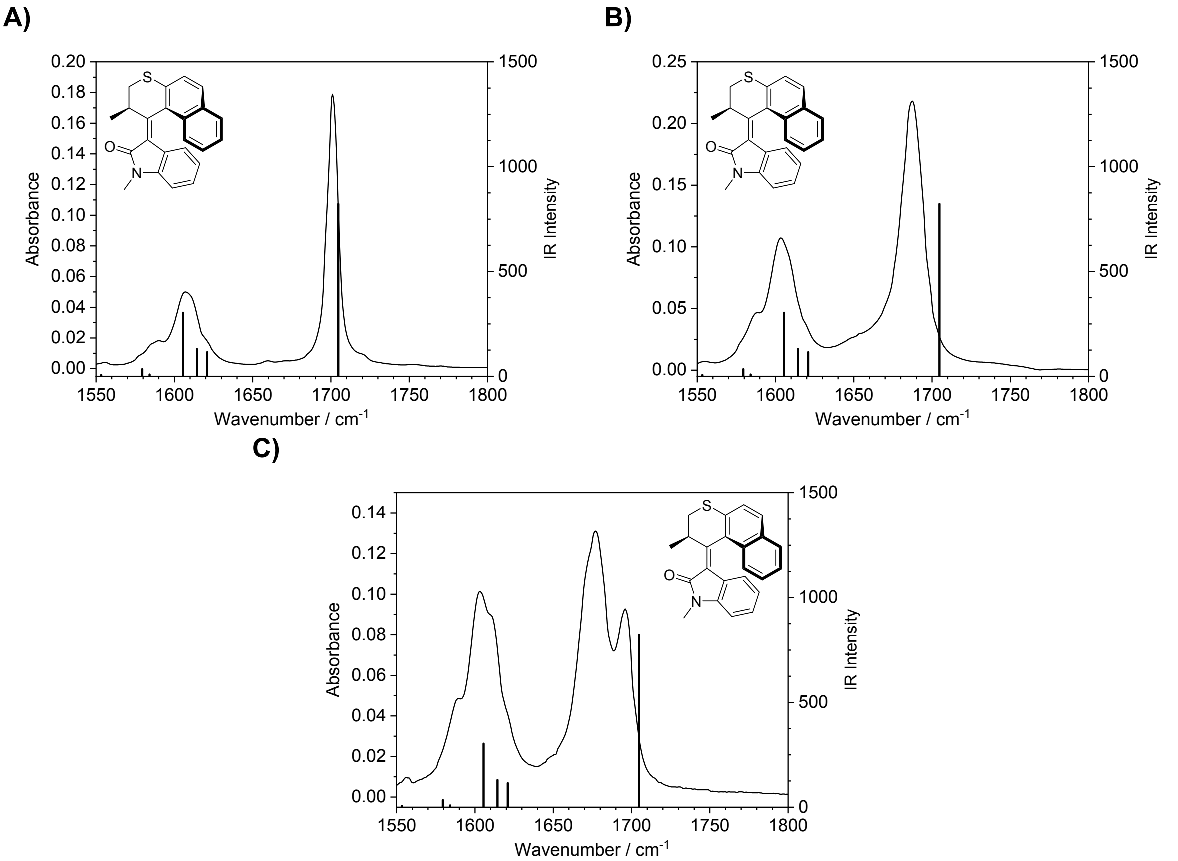

Figure S9: FTIR Spectra for solutions of motor **4** dissolved in **A)** cyclohexane-d_{12} (2.8 mM), **B)** DMSO-d_6 (2.7 mM) and **C)** methanol-d_4 (2.8 mM) all with a path length of 500 µm. The calculated vibrational transitions (shown as black bars) were computed at the ωB97XD/6-31+G(d,p) level of theory for an isolated molecule using a 0.952 scaling factor. The 1705 cm^{-1} transition corresponds to a C=O vibration with a considerable component of C=C vibration of the central alkene bond. The transitions observed from 1570 – 1630 cm^{-1} are attributed to aromatic ring modes, each with a large contribution of vibration of the central C=C alkene bond.

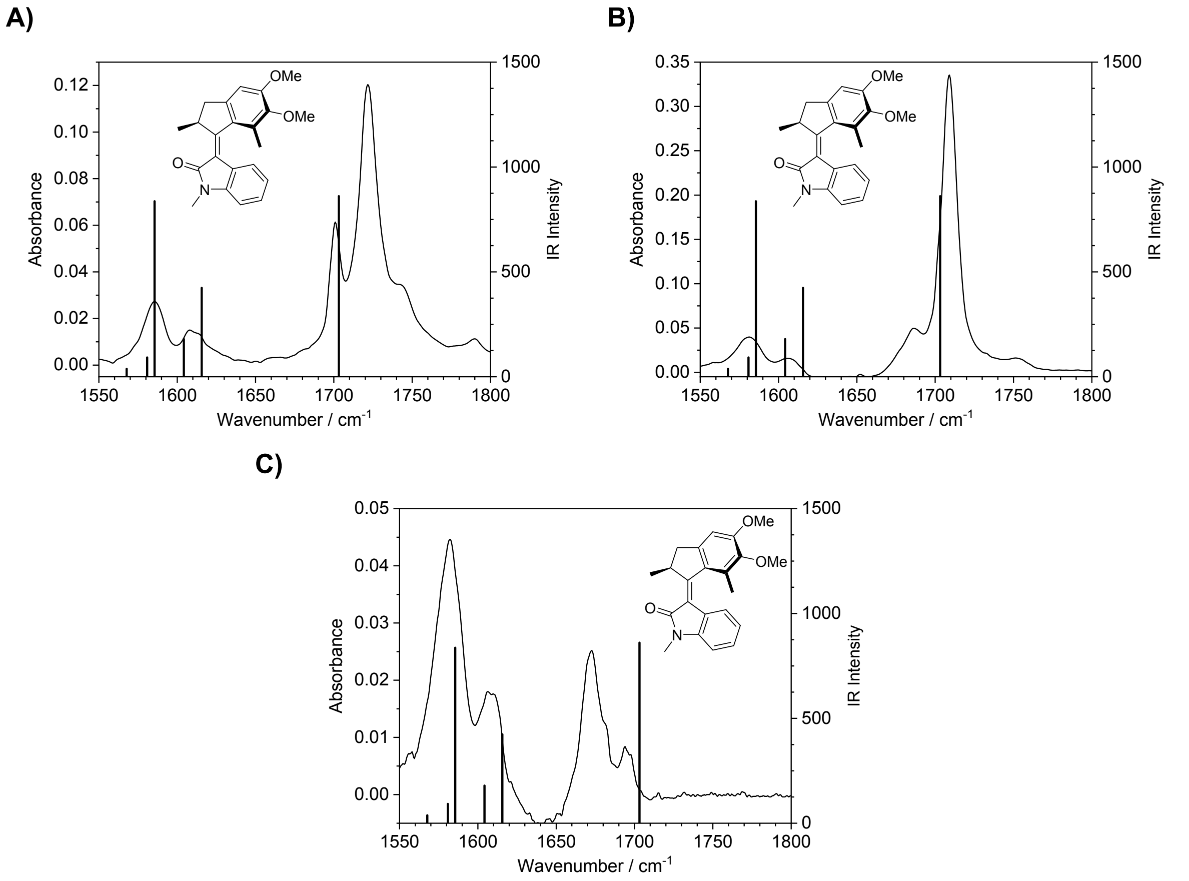

Figure S10: FTIR Spectra for solutions of motor **5** dissolved in **A)** cyclohexane-d_{12} (1.5 mM), **B)** DMSO-d_6 (1.8 mM) and **C)** methanol-d_4 (1.8 mM) all with a path length of 250 μm. The calculated vibrational transitions (shown as black bars) were computed at the ωB97XD/6-31+G(d,p) level of theory for an isolated molecule using a 0.952 scaling factor. The 1703 cm^{-1} transition corresponds to a C=O vibration with a considerable component of C=C vibration of the central alkene bond. The transitions observed from 1510 – 1620 cm^{-1} are attributed to aromatic ring modes, each with a large contribution of vibration of the central C=C alkene bond.

S7 TA and TRIR Spectra and Kinetics of the Oxindole Motors

S7.1 Motor 1 in cyclohexane, DMSO and methanol solutions

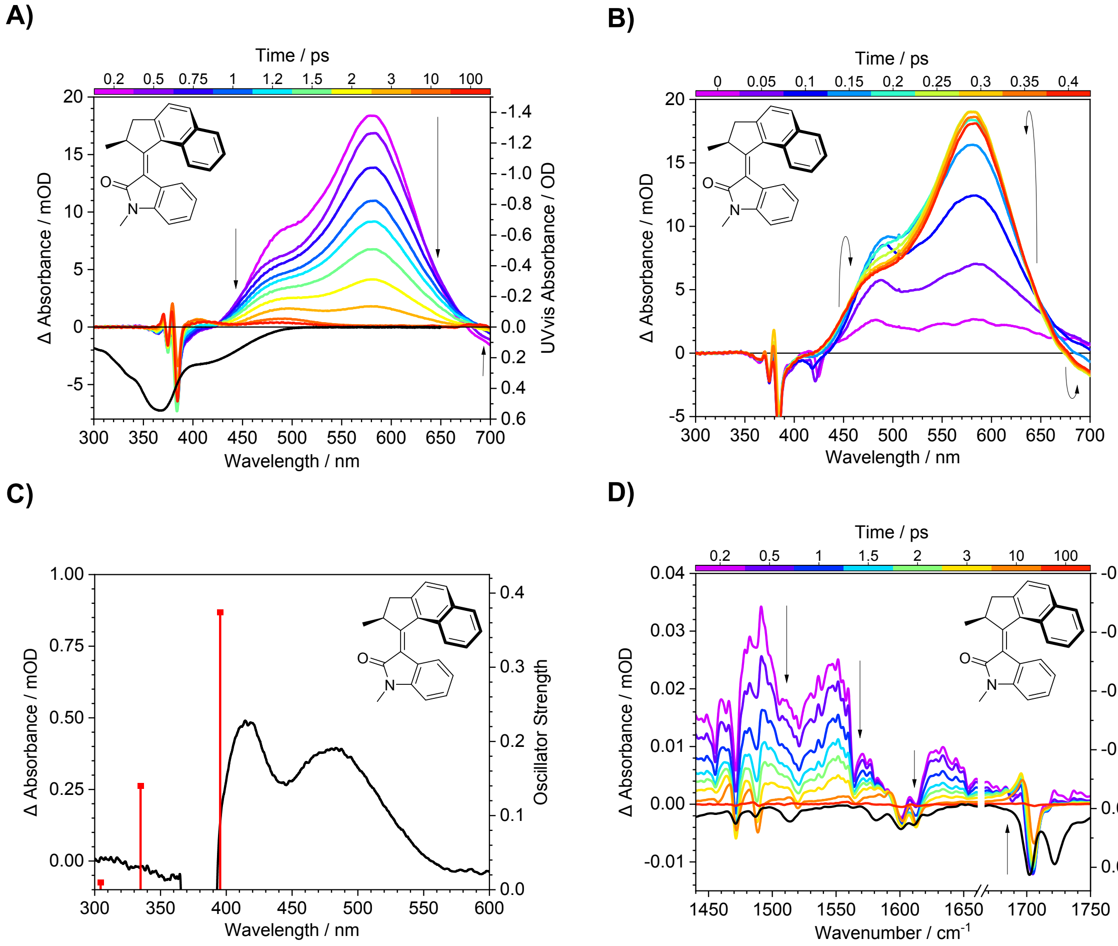

Figure S11: Temporal evolution of the transient absorption spectra of motor **1** in cyclohexane: **A)** for time delays 0.2 – 100 ps, **B)** for time delays 0 – 0.4 ps, **C)** for 900 ps time delay, showing the (*M*,*M*)-*Z*-**1** photoproduct of the isomerization reaction and calculated TD-DFT electronic transitions at the ωB97XD/6-31+G(d,p) level of theory. This spectrum has been smoothed using adjacent averaging in Origin2022b software. **D)** TRIR spectra for motor **1** in cyclohexane-d_{12} for time delays 0.2 – 100 ps. In panel **A)** and **D)**, black curves are inverted steady-state UV-vis and FTIR, respectively, to show regions of parent absorption. In panels **A)**, **B)** and **C)**, strong negative-going signals at 375-nm are scatter of the pump laser radiation. The arrows illustrate how the time-resolved spectral features evolve over time.

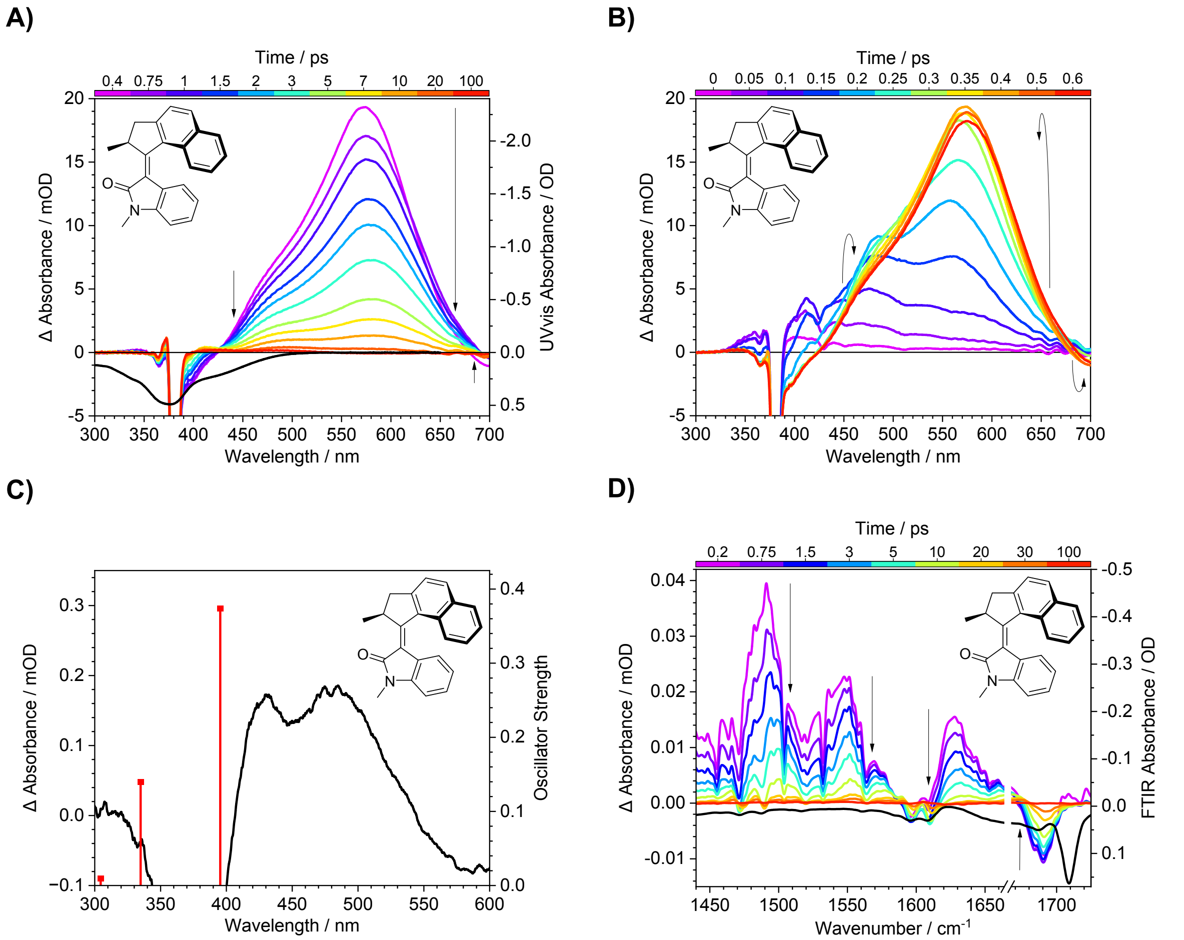

Figure S12: Temporal evolution of the transient absorption spectra of motor **1** in DMSO: **A)** for time delays 0.4 – 100 ps, **B)** for time delays 0 – 0.6 ps, **C)** for 900 ps time delay, showing the (*M*,*M*)-*Z*-**1** photoproduct of the isomerization reaction and calculated TD-DFT electronic transitions at the ωB97XD/6-31+G(d,p) level of theory. This spectrum has been smoothed using adjacent averaging in Origin2022b software. **D)** TRIR spectra for motor **1** in DMSO-d_6 for time delays 0.2 – 100 ps. In panels **A)** and **D)**, black curves are inverted steady-state UV-vis and FTIR, respectively, to show regions of parent absorption. In panels **A)**, **B)** and **C)**, strong negative-going signals at 375-nm are scatter of the pump laser radiation. The arrows illustrate how the time-resolved spectral features evolve over time.

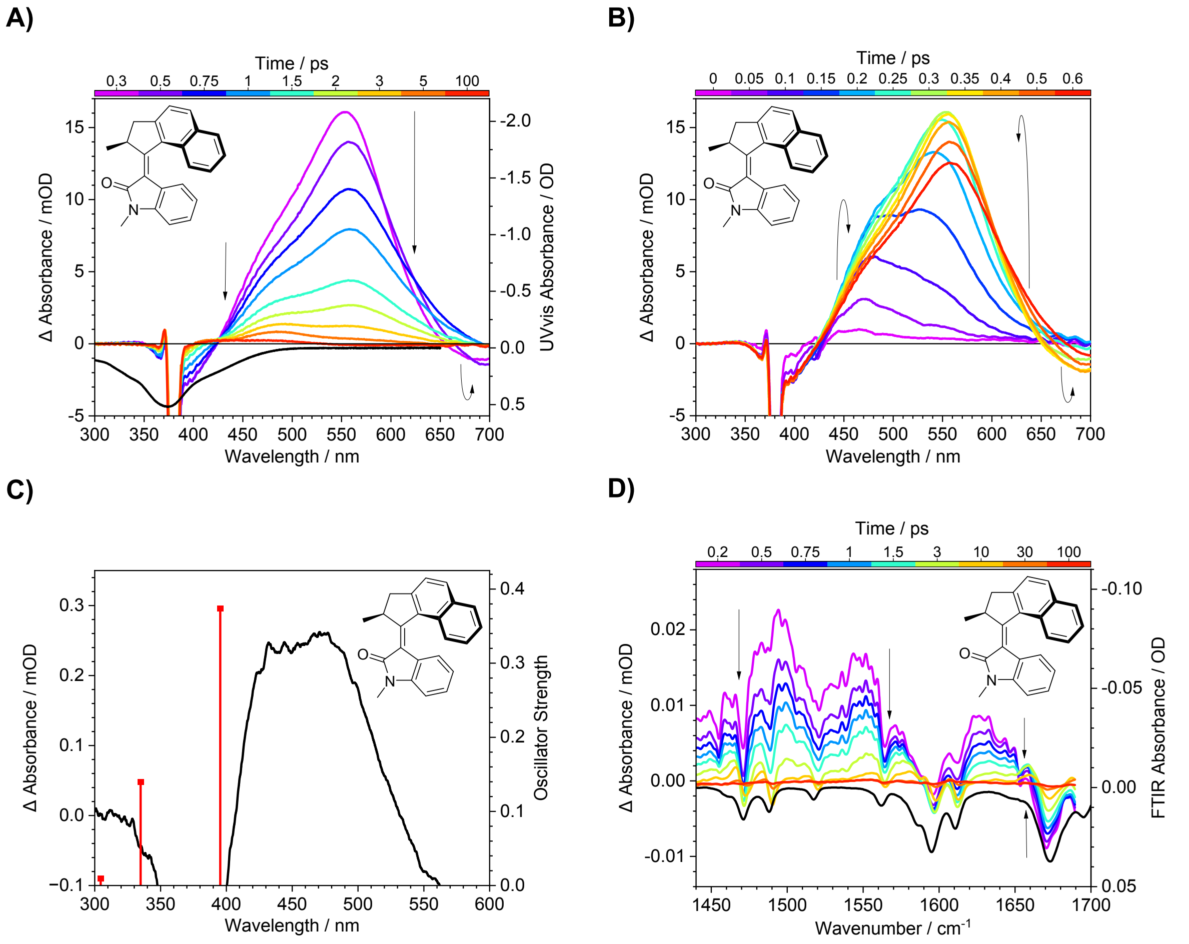

Figure S13: Temporal evolution of the transient absorption spectra of motor **1** in methanol: **A)** for time delays 0.3 – 100 ps, **B)** for time delays 0 – 0.6 ps, **C)** for 900 ps time delay, showing the (*M*,*M*)-*Z*-**1** photoproduct of the isomerization reaction and calculated TD-DFT electronic transitions at the ωB97XD/6-31+G(d,p) level of theory. This spectrum has been smoothed using adjacent averaging in Origin2022b software. **D)** TRIR spectra for motor **1** in methanol-d_4 for time delays 0.2 – 100 ps. In panels **A)** and **D)**, black curves are inverted steady-state UV-vis and FTIR, respectively, to show regions of parent absorption. In panels **A)**, **B)** and **C)**, strong negative-going signals at 375-nm are scatter of the pump laser radiation. The arrows illustrate how the time-resolved spectral features evolve over time.

S7.2 Motor 2 in cyclohexane, DMSO and methanol solutions

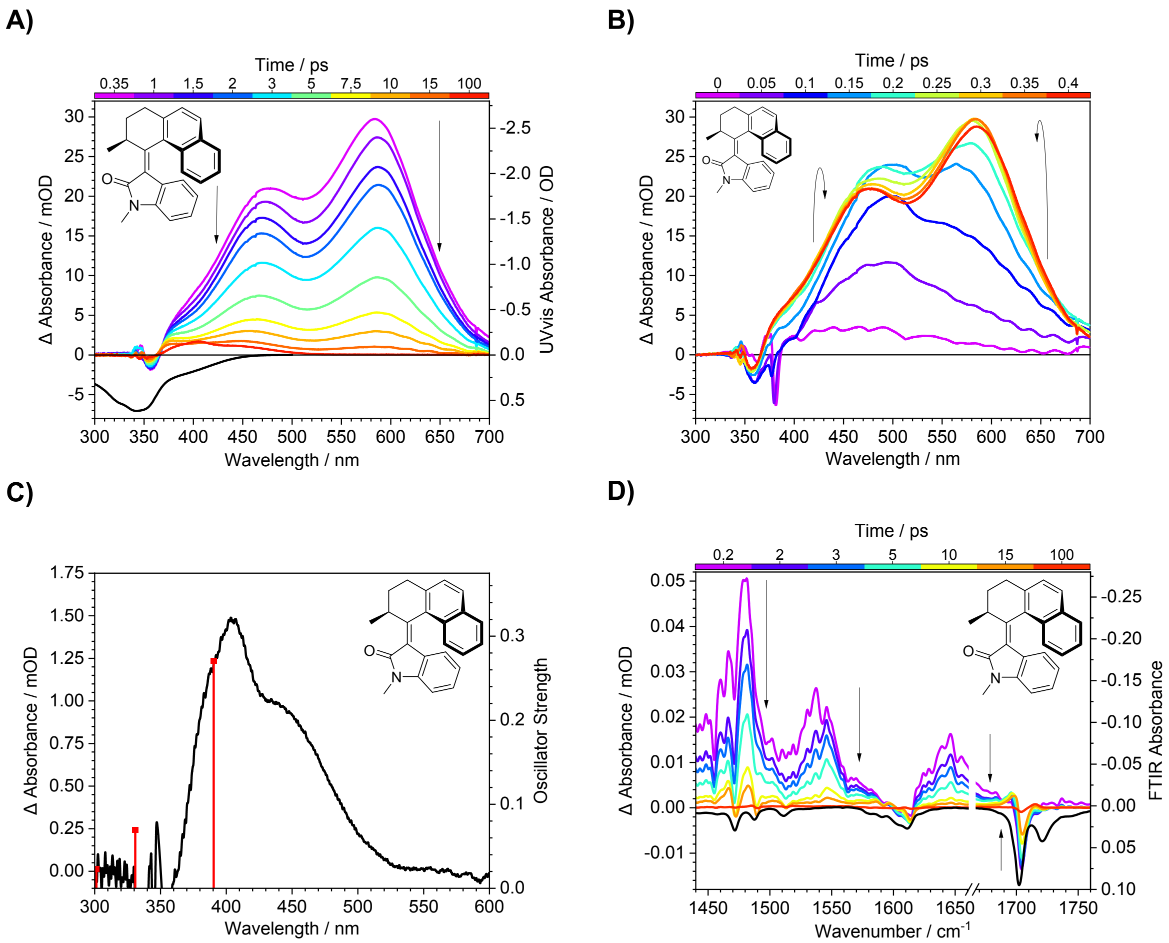

Figure S14: Temporal evolution of the transient absorption spectra of motor **2** in cyclohexane: **A)** for time delays 0.35 – 100 ps, **B)** for time delays 0 – 0.4 ps, **C)** for 900 ps time delay, showing the (*M*,*M*)-*Z*-**2** photoproduct of the isomerization reaction and calculated TD-DFT electronic transitions at the ωB97XD/6-31+G(d,p) level of theory. **D)** TRIR spectra for motor **2** in cyclohexane-d_{12} for time delays 0.2 – 100 ps. In panels **A)** and **D)**, black curves are inverted steady-state UV-vis and FTIR, respectively, to show regions of parent absorption. In panels **A)**, **B)** and **C)**, artifact signals at 345-nm are scatter of the pump laser radiation. The arrows illustrate how the time-resolved spectral features evolve over time.

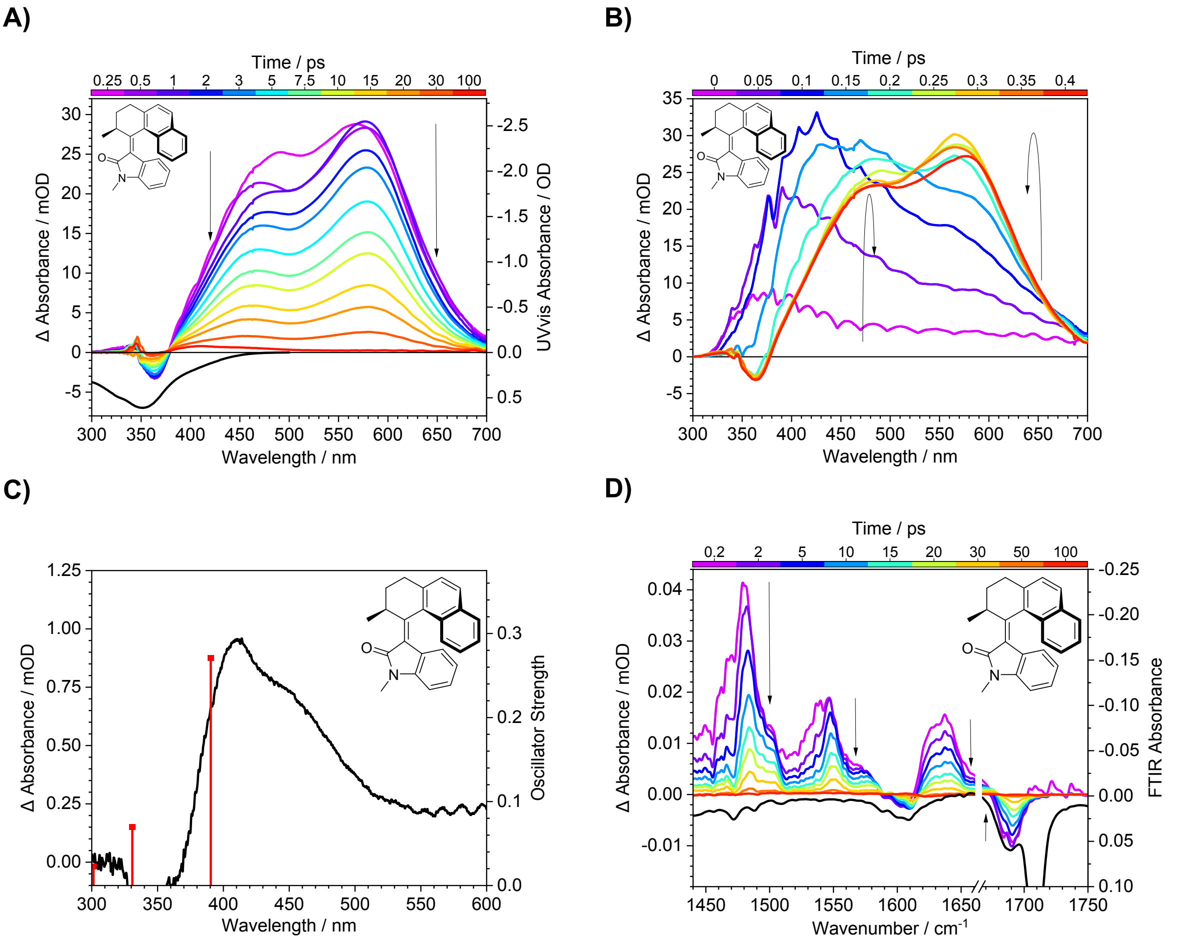

Figure S15: Temporal evolution of the transient absorption spectra of motor **2** in DMSO: **A)** for time delays 0.25 – 100 ps, **B)** for time delays 0 – 0.4 ps, **C)** for 900 ps time delay, showing the (*M*,*M*)-*Z*-**2** photoproduct of the isomerization reaction and calculated TD-DFT electronic transitions at the ωB97XD/6-31+G(d,p) level of theory. **D)** TRIR spectra for motor **2** in DMSO-d_6 for time delays 0.2 – 100 ps. In panels **A)** and **D)**, black curves are inverted steady-state UV-vis and FTIR, respectively, to show regions of parent absorption. In panels **A)**, **B)** and **C)**, artifact signals at 345-nm are scatter of the pump laser radiation. The arrows illustrate how the time-resolved spectral features evolve over time.

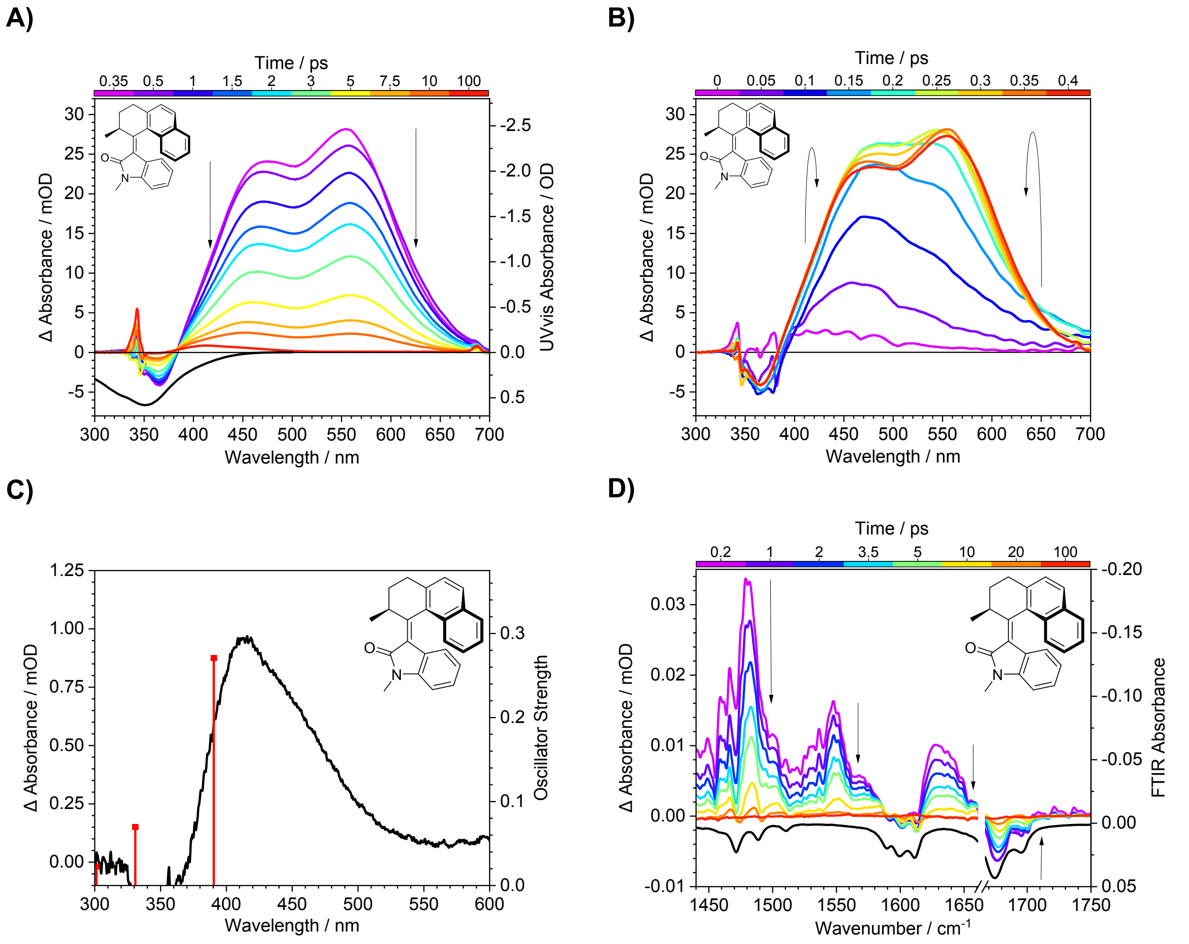

Figure S16: Temporal evolution of the transient absorption spectra of motor **2** in methanol: **A)** for time delays 0.35 – 100 ps, **B)** for time delays 0 – 0.4 ps, **C)** for 900 ps time delay, showing the (*M*,*M*)-*Z*-**2** photoproduct of the isomerization reaction and calculated TD-DFT electronic transitions at the ωB97XD/6-31+G(d,p) level of theory. **D)** TRIR spectra for motor **2** in methanol-d_4 for time delays 0.2 – 100 ps. In panels **A)** and **D)**, black curves are inverted steady-state UV-vis and FTIR, respectively, to show regions of parent absorption. In panels **A)**, **B)** and **C)**, artifact signals at 345-nm are scatter of the pump laser radiation. The arrows illustrate how the time-resolved spectral features evolve over time.

S7.3 Motor 3 in cyclohexane, DMSO and methanol solutions

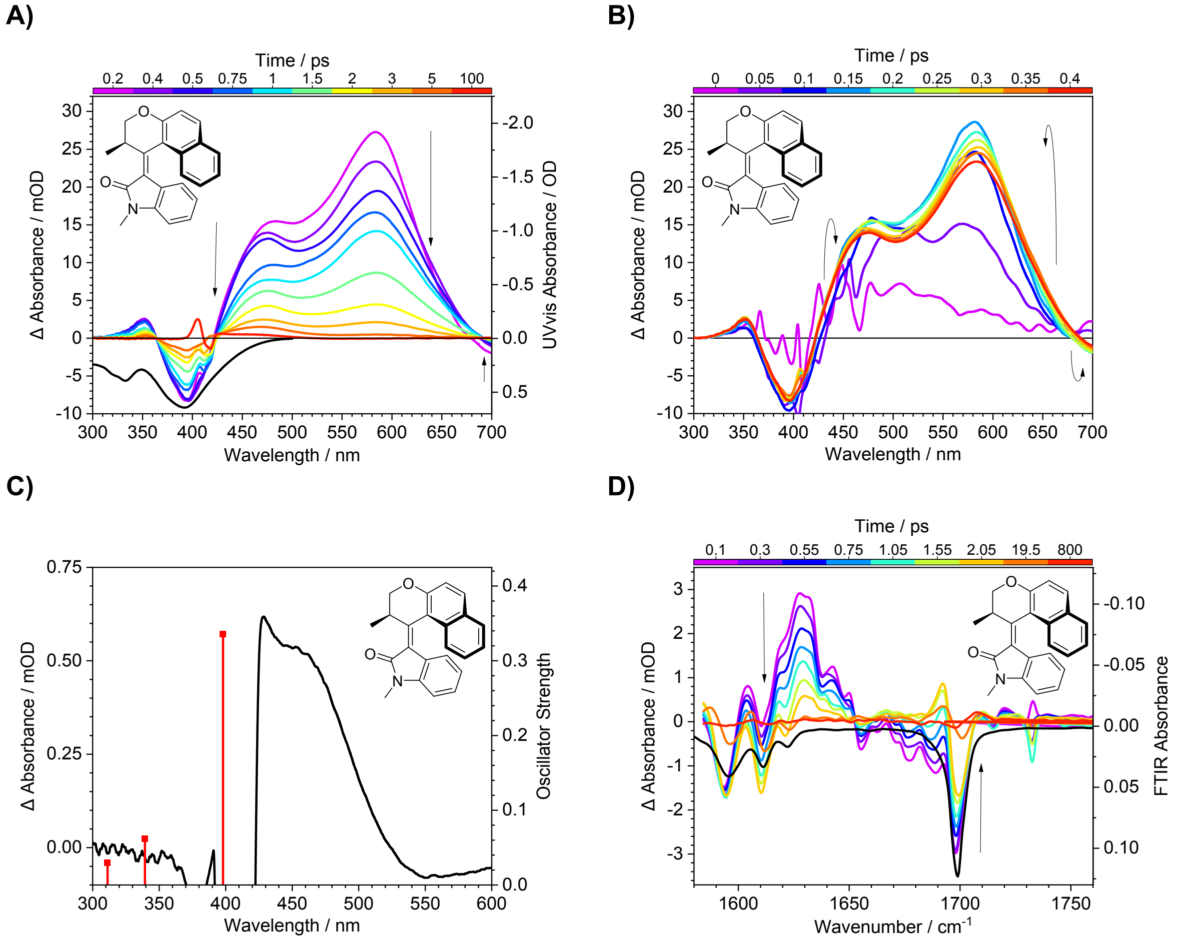

Figure S17: Temporal evolution of the transient absorption spectra of motor **3** in cyclohexane: **A)** for time delays 0.2 – 100 ps, **B)** for time delays 0 – 0.4 ps, **C)** for 900 ps time delay, showing the (*M*,*M*)-*Z*-**3** photoproduct of the isomerization reaction and calculated TD-DFT electronic transitions at the ωB97XD/6-31+G(d,p) level of theory. This spectrum has been smoothed using adjacent averaging in Origin2022b software. **D)** TRIR spectra for motor **3** in cyclohexane-d$_{12}$ for time delays 0.1 – 800 ps. In panels **A)** and **D)**, black curves are inverted steady-state UV-vis and FTIR, respectively, to show regions of parent absorption. In panels **A)**, **B)** and **C)**, artifact signals at 410-nm are scatter of the pump laser radiation. The arrows illustrate how the time-resolved spectral features evolve over time.

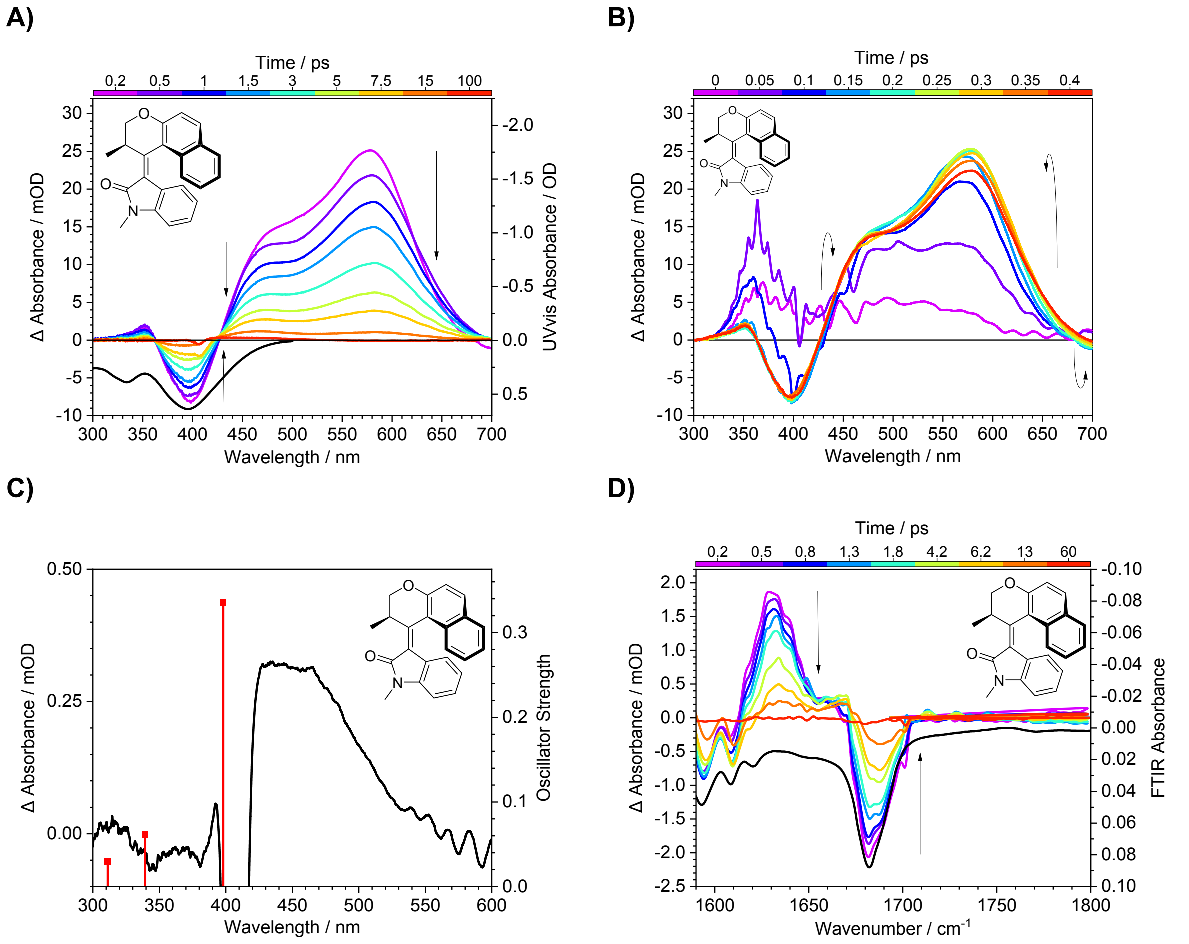

Figure S18: Temporal evolution of the transient absorption spectra of motor **3** in DMSO: **A)** for time delays 0.2 – 100 ps, **B)** for time delays 0 – 0.4 ps, **C)** for 900 ps time delay, showing the (*M*,*M*)-*Z*-**3** photoproduct of the isomerization reaction and calculated TD-DFT electronic transitions at the ωB97XD/6-31+G(d,p) level of theory. This spectrum has been smoothed using adjacent averaging in Origin2022b software. **D)** TRIR spectra for motor 19 in DMSO-d_6 for time delays 0.2 – 60 ps. In panels **A)** and **D)**, black curves are inverted steady-state UV-vis and FTIR, respectively, to show regions of parent absorption. In panels **A)**, **B)** and **C)**, artifact signals at 410-nm are scatter of the pump laser radiation. The arrows illustrate how the time-resolved spectral features evolve over time.

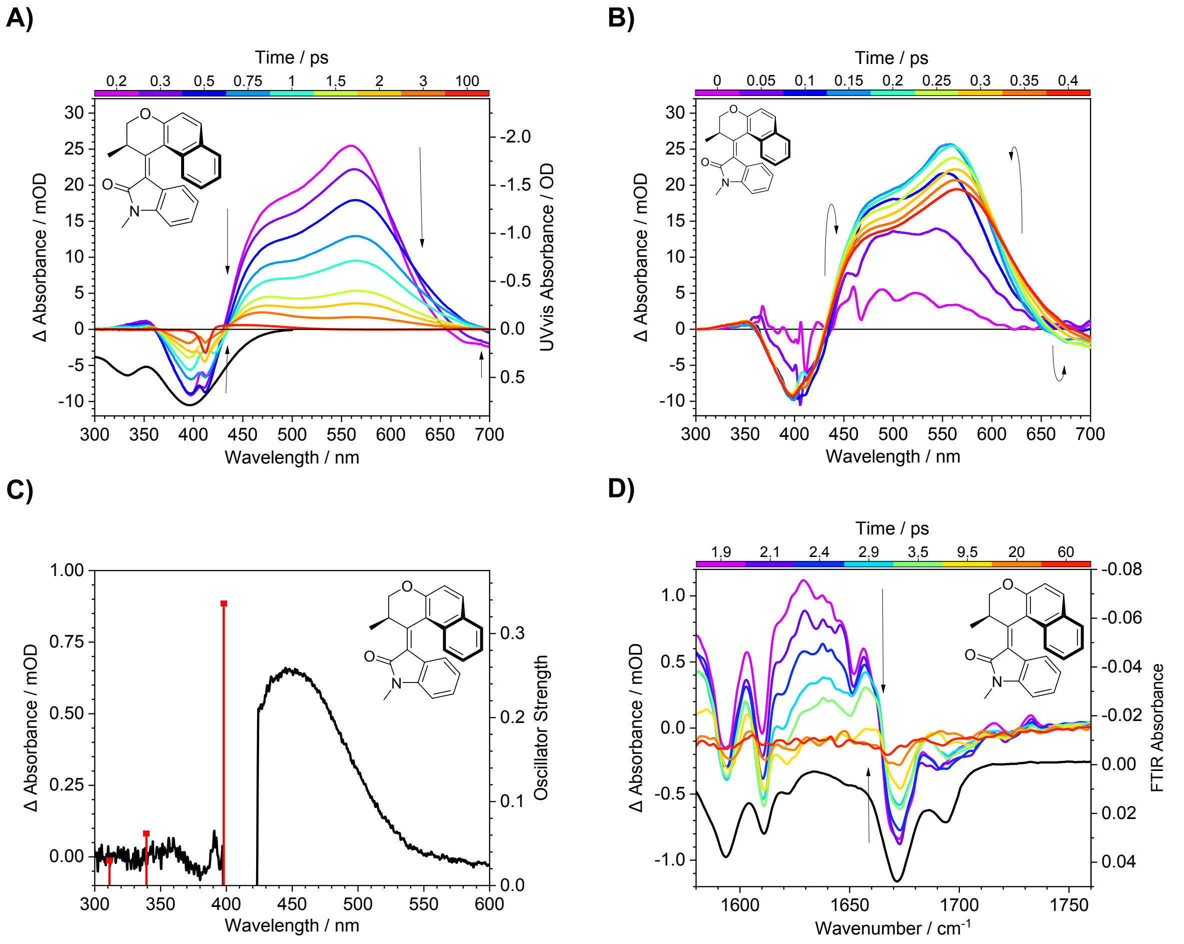

Figure S19: Temporal evolution of the transient absorption spectra of motor **3** in methanol: **A)** for time delays 0.2 – 100 ps, **B)** for time delays 0 – 0.4 ps, **C)** for 900 ps time delay, showing the (*M*,*M*)-*Z*-**3** photoproduct of the isomerization reaction and calculated TD-DFT electronic transitions at the ωB97XD/6-31+G(d,p) level of theory. This spectrum has been smoothed using adjacent averaging in Origin2022b software. **D)** TRIR spectra for motor **3** in methanol-d_4 for time delays 1.9 – 60 ps. In panels **A)** and **D)**, black curves are inverted steady-state UV-vis and FTIR, respectively, to show regions of parent absorption. In panels **A)**, **B)** and **C)**, artifact signals at 410-nm are scatter of the pump laser radiation. The arrows illustrate how the time-resolved spectral features evolve over time.

S7.4 Motor 4 in cyclohexane, DMSO and methanol solutions

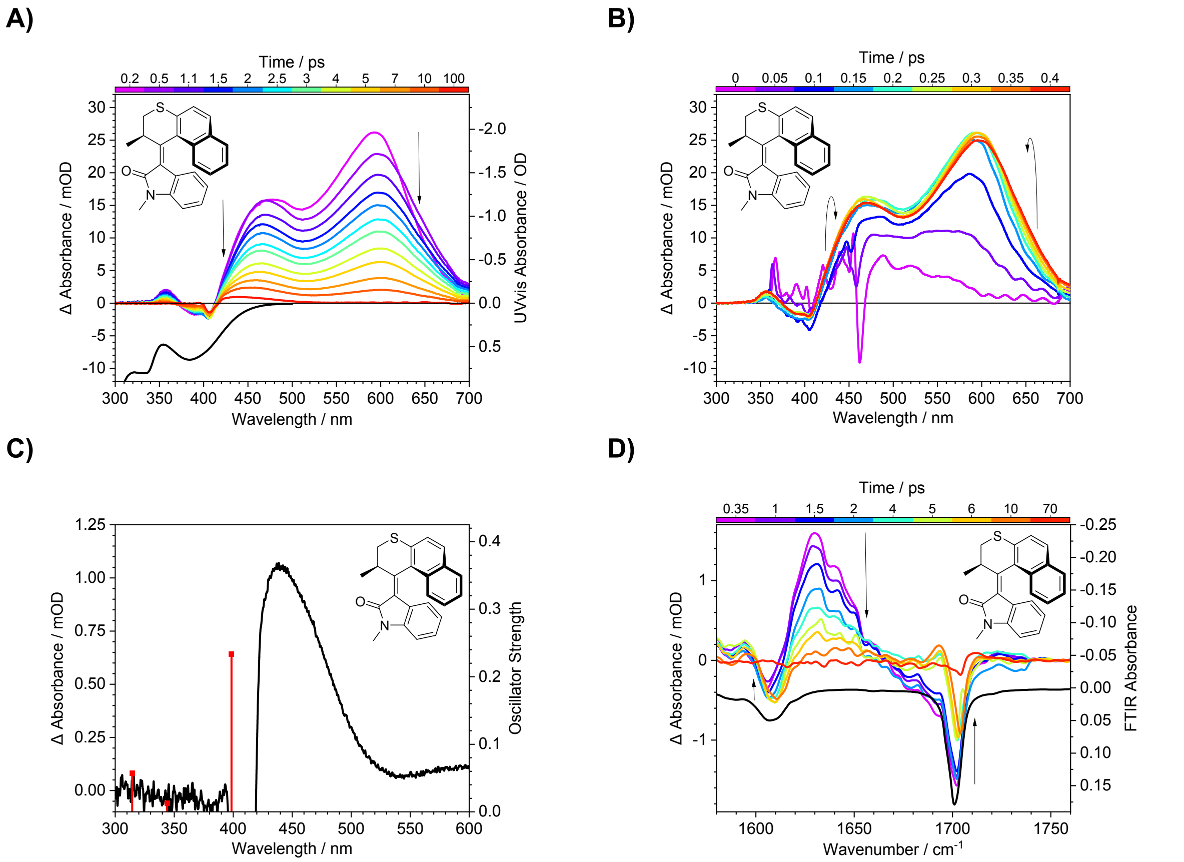

Figure S20: Temporal evolution of the transient absorption spectra of motor **4** in cyclohexane: **A)** for time delays 0.2 – 100 ps, **B)** for time delays 0 – 0.4 ps, **C)** for 900 ps time delay, showing the (*M*,*M*)-*Z*-**4** photoproduct of the isomerization reaction and calculated TD-DFT electronic transitions at the ωB97XD/6-31+G(d,p) level of theory. **D)** TRIR spectra for motor **4** in cyclohexane-d_{12} for time delays 0.35 – 70 ps. In panels **A)** and **D)**, black curves are inverted steady-state UV-vis and FTIR, respectively, to show regions of parent absorption. In panels **A)**, **B)** and **C)**, artifact signals at 410-nm are scatter of the pump laser radiation. The arrows illustrate how the time-resolved spectral features evolve over time.

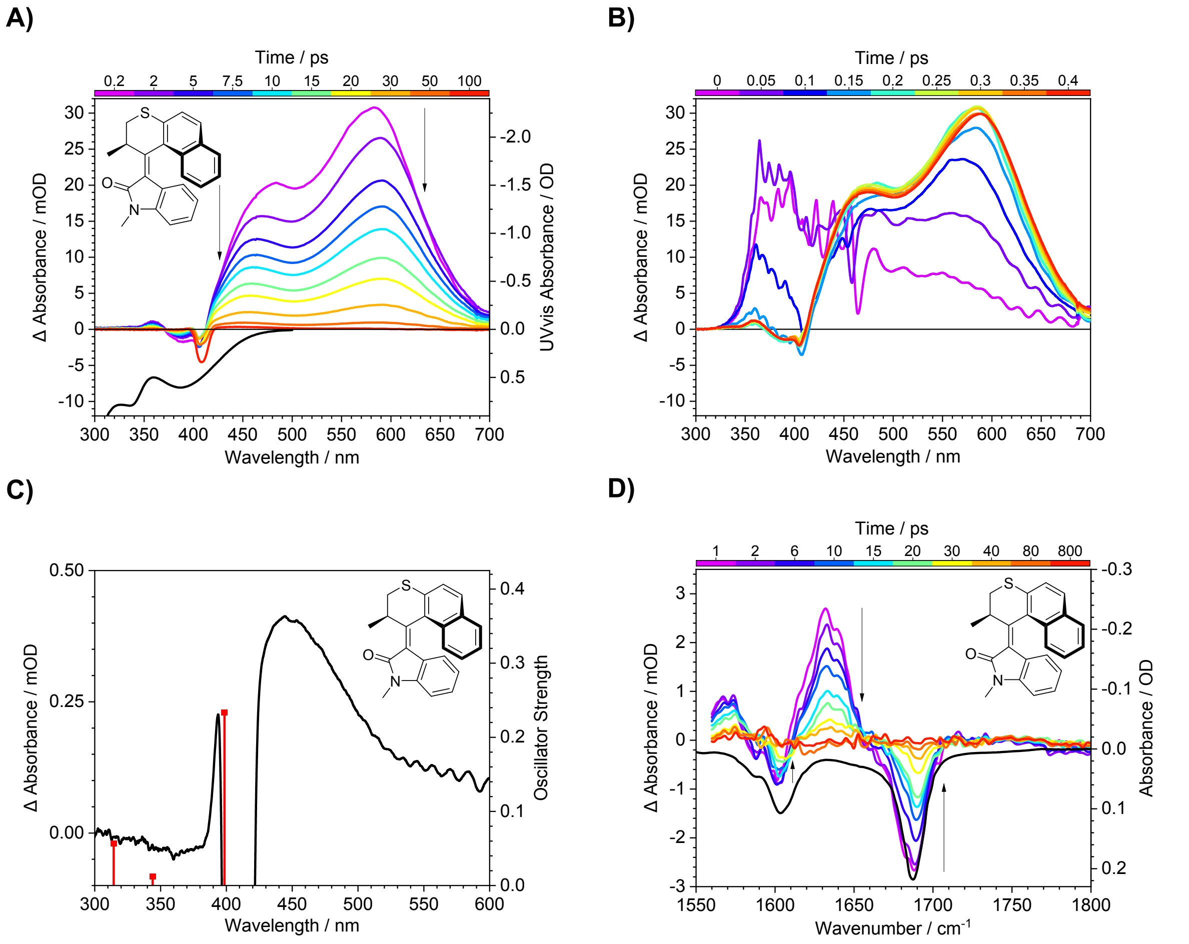

Figure S21: Temporal evolution of the transient absorption spectra of motor **4** in DMSO: **A)** for time delays 0.2 – 100 ps, **B)** for time delays 0 – 0.4 ps, **C)** for 900 ps time delay, showing the (*M*,*M*)-*Z*-**4** photoproduct of the isomerization reaction and calculated TD-DFT electronic transitions at the ωB97XD/6-31+G(d,p) level of theory. This spectrum has been smoothed using adjacent averaging in Origin2022b software. **D)** TRIR spectra for motor 20 in DMSO-d_6 for time delays 1.0 – 800 ps. In panels **A)** and **D)**, black curves are inverted steady-state UV-vis and FTIR, respectively, to show regions of parent absorption. In panels **A)**, **B)** and **C)**, artifact signals at 410-nm are scatter of the pump laser radiation. The arrows illustrate how the time-resolved spectral features evolve over time.

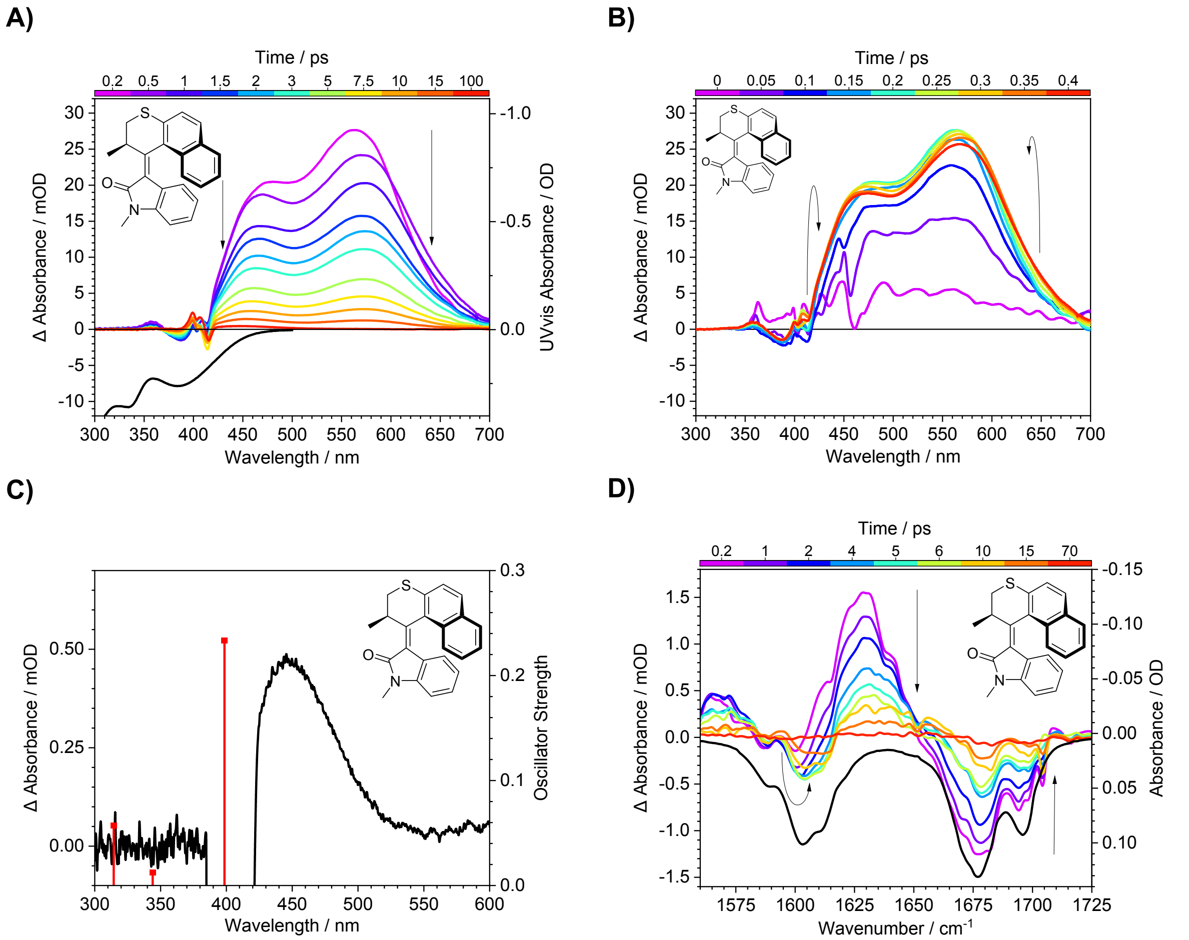

Figure S22: Temporal evolution of the transient absorption spectra of motor **4** in methanol: **A)** for time delays 0.2 – 100 ps, **B)** for time delays 0 – 0.4 ps, **C)** for 900 ps time delay, showing the (*M*,*M*)-*Z*-**4** photoproduct of the isomerization reaction and calculated TD-DFT electronic transitions at the ωB97XD/6-31+G(d,p) level of theory. This spectrum has been smoothed using adjacent averaging in Origin2022b software. **D)** TRIR spectra for motor 20 in methanol-d_4 for time delays 0.2 – 70 ps. In panels **A)** and **D)**, black curves are inverted steady-state UV-vis and FTIR, respectively, to show regions of parent absorption. In panels **A)**, **B)** and **C)**, artifact signals at 410-nm are scatter of the pump laser radiation. The arrows illustrate how the time-resolved spectral features evolve over time.

S7.5 Motor 5 in cyclohexane, DMSO and methanol solutions

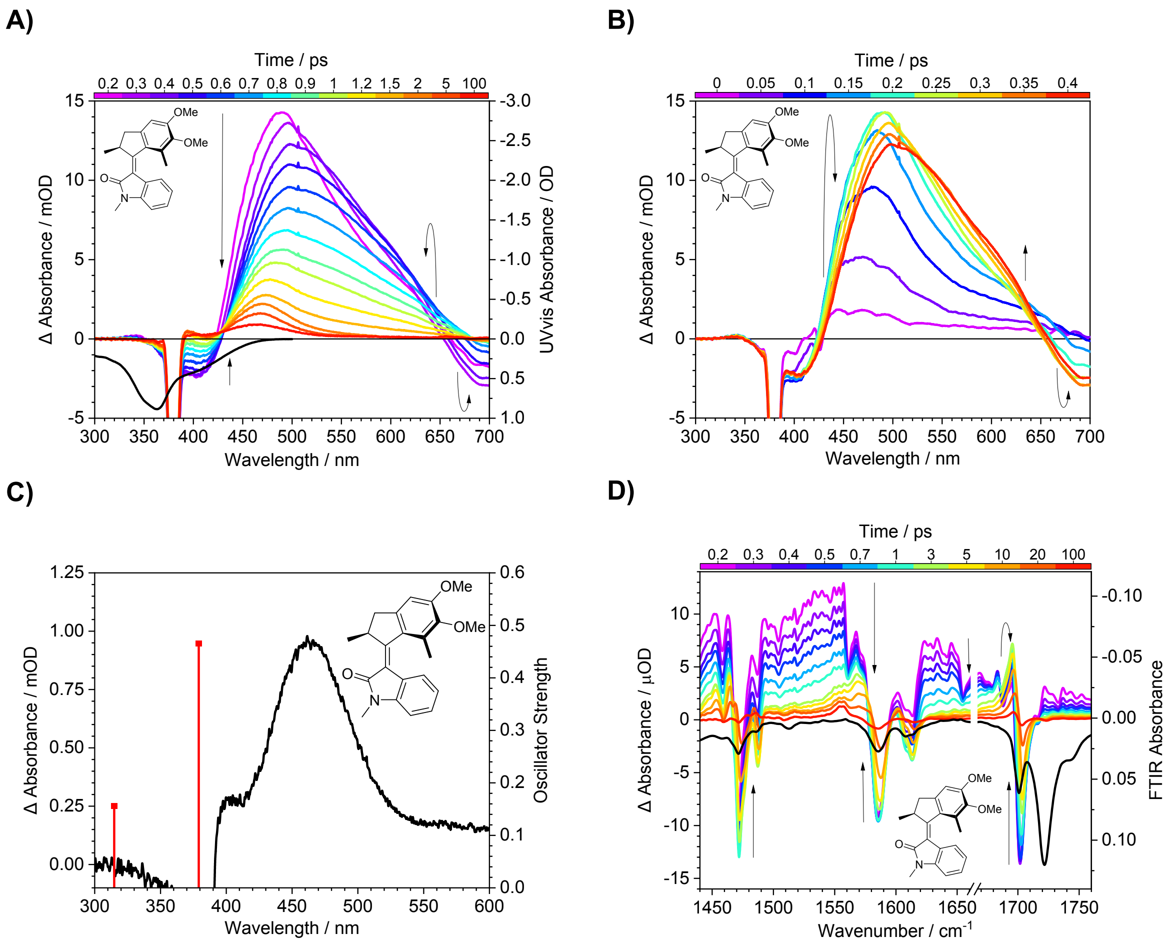

Figure S23: Temporal evolution of the transient absorption spectra of motor **5** in cyclohexane: **A)** for time delays 0.2 – 100 ps, **B)** for time delays 0 – 0.4 ps, **C)** for 900 ps time delay, showing the (*M*,*M*)-*Z*-**5** photoproduct of the isomerization reaction and calculated TD-DFT electronic transitions at the ωB97XD/6-31+G(d,p) level of theory. This spectrum has been smoothed using adjacent averaging in Origin2022b software. **D)** TRIR spectra for motor **5** in cyclohexane-d_{12} for time delays 0.2 – 100 ps. In panels **A)** and **D)**, black curves are inverted steady-state UV-vis and FTIR, respectively, to show regions of parent absorption. In panels **A)**, **B)** and **C)**, strong negative-going signals at 375-nm are scatter of the pump laser radiation. The arrows illustrate how the time-resolved spectral features evolve over time.

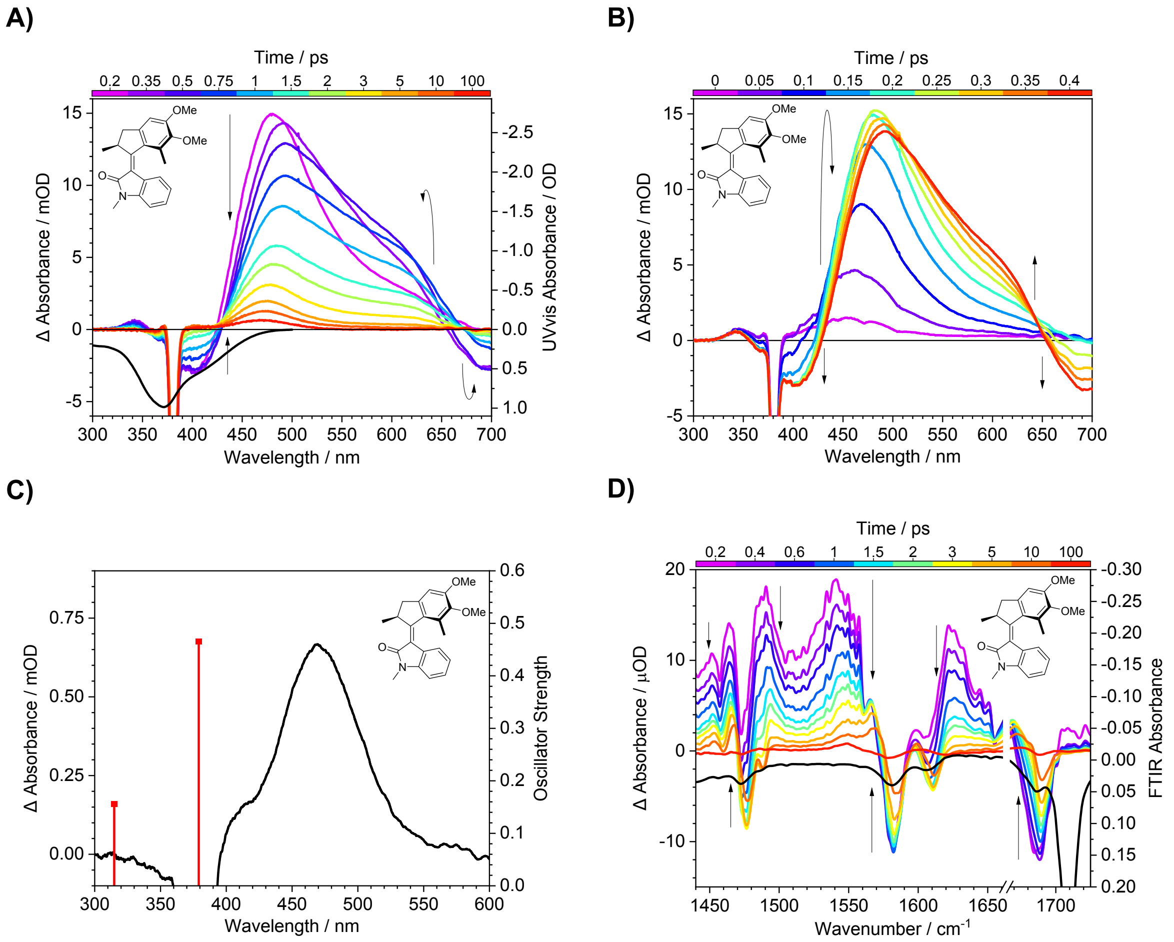

Figure S24: Temporal evolution of the transient absorption spectra of motor **5** in DMSO: **A)** for time delays 0.2 – 100 ps, **B)** for time delays 0 – 0.4 ps, **C)** for 900 ps time delay, showing the (*M*,*M*)-*Z*-**5** photoproduct of the isomerization reaction and calculated TD-DFT electronic transitions at the ωB97XD/6-31+G(d,p) level of theory. This spectrum has been smoothed using adjacent averaging in Origin2022b software. **D)** TRIR spectra for motor **5** in DMSO-d$_6$ for time delays 0.2 – 100 ps. In panels **A)** and **D)**, black curves are inverted steady-state UV-vis and FTIR, respectively, to show regions of parent absorption. In panels **A)**, **B)** and **C)**, strong negative-going signals at 375-nm are scatter of the pump laser radiation. The arrows illustrate how the time-resolved spectral features evolve over time.

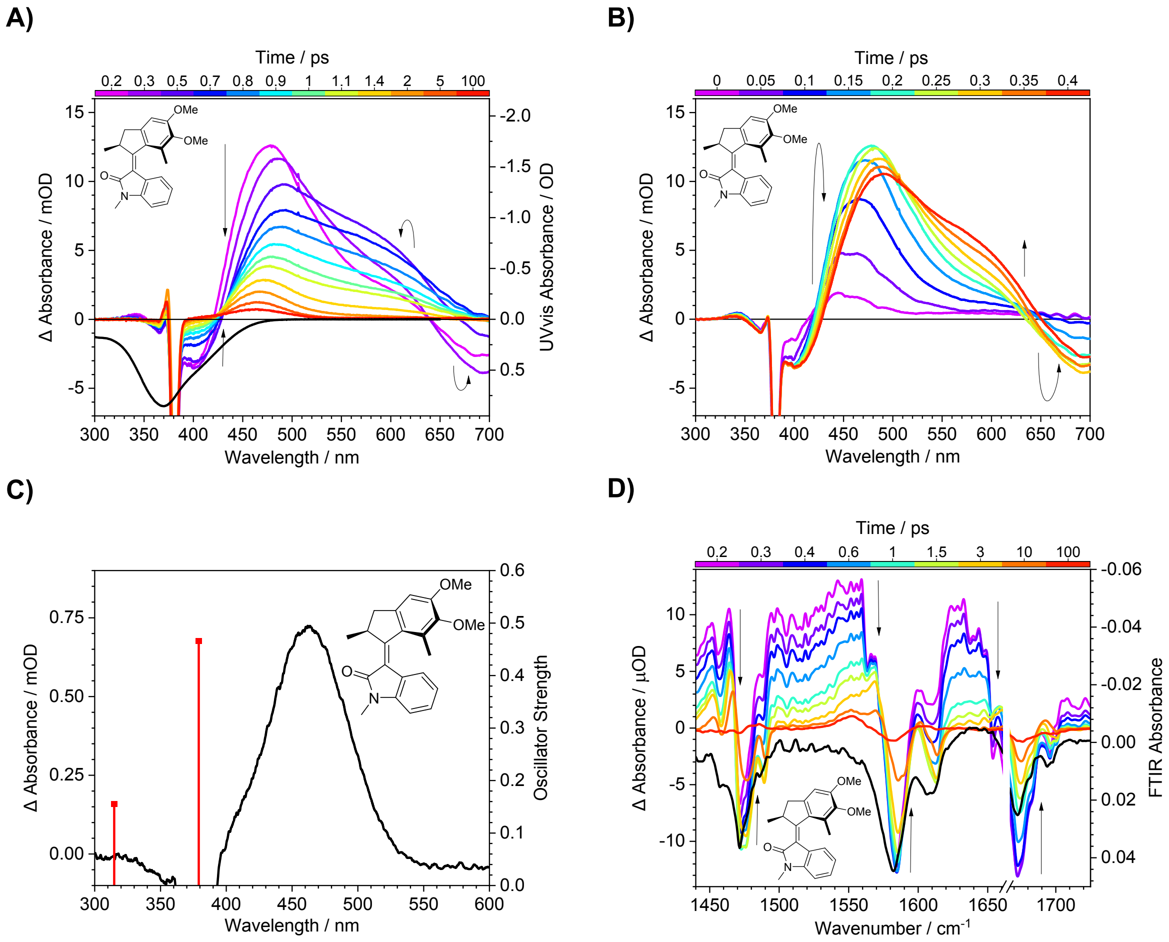

Figure S25: Temporal evolution of the transient absorption spectra of motor **5** in methanol: **A)** for time delays 0.2 – 100 ps, **B)** for time delays 0 – 0.4 ps, **C)** for 900 ps time delay, showing the (*M*,*M*)-*Z*-**5** photoproduct of the isomerization reaction and calculated TD-DFT electronic transitions at the ωB97XD/6-31+G(d,p) level of theory. This spectrum has been smoothed using adjacent averaging in Origin2022b software. **D)** TRIR spectra for motor **5** in methanol-d_4 for time delays 0.2 – 100 ps. In panels **A)** and **D)**, black curves are inverted steady-state UV-vis and FTIR, respectively, to show regions of parent absorption. In panels **A)**, **B)** and **C)**, strong negative-going signals at 375-nm are scatter of the pump laser radiation. The arrows illustrate how the time-resolved spectral features evolve over time.

S8 Examples of TA and TRIR Spectral Decomposition of the Spectra

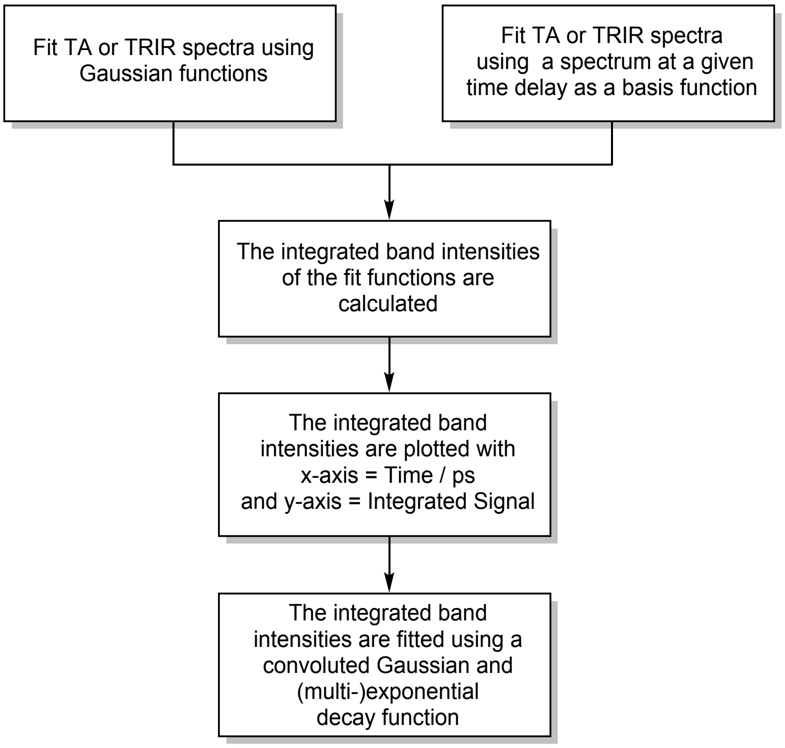

Scheme S2: Flowchart showing the process used to fit the time-resolved spectra to extract time constants using KOALA software.[9]

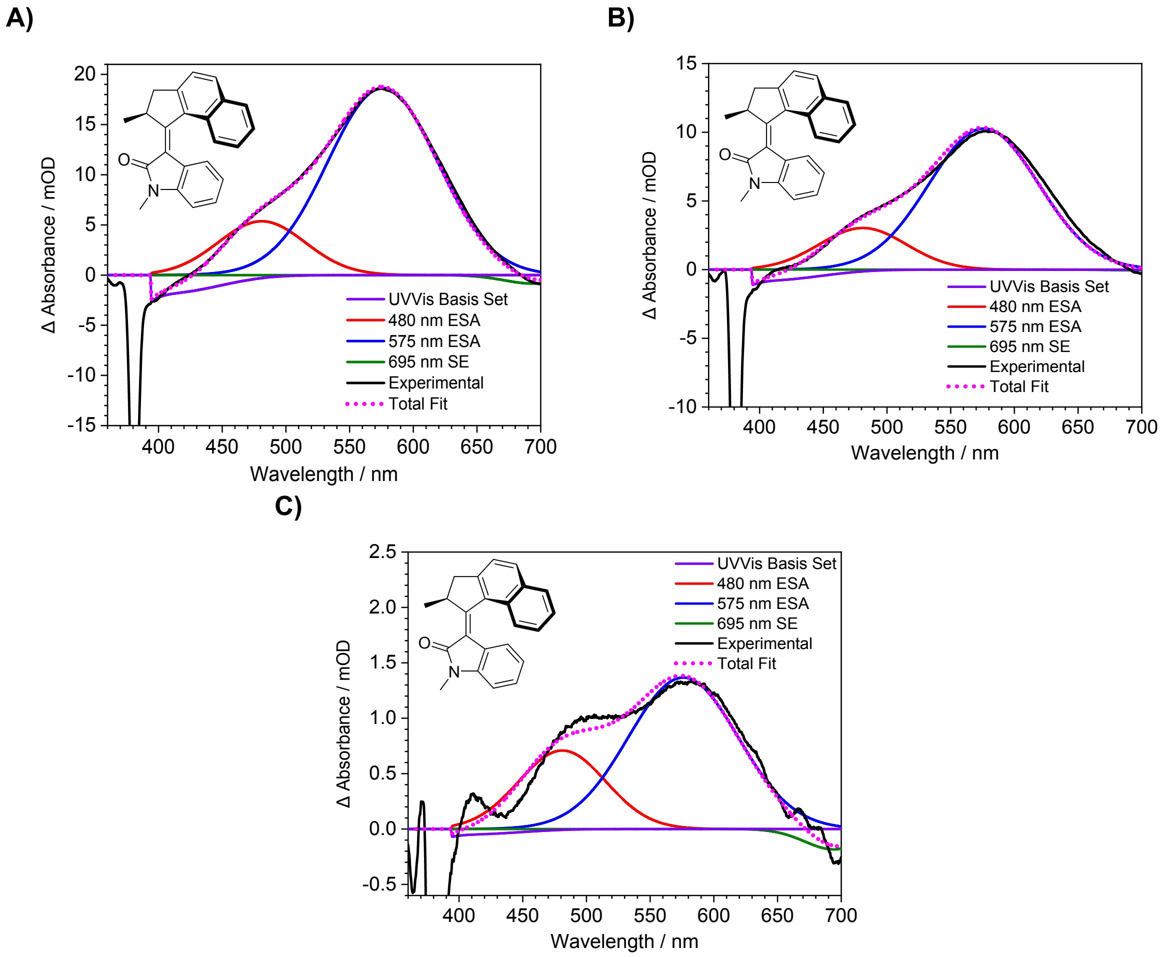

Figure S26: Decomposition of the TA spectra acquired for a 1.4 mM sample of **1** in DMSO following excitation using 375 nm light after **A)** 0.55 ps, **B)** 2 ps and **C)** 10 ps. This is an example of utilizing a combination of the steady-state UV-Vis spectrum and Gaussian functions as basis functions to acquire the integrated band intensities used to obtain the excited-state kinetics for the TA data. Note that the absorbance scale changes in the three panels.

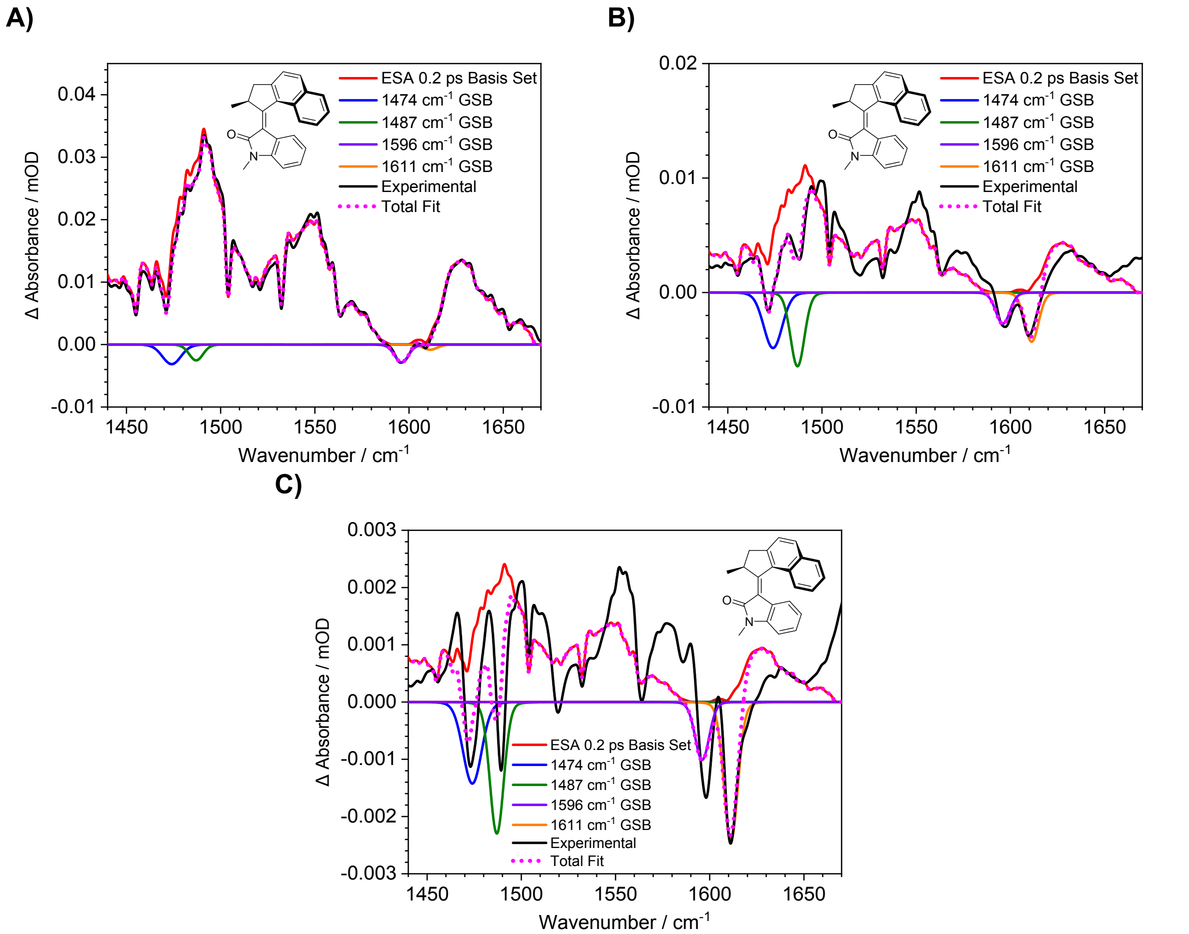

Figure S27: Decomposition of the TRIR spectra acquired for a 1.4 mM sample of **1** in DMSO-d_6 following excitation using 375 nm light after **A)** 0.5 ps, **B)** 5 ps and **C)** 15 ps. This is an example of utilizing a combination of the 0.2 ps spectrum as a basis function and Gaussian basis functions for GSB features to acquire the integrated band intensities used to obtain the excited-state kinetics for the TRIR data. Note that the absorbance scale changes in the three panels.

S9 Kinetics of Excited-State Relaxation for the Oxindole Motors

S9.1 Motor 1 in cyclohexane, DMSO and methanol solutions

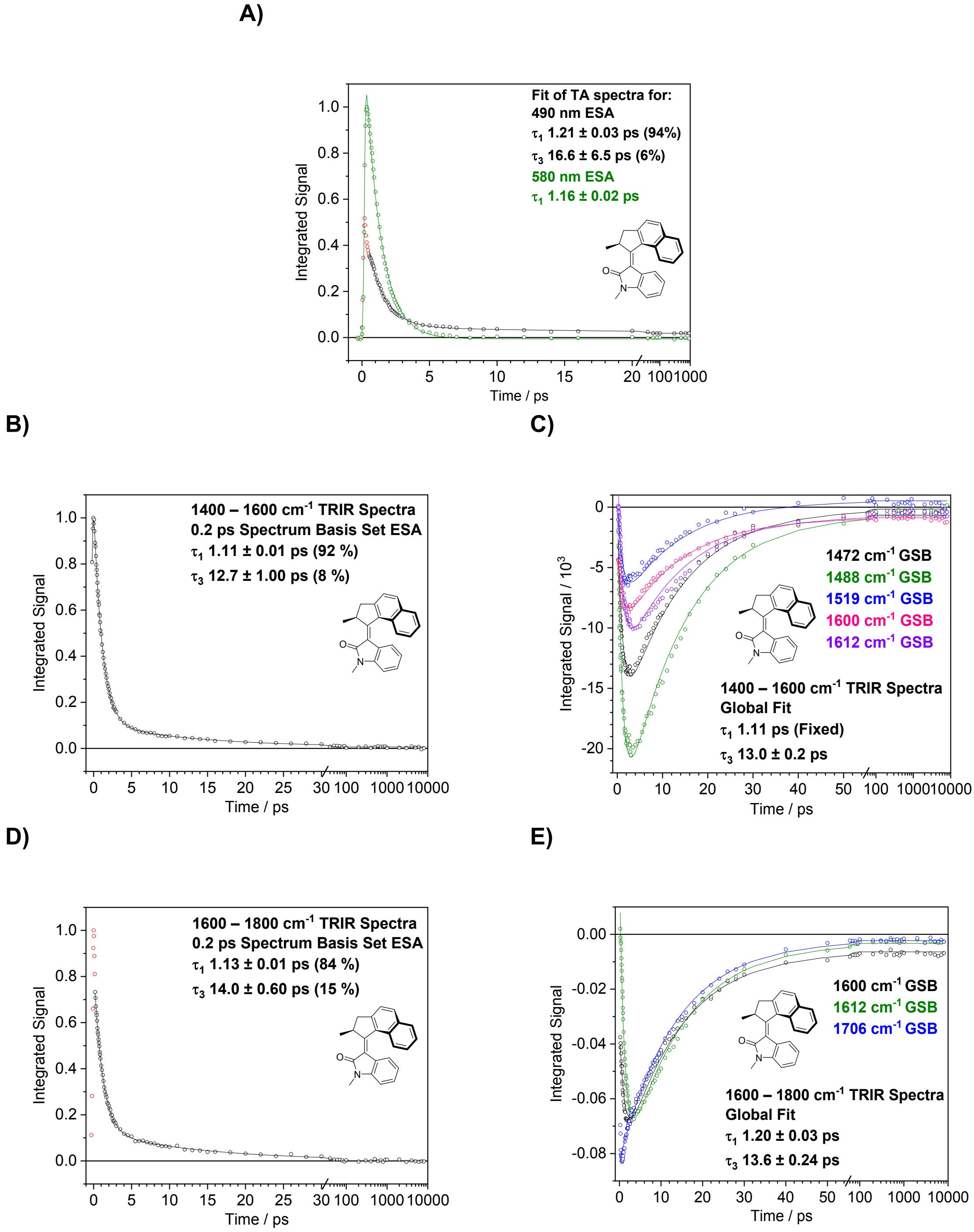

Figure S28: **A)** Excited-state kinetics deduced from the TA spectra of **1** acquired in cyclohexane, highlighting mono- and bi-exponential decays fitted to integrated band intensities obtained by spectral decomposition using the two Gaussian functions centred at the wavelengths stated in the inset. The < 0.55 ps 490 nm data (red circles) are omitted from the fitting procedure due to non-exponential decay kinetics caused by ultrafast kinetics in the S_1 adiabatic PES. **B)** and **D)** Bi-exponential excited-state kinetics of **1** from TRIR spectra in cyclohexane-d_{12} collected in the 1400 – 1600 cm^{-1} and 1600 – 1800 cm^{-1} regions, respectively. These kinetics were obtained using the integrated band intensities acquired by fitting of each spectral region using their respective 0.2 ps spectra as a basis function. **C)** and **E)** Kinetics of the GSB bands obtained from 1400 – 1600 cm^{-1} and 1600 – 1800 cm^{-1} regions, respectively, fitted to integrated band intensities derived by spectral fitting using Gaussian functions centred at the wavenumbers stated in the inset.

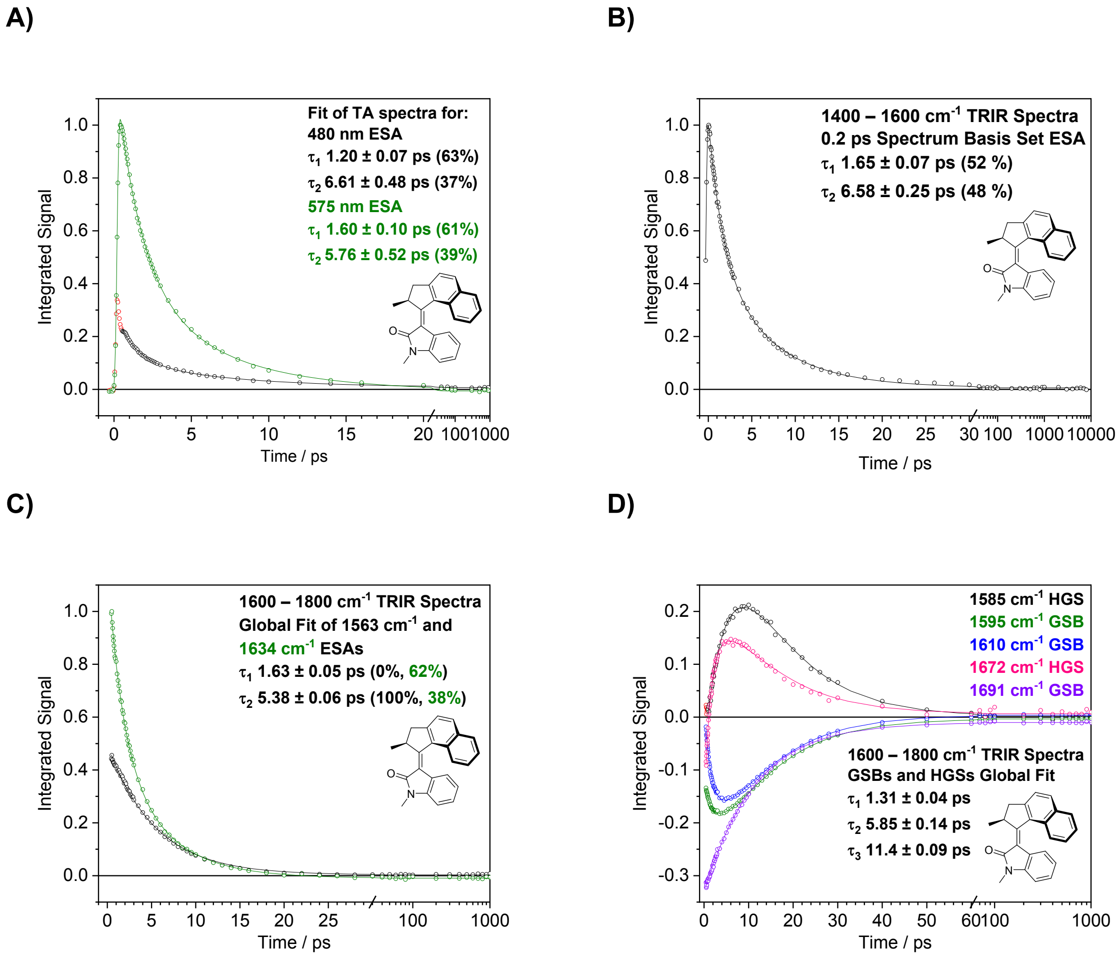

Figure S29: **A)** Excited-state kinetics deduced from the TA spectra of **1** acquired in DMSO, highlighting bi-exponential decay kinetics fitted to integrated band intensities obtained by spectral decomposition using the two Gaussian functions centred at the wavelengths stated in the inset. The < 0.55 ps 480 nm data (red circles) are omitted from the fitting procedure due to non-exponential decay kinetics caused by ultrafast kinetics in the S_1 adiabatic potential. **B)** and **C)** Bi-exponential excited-state kinetics of **1** from TRIR spectra in DMSO-d_6 collected in the 1400 – 1600 cm^{-1} and 1600 – 1800 cm^{-1} regions, respectively. These kinetics were obtained using **B)** the integrated band intensities acquired by spectral fitting using the 0.2 ps spectra as a basis function and **C)** Gaussian functions centred at the wavenumbers stated in the inset. **D)** Kinetics of the GSB and HGS bands obtained from 1600 – 1800 cm^{-1} region, fitted to integrated band intensities derived by spectral fitting using Gaussian functions centred at the wavenumbers stated in the inset.

A)

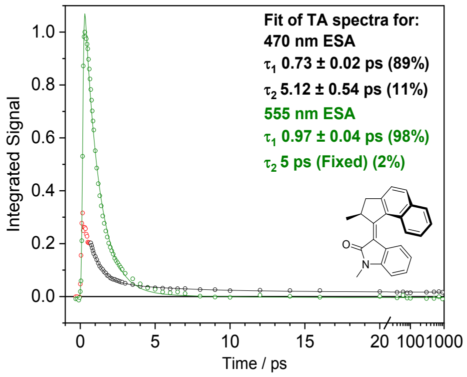

B)

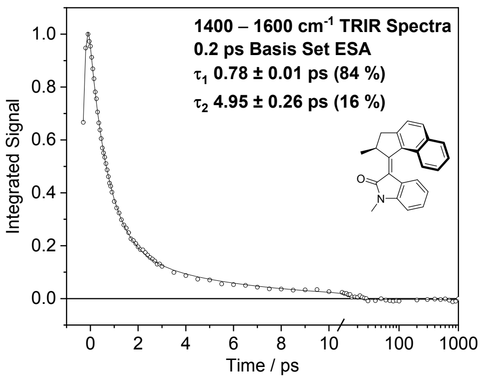

C)

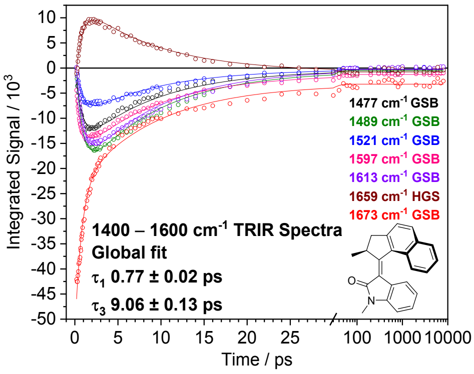

Figure S30: **A)** Excited-state kinetics deduced from the TA spectra of **1** acquired in methanol, highlighting bi-exponential decay kinetics fitted to integrated band intensities obtained by spectral decomposition using the two Gaussian functions centred at the wavelengths stated in the inset. The < 0.7 ps 470 nm data (red circles) are omitted from the fitting procedure due to non-exponential decay kinetics caused by ultrafast kinetics in the S_1 adiabatic potential. **B)** Bi-exponential excited-state kinetics of **1** from TRIR spectra in methanol-d_4 collected in the 1400 – 1600 cm^{-1} region. These kinetics were obtained using the integrated band intensities acquired by spectral fitting using the 0.2 ps spectra as a basis function. **C)** Kinetics of the GSB and HGS bands obtained from 1400 – 1600 cm^{-1} region, fitted to integrated band intensities derived by spectral fitting using Gaussian functions centred at the wavenumbers stated in the inset.

S9.2 Motor 2 in cyclohexane, DMSO and methanol solutions

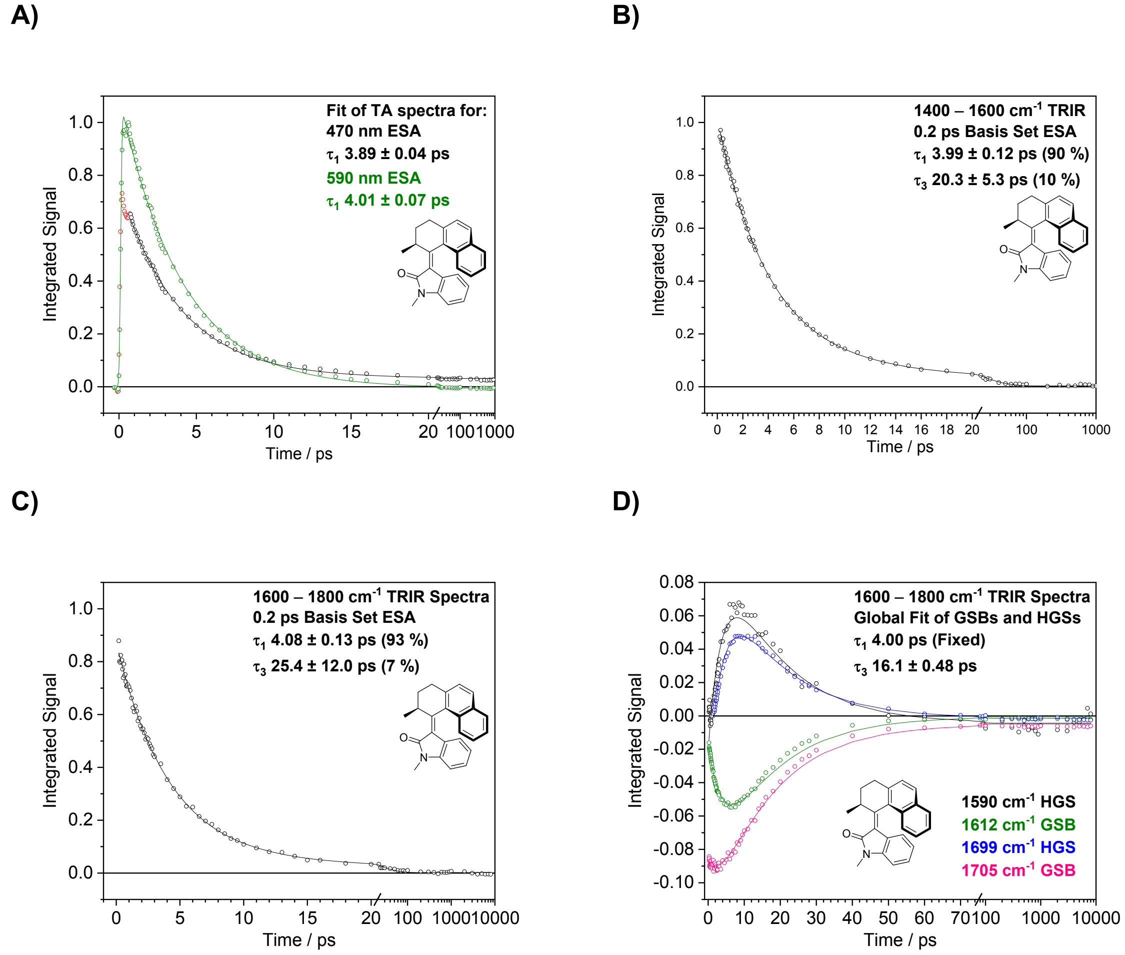

Figure S31: **A)** Excited-state kinetics deduced from the TA spectra of **2** acquired in cyclohexane, highlighting mono-exponential decay kinetics fitted to integrated band intensities obtained by spectral decomposition using the two Gaussian functions centred at the wavelengths stated in the inset. The < 0.75 ps 470 nm data (red circles) are omitted from the fitting procedure due to non-exponential decay kinetics caused by ultrafast kinetics in the S_1 adiabatic potential. **B)** and **C)** Bi-exponential excited-state kinetics of **2** from TRIR spectra in cyclohexane-d_{12} collected in the 1400 – 1600 cm^{-1} and 1600 – 1800 cm^{-1} regions, respectively. These kinetics were obtained using the integrated band intensities acquired by spectral fitting of each spectral region using their respective 0.2 ps spectra as a basis function. **D)** Kinetics of the GSB and HGS bands obtained from 1600 – 1800 cm^{-1} region, fitted to integrated band intensities derived by spectral fitting using Gaussian functions centred at the wavenumbers stated in the inset.

A)

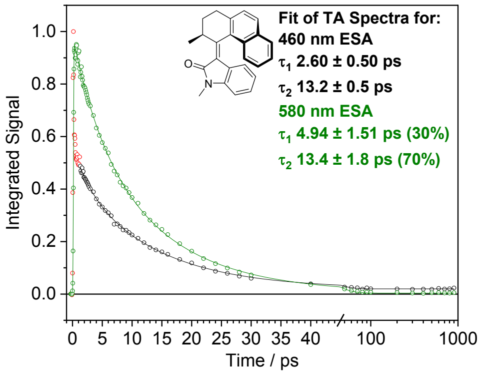

B)

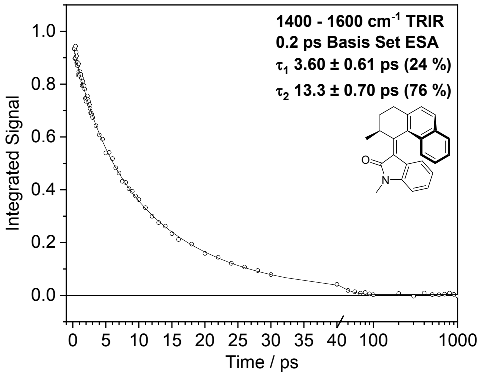

C)

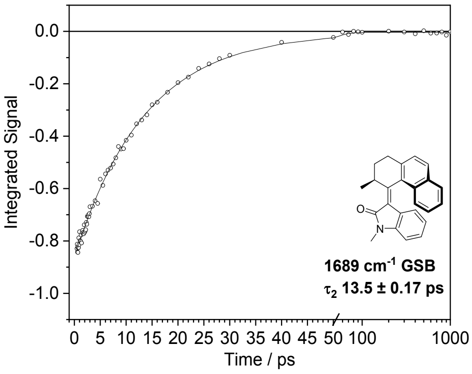

Figure S32: **A)** Excited-state kinetics deduced from the TA spectra of **2** acquired in DMSO, highlighting bi-exponential decay kinetics fitted to integrated band intensities obtained by spectral decomposition using the two Gaussian functions centred at the wavelengths stated in the inset. The < 1.3 ps 470 nm data are omitted from the fitting procedure due to non-exponential decay kinetics caused by ultrafast dynamics in the S_1 adiabatic potential. **B)** Bi-exponential excited-state kinetics of **2** from TRIR spectra in DMSO-d_6 collected in the 1400 – 1600 cm^{-1} region. These kinetics were obtained using the integrated band intensities acquired by spectral fitting using the 0.2 ps spectra as a basis function. **C)** Kinetics of the carbonyl GSB band obtained from 1600 – 1800 cm^{-1} region, fitted to integrated band intensities derived by spectral fitting using a Gaussian function centred at 1689 cm^{-1}.

A)

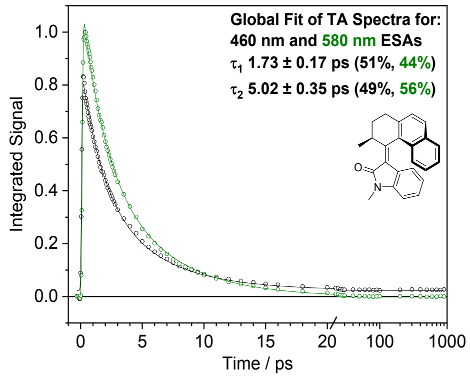

B)

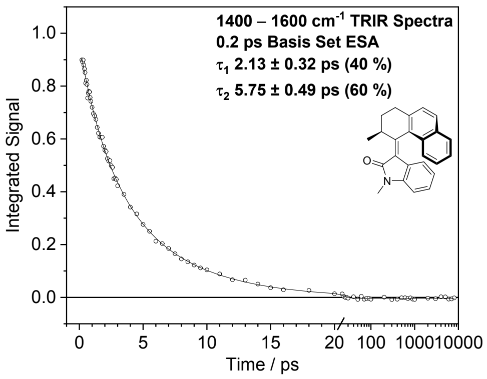

C)

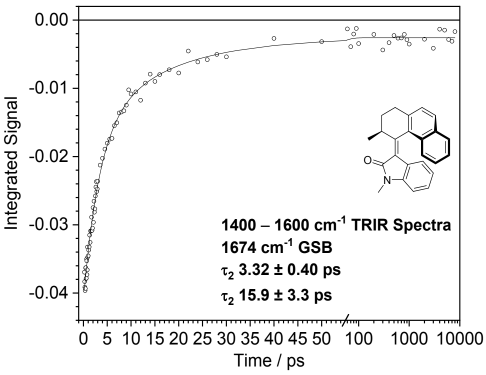

Figure S33: **A)** Excited-state kinetics deduced from the TA spectra of **2** acquired in methanol, highlighting bi-exponential decay kinetics fitted to integrated band intensities obtained by spectral decomposition using the two Gaussian functions centred at the wavelengths stated in the inset. **B)** Bi-exponential excited-state kinetics of **2** from TRIR spectra in methanol-d_4 collected in the 1400 – 1600 cm^{-1} region. These kinetics were obtained using the integrated band intensities acquired by spectral fitting using the 0.2 ps spectra as a basis function. **C)** Kinetics of the carbonyl GSB band obtained from 1400 – 1600 cm^{-1} region, fitted to integrated band intensities derived by spectral fitting using a Gaussian function centred at 1674 cm^{-1}. The deduced τ_1 = 3.32 ± 0.40 ps time constant appears anomalous. However, comparison to the average excited-state lifetime τ_{AV} = 3.36 ± 0.53 ps, calculated using the TA kinetic data, shows that this is likely an average of the excited-state decay kinetics. The data were also fitted using a tri-exponential decay function, but this fitting procedure was unsuccessful.

S9.3 Motor 3 in cyclohexane, DMSO and methanol solutions

A)

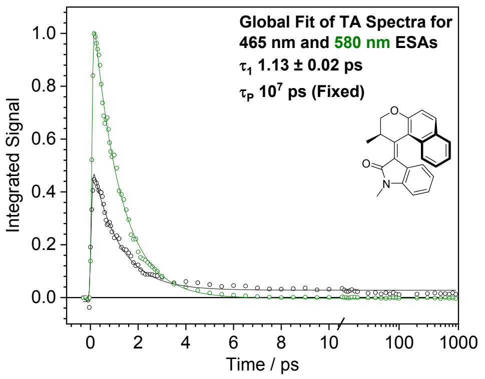

B)

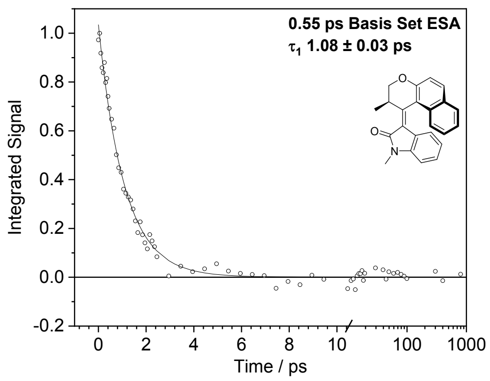

C)

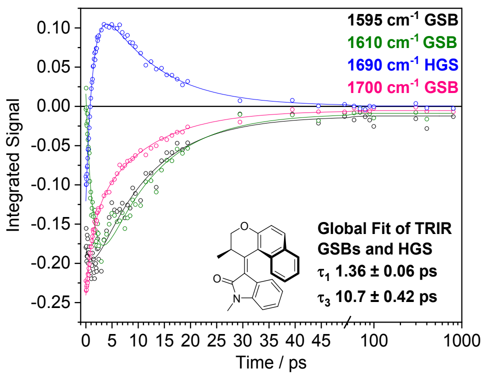

Figure S34: **A)** Excited-state kinetics deduced from the TA spectra of **3** acquired in cyclohexane, highlighting mono-exponential decay kinetics fitted to integrated band intensities obtained by spectral decomposition using the two Gaussian functions centred at the wavelengths stated in the inset. **B)** Mono-exponential excited-state kinetics of **3** from TRIR spectra in cyclohexane-d_{12} collected in the 1400 – 1600 cm^{-1} region. These kinetics were obtained using the integrated band intensities acquired by spectral fitting using the 0.55 ps spectra as a basis function. **C)** Kinetics of the GSB and HGS bands obtained from 1400 – 1600 cm^{-1} region, fitted to integrated band intensities derived by spectral fitting using Gaussian functions centred at the wavenumbers stated in the inset.

A)

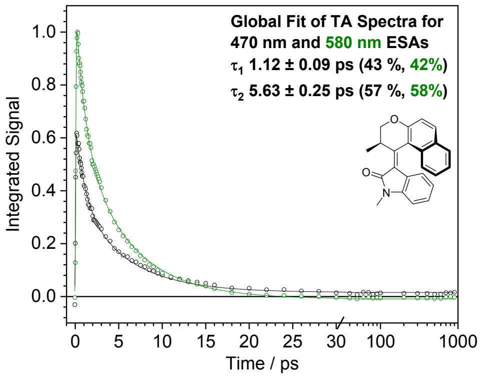

B)

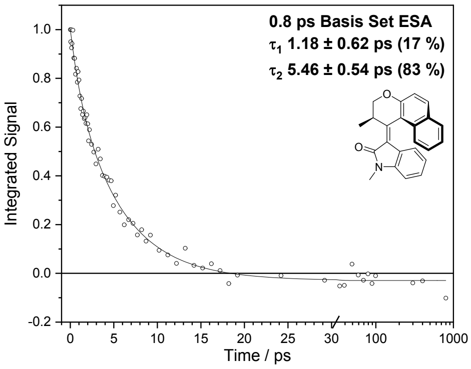

C)

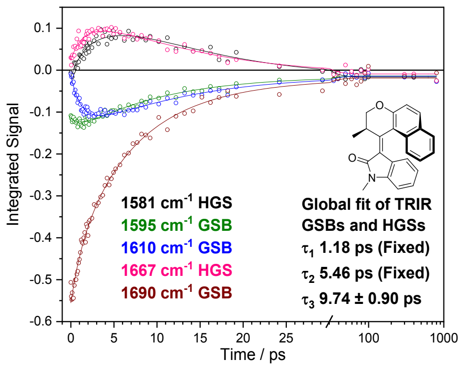

Figure S35: **A)** Excited-state kinetics deduced from the TA spectra of **3** acquired in DMSO, highlighting bi-exponential decay kinetics fitted to integrated band intensities obtained by spectral decomposition using the two Gaussian functions centred at the wavelengths stated in the inset. **B)** Bi-exponential excited-state kinetics of **3** from TRIR spectra in DMSO-d_6 collected in the 1400 – 1600 cm^{-1} region. These kinetics were obtained using the integrated band intensities acquired by spectral fitting using the 0.8 ps spectrum as a basis function. **C)** Kinetics of the GSB and HGS bands obtained from 1400 – 1600 cm^{-1} region, fitted to integrated band intensities derived by spectral fitting using Gaussian functions centred at the wavenumbers stated in the inset.

A)

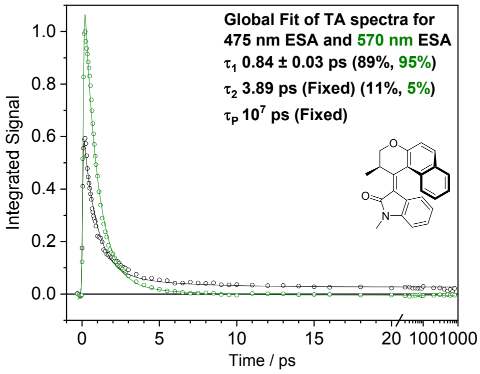

B)

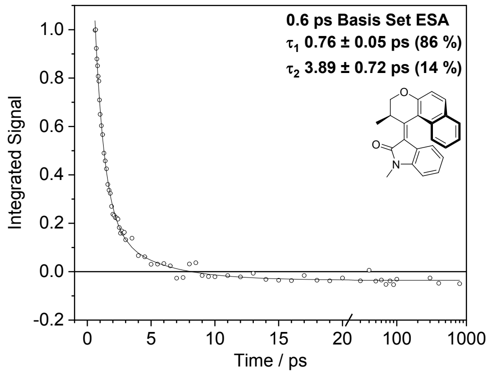

C)

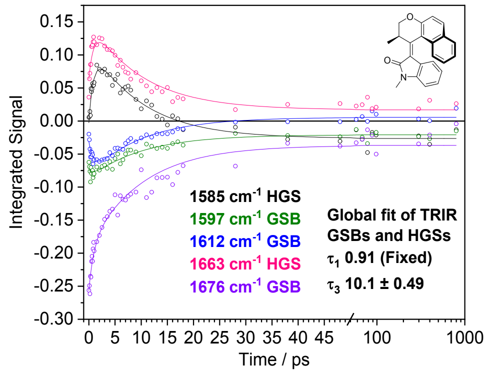

Figure S36: **A)** Excited-state kinetics deduced from the TA spectra of **3** acquired in methanol, highlighting bi-exponential decay kinetics fitted to integrated band intensities obtained by spectral decomposition using the two Gaussian functions centred at the wavelengths stated in the inset. **B)** Bi-exponential excited-state kinetics of **3** from TRIR spectra in methanol-d_4 collected in the 1400 – 1600 cm^{-1} region. These kinetics were obtained using the integrated band intensities acquired by spectral fitting using the 0.6 ps spectrum as a basis function. **C)** Kinetics of the GSB and HGS bands obtained from 1400 – 1600 cm^{-1} region, fitted to integrated band intensities derived by spectral fitting using Gaussian functions centred at the wavenumbers stated in the inset.

S9.4 Motor 4 in cyclohexane, DMSO and methanol solutions

A)

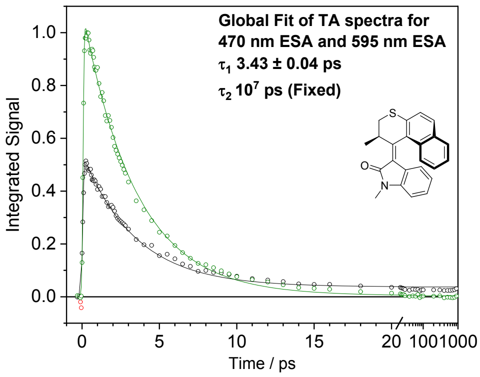

B)

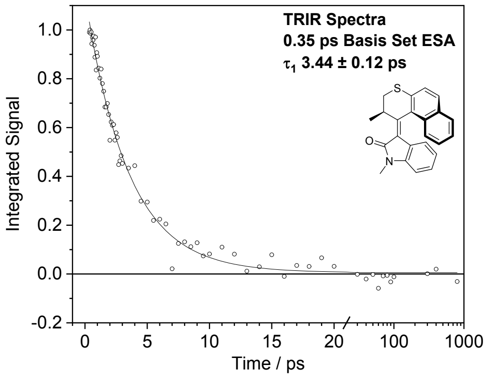

C)

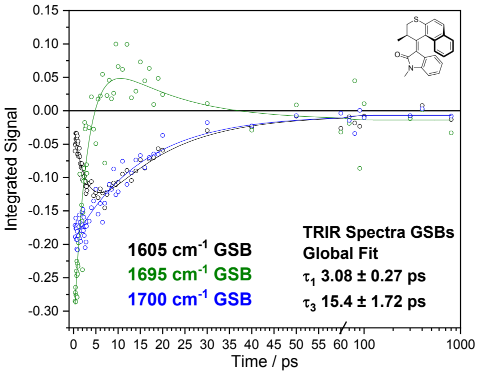

Figure S37: **A)** Excited-state kinetics deduced from the TA spectra of **4** acquired in cyclohexane, highlighting mono-exponential decay kinetics fitted to integrated band intensities obtained by spectral decomposition using the two Gaussian functions centred at the wavelengths stated in the inset. **B)** Mono-exponential excited-state kinetics of **4** from TRIR spectra in cyclohexane-d_{12} collected in the 1400 – 1600 cm^{-1} region. These kinetics were obtained using the integrated band intensities acquired by spectral fitting using the 0.35 ps spectrum as a basis function. **C)** Kinetics of the GSB bands obtained from 1400 – 1600 cm^{-1} region, fitted to integrated band intensities derived by spectral fitting using Gaussian functions centred at the wavenumbers stated in the inset.

A)

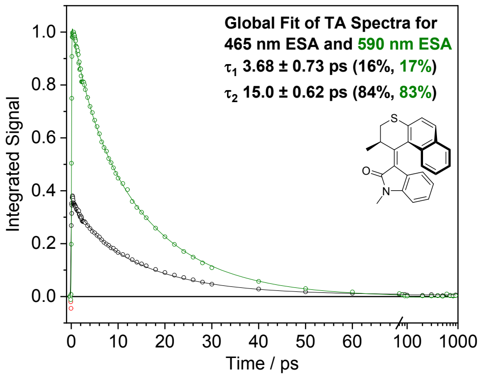

B)

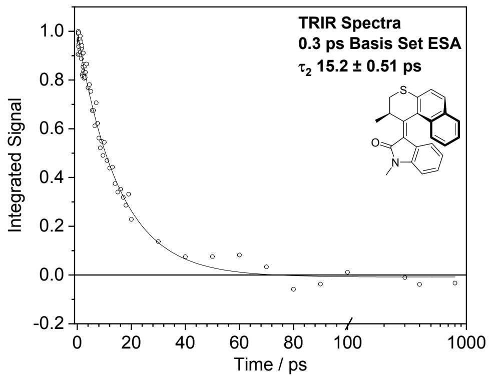

C)

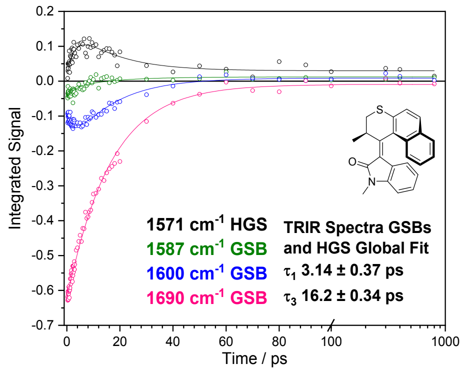

Figure S38: **A)** Excited-state kinetics deduced from the TA spectra of **4** acquired in DMSO, highlighting bi-exponential decay kinetics fitted to integrated band intensities obtained by spectral decomposition using the two Gaussian functions centred at the wavelengths stated in the inset. **B)** Bi-exponential excited-state kinetics of **4** from TRIR spectra in DMSO-d_6 collected in the 1400 – 1600 cm^{-1} region. These kinetics were obtained using the integrated band intensities acquired by spectral fitting using the 0.3 ps spectrum as a basis function. **C)** Kinetics of the GSB and HGS bands obtained from 1400 – 1600 cm^{-1} region, fitted to integrated band intensities derived by spectral fitting using Gaussian functions centred at the wavenumbers stated in the inset.

A)

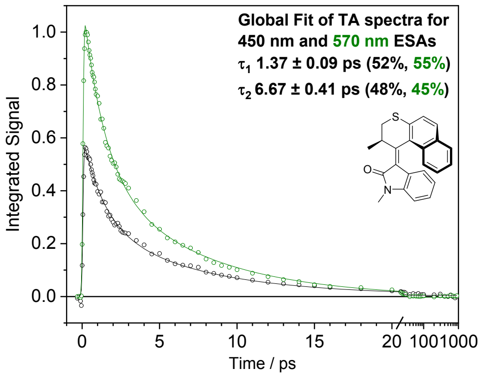

B)

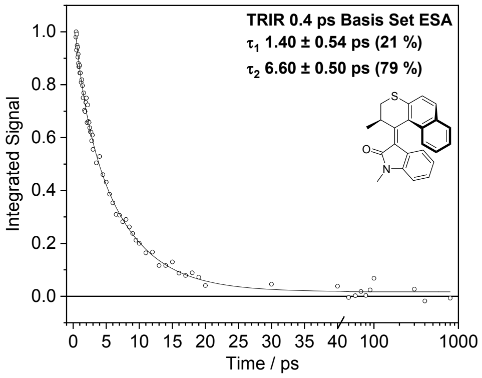

C)

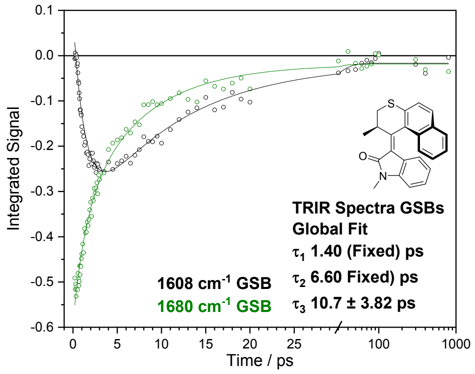

Figure S39: Excited-state kinetics deduced from the TA spectra of **4** acquired in methanol, highlighting bi-exponential decay kinetics fitted to integrated band intensities obtained by spectral decomposition using the two Gaussian functions centred at the wavelengths stated in the inset. **B)** Bi-exponential excited-state kinetics of **4** from TRIR spectra in methanol-d_4 collected in the 1400 – 1600 cm^{-1} region. These kinetics were obtained using the integrated band intensities acquired by spectral fitting using the 0.4 ps spectrum as a basis function. **C)** Kinetics of the GSB bands obtained from 1400 – 1600 cm^{-1} region, fitted to integrated band intensities derived by spectral fitting using Gaussian functions centred at the wavenumbers stated in the inset.

S9.5 Motor 5 in cyclohexane, DMSO and methanol solutions

A)

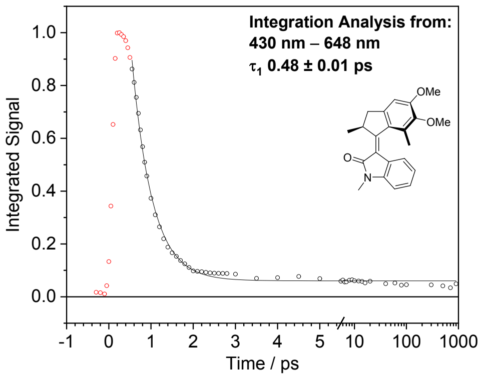

B) **C)**

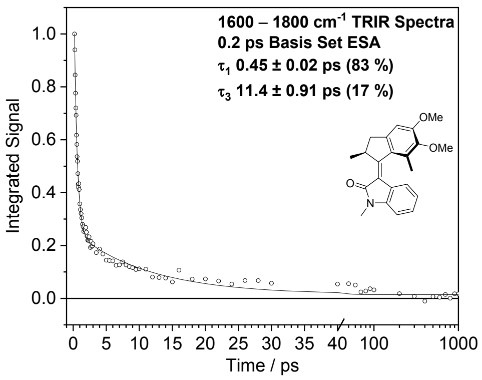

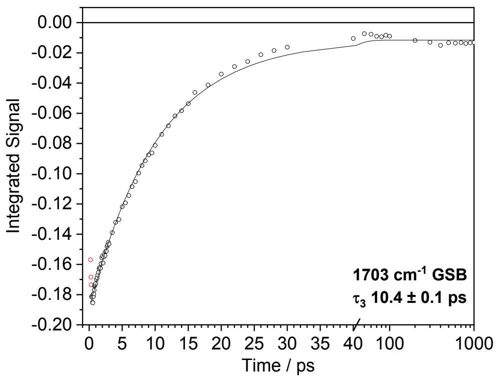

Figure S40: **A)** Excited-state kinetics deduced from the TA spectra of **5** acquired in cyclohexane, highlighting mono-exponential decay kinetics obtained using the integrated signal from 430 – 648 nm. The < 0.55 ps data (red circles) are omitted from the fitting procedure due to non-exponential decay kinetics caused by ultrafast kinetics in the S_1 adiabatic potential. **B)** Bi-exponential excited-state kinetics of **5** from TRIR spectra in cyclohexane-d_{12} collected in the 1600 – 1800 cm^{-1} region. These kinetics were obtained using the integrated band intensities acquired by fitting of the spectral region using the 0.2 ps spectrum as a basis function. **C)** Kinetics of the carbonyl GSB band obtained from 1600 – 1800 cm^{-1} region, fitted to integrated band intensities derived by spectral fitting using a Gaussian function centred at 1703 cm^{-1}.

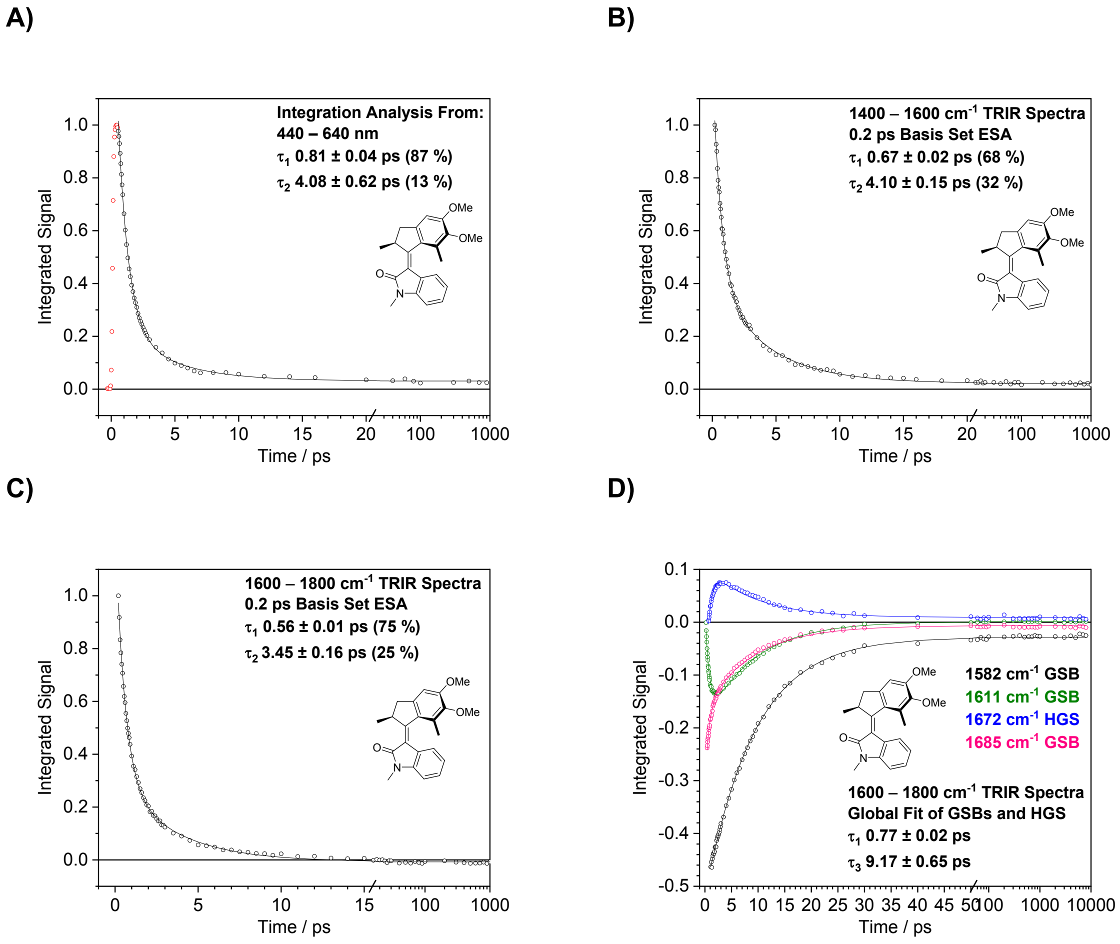

Figure S41: **A)** Excited-state kinetics deduced from the TA spectra of **5** acquired in DMSO, highlighting bi-exponential decay kinetics obtained using the integrated signal from 440 – 640 nm. The < 0.55 ps data (red circles) are omitted from the fitting procedure due to non-exponential decay kinetics caused by ultrafast dynamics in the S_1 adiabatic potential. **B)** and **C)** Bi-exponential excited-state kinetics of **5** from TRIR spectra in DMSO-d_6 collected in the 1400 – 1600 cm^{-1} and 1600 – 1800 cm^{-1} regions, respectively. These kinetics were obtained using the integrated band intensities acquired by fitting of each spectral region using their respective 0.2 ps spectrum as a basis function. **D)** Kinetics of the GSB and HGS bands obtained from 1600 – 1800 cm^{-1} region, fitted to integrated band intensities derived by spectral fitting using Gaussian functions centred at the wavenumbers stated in the inset.

A)

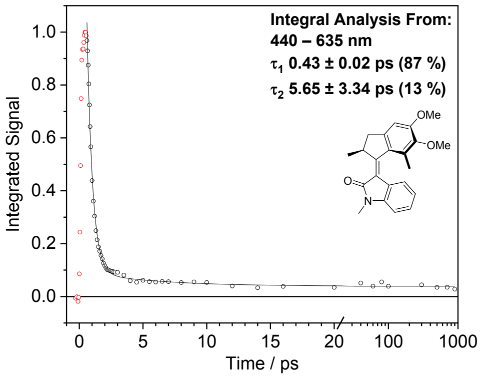

B)

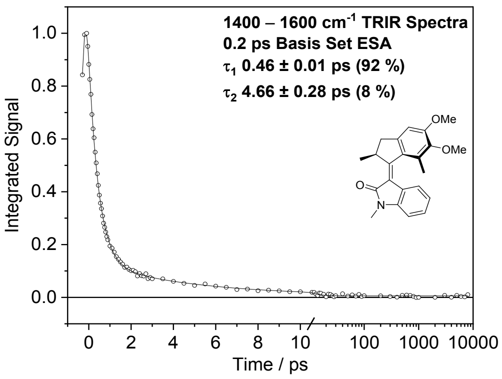

C)

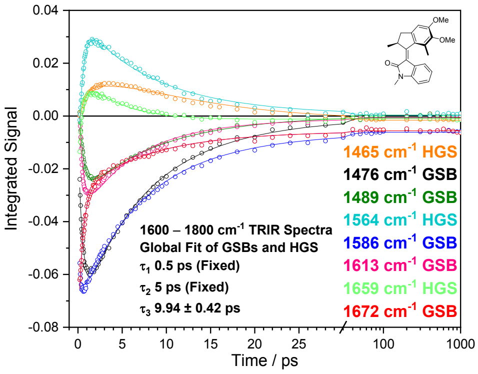

Figure S42: **A)** Excited-state kinetics deduced from the TA spectra of **5** acquired in methanol, highlighting bi-exponential decay kinetics acquired using the integrated signal from 440 – 635 nm. The < 0.6 ps data (red circles) are omitted from the fitting procedure due to non-exponential decay kinetics caused by ultrafast dynamics in the S_1 adiabatic potential. **B)** Bi-exponential excited-state kinetics of **5** from TRIR spectra in DMSO-d_6 collected in the 1600 – 1800 cm^{-1} region. These kinetics were obtained using the integrated band intensities acquired by fitting of the spectral region using the 0.2 ps spectrum as a basis function. **C)** Kinetics of the GSB and HGS bands obtained from 1600 – 1800 cm^{-1} region, fitted to integrated band intensities derived by spectral fitting using Gaussian functions centred at the wavenumbers stated in the inset.

S10 Summary of the Measured Excited-State Kinetics of the Oxindole Motors

Table S2: Summary of the excited-state kinetics of motor **1** after excitation with 375 nm light in three solvents: cyclohexane, DMSO and methanol. The percentages express the proportion of the dark state that decays *via* directed motion (τ_1) or indirect motion (τ_2) towards the S_1/S_0 CI. These percentages are calculated from the ratio of the preexponential factors for τ_1 and τ_2 decay components, which quantify the bi-exponential lifetimes of the dark state. The average dark state lifetime ($< \tau_{ds} >$) is calculated from the weighted average of the τ_1 and τ_2 time constants.

		τ_1 / ps	τ_2 / ps	τ_3 / ps	$< \tau_{ds} >$ / ps
Cyclohexane	TA	1.16 ± 0.02	–	–	–
	TRIR	1.11 ± 0.01[a]<br>1.13 ± 0.01[b]	–<br>–	12.7 ± 1.00[a]<br>13.6 ± 0.24[b]	–<br>–
DMSO	TA	1.60 ± 0.10 (61%)	5.76 ± 0.52 (39%)	–	3.21 ± 0.42
	TRIR	1.65 ± 0.07[a] (52%)<br>1.63 ± 0.05[b] (62%)	6.58 ± 0.25[a] (48%)<br>5.38 ± 0.06[b] (38%)	–<br>11.4 ± 0.10[b†]	3.10 ± 0.12[a]<br>3.43 ± 0.10[b]
Methanol	TA	0.73 ± 0.02 (89%)	5.12 ± 0.54 (11%)	–	1.22 ± 0.09
	TRIR	0.78 ± 0.01[a] (84%)	4.95 ± 0.26[a] (16%)	9.06 ± 0.13[a†]	1.47 ± 0.06[a]

[(a)] ESA lifetime from TRIR spectra recorded in the 1400 – 1600 cm^{-1} spectral window. [(b)] ESA lifetime from TRIR spectra recorded in the 1600 – 1800 cm^{-1} spectral window. [(†)] Lifetime derived from global fitting of TRIR GSB and HGS absorptions.

Table S3: Summary of the excited-state kinetics of motor **2** after excitation with 345 nm light in three solvents: cyclohexane, DMSO and methanol. The percentages express the proportion of the dark state that decays *via* directed motion (τ_1) or indirect motion (τ_2) towards the S_1/S_0 CI. These percentages are calculated from the ratio of the preexponential factors for τ_1 and τ_2 decay components, which quantify the bi-exponential lifetimes of the dark state. The average dark state lifetime ($< \tau_{ds} >$) is calculated from the weighted average of the τ_1 and τ_2 time constants.

		τ_1 / ps	τ_2 / ps	τ_3 / ps	$< \tau_{ds} >$ / ps
Cyclohexane	TA	4.01 ± 0.07	–	–	–
	TRIR	3.99 ± 0.12[a]<br>4.08 ± 0.13[b]	–<br>–	20.3 ± 5.3[a]<br>25.4 ± 12.0[b]	–<br>–
DMSO	TA	4.94 ± 1.51 (30%)	13.4 ± 1.8 (70%)	–	10.8 ± 3.7
	TRIR	3.60 ± 0.61[a] (24%)	13.3 ± 0.70[a] (76%)	–	10.9 ± 1.22[a]
Methanol	TA	1.73 ± 0.17 (44%)	5.02 ± 0.35 (56%)	–	3.58 ± 0.54
	TRIR	2.13 ± 0.32[a] (40%)	5.75 ± 0.49[a] (60%)	15.9 ± 3.3[a†]	4.32 ± 0.92[a]

[(a)] ESA lifetime from TRIR spectra recorded in the 1400 – 1600 cm^{-1} spectral window. [(b)] ESA lifetime from TRIR spectra recorded in the 1600 – 1800 cm^{-1} spectral window. [(†)] Lifetime derived from global fitting of TRIR GSB and HGS absorptions.

Table S4: Summary of the excited-state kinetics of motor **3** after excitation with 410 nm light in three solvents: cyclohexane, DMSO and methanol. The percentages express the proportion of the dark state that decays *via* directed motion (τ_1) or indirect motion (τ_2) towards the S_1/S_0 CI. These percentages are calculated from the ratio of the preexponential factors for τ_1 and τ_2 decay components, which quantify the bi-exponential lifetimes of the dark state. The average dark state lifetime ($< \tau_{ds} >$) is calculated from the weighted average of the τ_1 and τ_2 time constants.

		τ_1 / ps	τ_2 / ps	τ_3 / ps	$< \tau_{ds} >$ / ps
Cyclohexane	TA	1.13 ± 0.02	–	–	–
	TRIR	1.08 ± 0.03[a]	–	10.7 ± 0.42[a]	–
DMSO	TA	1.12 ± 0.09 (42%)	5.63 ± 0.25 (58%)	–	3.76 ± 0.26
	TRIR	1.18 ± 0.62[a] (17%)	5.46 ± 0.54[a] (83%)	9.74 ± 0.90[a†]	4.74 ± 0.88[a]
Methanol	TA	0.84 ± 0.03 (54%)	3.89[c] (46%)	–	0.89 ± 0.07
	TRIR	0.76 ± 0.05[a] (86%)	3.89 ± 0.72[a] (14%)	10.9 ± 0.5[a†]	1.19 ± 0.16[a]

[(a)] ESA lifetime from TRIR spectra recorded in the 1400 – 1600 cm^{-1} spectral window. [(b)] ESA lifetime from TRIR spectra recorded in the 1600 – 1800 cm^{-1} spectral window. [(c)] Fixed time constant using the value obtained from the TRIR data. [(†)] Lifetime derived from global fitting of TRIR GSB and HGS absorptions.

Table S5: Summary of the excited-state kinetics of motor **4** after excitation with 410 nm light in three solvents: cyclohexane, DMSO and methanol. The percentages express the proportion of the dark state that decays *via* directed motion (τ_1) or indirect motion (τ_2) towards the S_1/S_0 CI. These percentages are calculated from the ratio of the preexponential factors for τ_1 and τ_2 decay components, which quantify the bi-exponential lifetimes of the dark state. The average dark state lifetime ($< \tau_{ds} >$) is calculated from the weighted average of the τ_1 and τ_2 time constants.

		τ_1 / ps	τ_2 / ps	τ_3 / ps	$< \tau_{ds} >$ / ps
Cyclohexane	TA	3.43 ± 0.04	–	–	–
	TRIR	3.44 ± 0.12[a]	–	15.4 ± 1.72[a]	–
DMSO	TA	3.68 ± 0.73 (17%)	15.0 ± 0.62 (83%)	–	13.1 ± 1.1
	TRIR	–	15.2 ± 0.51[a]	16.2 ± 0.3[a†]	–
Methanol	TA	1.37 ± 0.09 (55%)	6.67 ± 0.41 (45%)	–	3.75 ± 0.33
	TRIR	1.40 ± 0.54[a] (21%)	6.60 ± 0.50[a] (79%)	10.7 ± 3.8[a†]	5.50 ± 0.78[a]

[(a)] ESA lifetime from TRIR spectra recorded in the 1400 – 1600 cm^{-1} spectral window. [(b)] ESA lifetime from TRIR spectra recorded in the 1600 – 1800 cm^{-1} spectral window. [(c)] Fixed time constant using the value obtained from the TRIR data. [(†)] Lifetime derived from global fitting of TRIR GSB and HGS absorptions.

Table S6: Summary of the excited-state kinetics of motor **5** after excitation with 375 nm light in three solvents: cyclohexane, DMSO and methanol. The percentages express the proportion of the dark state that decays *via* directed motion (τ_1) or indirect motion (τ_2) towards the S_1/S_0 CI. These percentages are calculated from the ratio of the preexponential factors for τ_1 and τ_2 decay components, which quantify the bi-exponential lifetimes of the dark state. The average dark state lifetime ($< \tau_{ds} >$) is calculated from the weighted average of the τ_1 and τ_2 time constants.

		τ_1 / ps	τ_2 / ps	τ_3 / ps	$< \tau_{ds} >$ / ps
Cyclohexane	TA	0.48 ± 0.01	–	–	–
	TRIR	0.45 ± 0.02[b]	–	11.4 ± 0.91[a]	–
DMSO	TA	0.81 ± 0.04 (87%)	4.08 ± 0.62 (13%)	–	1.25 ± 0.13
	TRIR	0.67 ± 0.02[a] (68%)<br>0.56 ± 0.01[b] (75%)	4.10 ± 0.15[a] (32%)<br>3.45 ± 0.16[b] (25%)	9.17 ± 0.65[a†]<br>–	1.79 ± 0.07<br>1.27 ± 0.06
Methanol	TA	0.43 ± 0.02 (87%)	5.65 ± 3.34 (13%)	–	0.51 ± 0.07
	TRIR	0.46 ± 0.01[a] (92%)	4.66 ± 0.28[a] (8%)	9.94 ± 0.42[b†]	0.79 ± 0.03[a]

[(a)] ESA lifetime from TRIR spectra recorded in the 1400 – 1600 cm^{-1} spectral window. [(b)] ESA lifetime from TRIR spectra recorded in the 1600 – 1800 cm^{-1} spectral window. [(c)] Fixed time constant using the value obtained from the TRIR data. [(†)] Lifetime derived from global fitting of TRIR GSB and HGS absorptions.

S11 Fitted Oscillatory Dynamics of the TA ESA Bands

All the integrated signals plotted in this section are intensities integrated over the full range of wavelengths of the bands after spectral decomposition. The inset labels in figure panels identify the excited-state absorption bands.

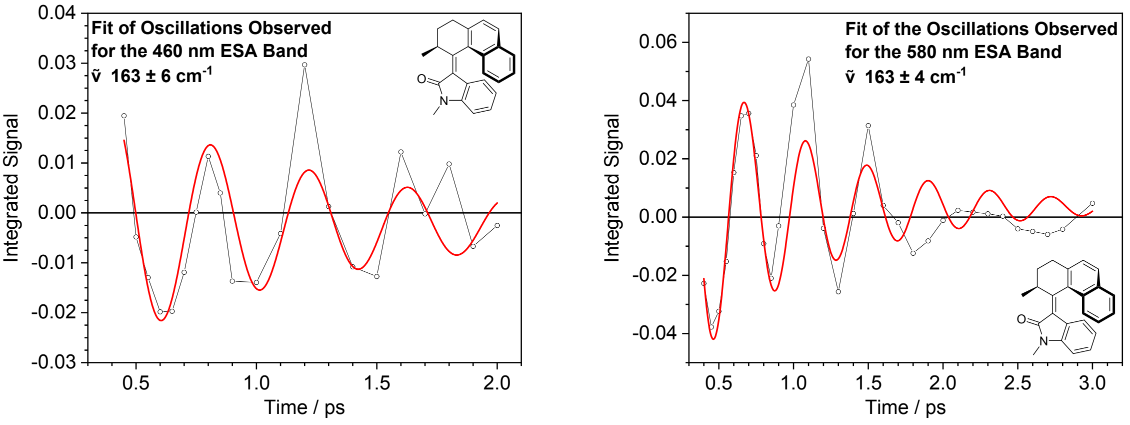

Figure S43: Fitted oscillatory dynamics of motor **2** in DMSO using a damped sine function.

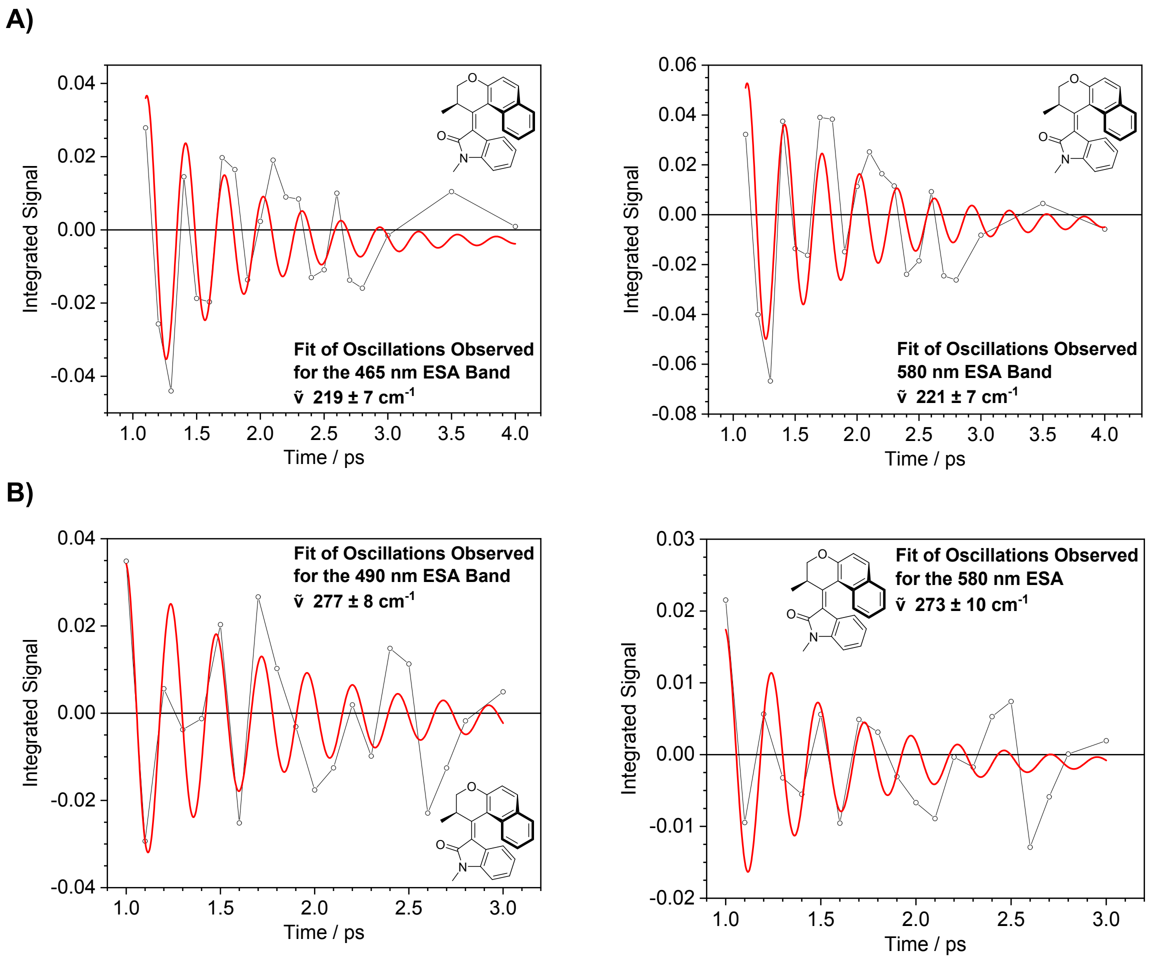

Figure S44: Fitted oscillatory dynamics of motor **3** in **A)** cyclohexane and **B)** DMSO using a damped sine function.

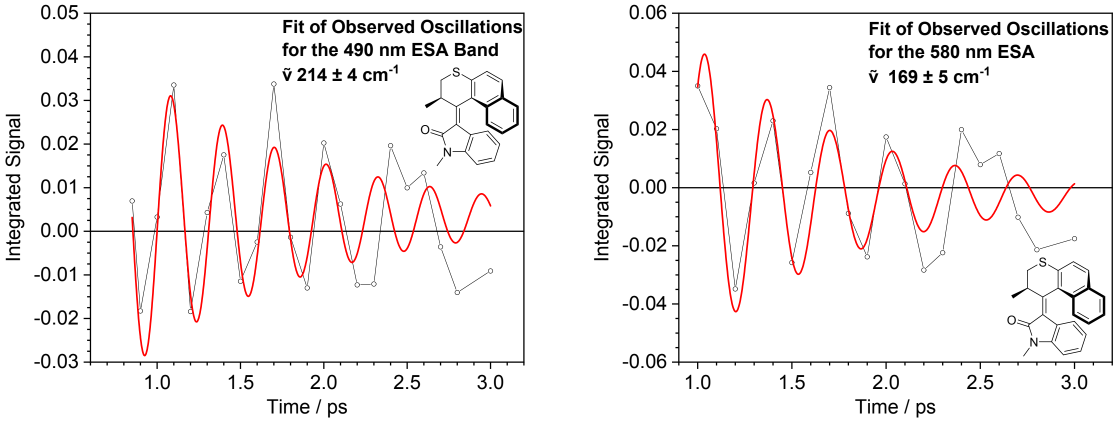

Figure S45: Fitted oscillatory dynamics of motor **3** in cyclohexane using a damped sine function.

S12 Characterization of the Excited-State PESs Using TD-DFT Calculations of Interpolated Oxindole Motor Structures

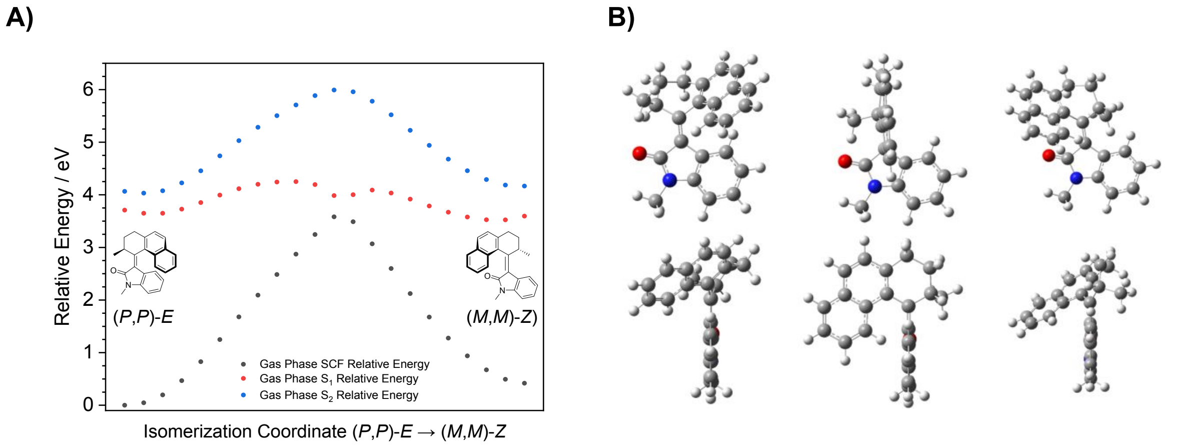

Figure S46: **A)** Relative energies calculated at the ωB97-XD/6-31+G(d,p) level of theory for the interpolated structures of motor **2** in the S_0 (black), S_1 (red) and S_2 (blue) electronic states. Calculations were performed for the gas phase. **B)** Structures of the optimized (*P*,*P*)-*E*-**2** diastereomer, the interpolated isomerization saddle point on S_0 and the optimized (*M*,*M*)-*Z*-**2** diastereomer. In each case, two views of the same structure are shown.

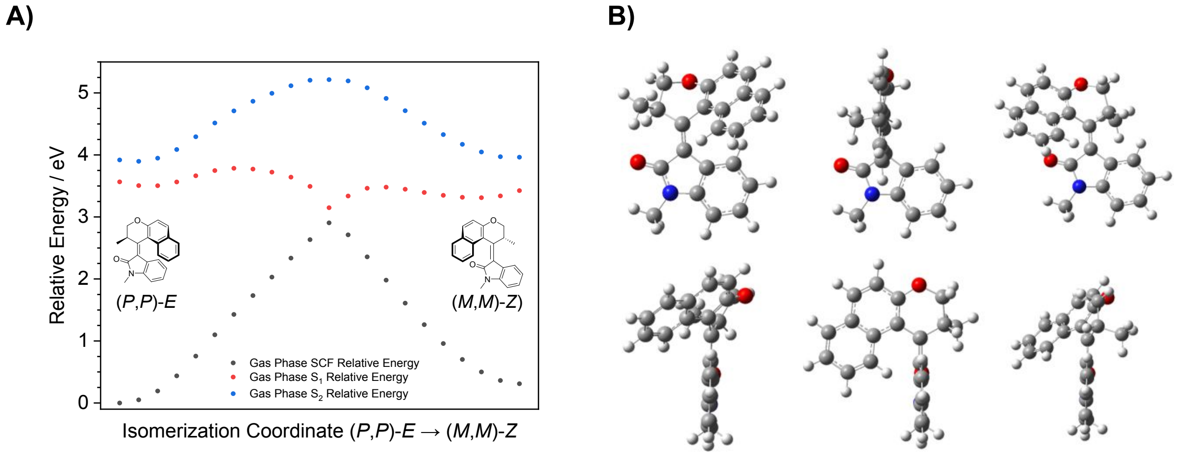

Figure S47: **A)** Relative energies calculated at the ωB97-XD/6-31+G(d,p) level of theory for the interpolated structures of motor **3** in the S_0 (black), S_1 (red) and S_2 (blue) electronic states. Calculations were performed for the gas phase. **B)** Structures of the optimized (*P*,*P*)-*E*-**3** diastereomer, the interpolated isomerization saddle point on S_0 and the optimized (*M*,*M*)-*Z*-**3** diastereomer. In each case, two views of the same structure are shown.

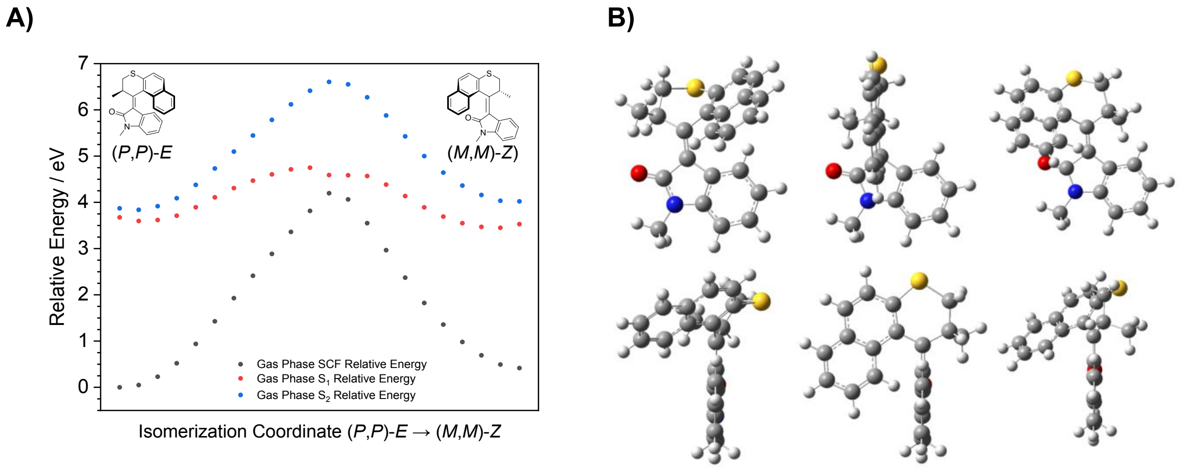

Figure S48: **A)** Relative energies calculated at the ωB97-XD/6-31+G(d,p) level of theory for the interpolated structures of motor **4** in the S_0 (black), S_1 (red) and S_2 (blue) electronic states. Calculations were performed for the gas phase. **B)** Structures of the optimized (*P*,*P*)-*E*-**4** diastereomer, the interpolated isomerization saddle point on S_0 and the optimized (*M*,*M*)-*Z*-**4** diastereomer. In each case, two views of the same structure are shown.

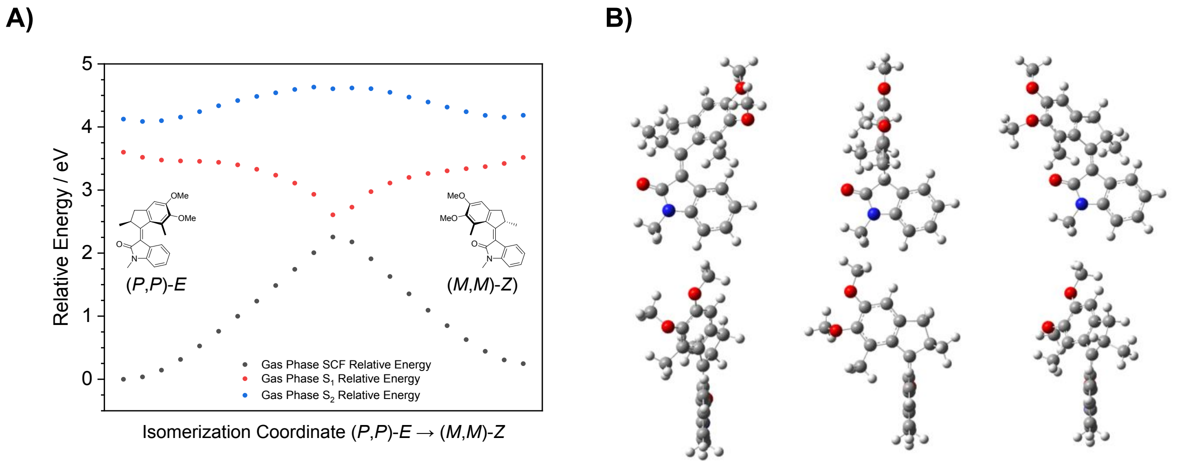

Figure S49: **A)** Relative energies calculated at the ωB97-XD/6-31+G(d,p) level of theory for the interpolated structures of motor **5** in the S_0 (black), S_1 (red) and S_2 (blue) electronic states. Calculations were performed for the gas phase. **B)** Structures of the optimized (*P*,*P*)-*E*-**5** diastereomer, the interpolated isomerization saddle point on S_0 and the optimized (*M*,*M*)-*Z*-**5** diastereomer. In each case, two views of the same structure are shown.

S13 References

1 ter Wiel, M. K. J.; van Delden, R. A.; Meetsma, A.; Feringa, B. L., Increased Speed of Rotation for the Smallest Light-Driven Molecular Motor, *J Am Chem Soc*, **2003**, *125*, 15076–15086

2 Štacko, P.; Kistemaker, J. C. M.; Feringa, B. L., Fluorine-Substituted Molecular Motors with a Quaternary Stereogenic Center, *Chem Eur J*, **2017**, *23*, 6643–6653.

3 Roke, D.; Sen, M.; Danowski, W.; Wezenberg, S. J.; Feringa, B. L., Visible-Light-Driven Tunable Molecular Motors Based on Oxindole, *J Am Chem Soc*, **2019**, *141*, 7622–7627.

4 Harada, N.; Koumura, N.; Feringa, B. L., Chemistry of Unique Chiral Olefins. 3. Synthesis and Absolute Stereochemistry of *trans*- and *cis*-1,1‘,2,2‘,3,3‘,4,4‘- Octahydro-3,3‘-dimethyl-4,4‘-biphenanthrylidenes, *J Am Chem Soc*, **1997**, *119*, 7256–7264.

5 Gawade, P. M.; Khose, V. N.; Badani, P. M.; Hasan, M.; Kaabel, S.; Mobin, S. M.; Borovkov, V.; Karnik, A. V., Benzyne-Mediated Nonconcerted Pathway toward Synthesis of Sterically Crowded [5]- and [7]-Oxahelicenoids, Stereochemical and Theoretical Studies, and Optical Resolution of Helicenoids, *J Org Chem*, **2019**, *84*, 860–868.

6 Kistemaker, J. C. M.; Pizzolato, S. F.; van Leeuwen, T.; Pijper, T. C.; Feringa, B. L., Spectroscopic and Theoretical Identification of Two Thermal Isomerization Pathways for Bistable Chiral Overcrowded Alkenes, *Chem Eur J*, **2016**, *22*, 13478–13487.

7 Pooler, D. R. S.; Pierron, R.; Crespi, S.; Costil, R.; Pfeifer, L.; Léonard, J.; Olivucci, M.; Feringa, B. L., Effect of charge-transfer enhancement on the efficiency and rotary mechanism of an oxindole-based molecular motor, *Chem Sci*, **2021**, *12*, 7486–7497.

8 Roberts, G. M.; Marroux, H. J. B.; Grubb, M. P.; Ashfold, M. N. R.; Orr-Ewing, A. J., On the Participation of Photoinduced N–H Bond Fission in Aqueous Adenine at 266 and 220 nm: A Combined Ultrafast Transient Electronic and Vibrational Absorption Spectroscopy Study, *J Phys Chem A*, **2014**, *118*, 11211–11225.

9 Grubb, M. P.; Orr-Ewing, A. J.; Ashfold, M. N. R., KOALA: A program for the processing and decomposition of transient spectra, *Rev Sci Instrumen*, **2014**, *85*, 064104

10 Origin(Pro), Version *Number* (e.g. "Version 2020b"). OriginLab Corporation, Northampton, MA, USA.

11 Frisch, M. J.; Trucks, G. W.; Schlegel, H. B.; Scuseria, G. E.; Robb, M. A.; Cheeseman, J. R.; Scalmani, G.; Barone, V.; Petersson, G. A.; Nakatsuji, H.; et al., Gaussian 16, Revision C.01, Gaussian Inc. Wallingford, CT, 2016.

12 Pang, X.; Cui, X.; Hu, D.; Jiang, C.; Zhao, D.; Lan, Z.; Li, F., “Watching” the Dark State in Ultrafast Nonadiabatic Photoisomerization Process of a Light-Driven Molecular Rotary Motor, *J Phys Chem A*, **2017**, *121*, 1240–1249.

13 Wiley, T. E.; Konar, A.; Miller, N. A.; Spears, K. G.; Sension R. J., Primed for Efficient Motion: Ultrafast Excited State Dynamics and Optical Manipulation of a Four Stage Rotary Molecular Motor, *J Phys Chem A*, **2018**, *122*, 7548–7558.

14 Amirjalayer, S.; Cnossen, A.; Browne, W. R.; Feringa, B. L.; Buma, W. J.; Woutersen, S., Direct Observation of a Dark State in the Photocycle of a Light-Driven Molecular Motor, *J Phys Chem A*, **2016**, *120*, 8606–8612.
